# Identification of Schizophrenia‐Risk Regulatory Variant rs1399178 in the Non‐coding Region With Its Impact on NRF1 Binding

**DOI:** 10.1111/cns.70275

**Published:** 2025-02-28

**Authors:** Lei Ji, Bojian Huang, Decheng Ren, Xiaoxi Wei, Liangjie Liu, Yan Bi, Zhiqiang Li, Fan Yuan, Ke Han, Keyi Li, Fengping Yang, Xingwang Li, Tao Yu, Yi Shi, Lin He, Qing Lu, Guang He

**Affiliations:** ^1^ Bio‐X Institutes, Key Laboratory for the Genetics of Developmental and Neuropsychiatric Disorders Shanghai Jiao Tong University Shanghai China; ^2^ The Collaborative Innovation Center for Brain Science, and Brain Science and Technology Research Center Shanghai Jiao Tong University Shanghai China; ^3^ Shanghai Key Laboratory of Psychotic Disorders, Shanghai Mental Health Center Shanghai Jiao Tong University School of Medicine Shanghai China; ^4^ Affiliated Hospital of Qingdao University and Biomedical Sciences Institute of Qingdao University (Qingdao Branch of SJTU Bio‐X Institutes) Qingdao University Qingdao China; ^5^ Department of Otorhinolaryngology‐Head and Neck Surgery Chongqing General Hospital Chongqing China; ^6^ Ear Institute Shanghai Jiao Tong University School of Medicine Shanghai China; ^7^ Shanghai Key Laboratory of Translational Medicine on Ear and Nose Diseases Shanghai China

**Keywords:** functional genomics, *NRF1*, rs1399178, schizophrenia, structural analysis, TF‐binding regulation

## Abstract

**Aims:**

The challenges in identifying functional variants from genome‐wide association studies (GWAS) and unraveling regulatory mechanisms in schizophrenia research persist, particularly in intronic regions. A non‐coding regulatory variant, rs1399178, associated with schizophrenia risk, is identified and its impact on NRF1 binding is investigated.

**Methods:**

This study focuses on schizophrenia GWAS risk loci, using functional genomics, expression analyses and structural analysis to identify 736 schizophrenia risk single‐nucleotide polymorphisms (SNPs) that disrupt transcription factor (TF) binding.

**Results:**

Among these SNPs, rs1399178 stands out as a bifunctional intergenic SNP that can switch between acting as a promoter and an enhancer, potentially influencing *MLH1* and *LRRFIP2* expression via expression quantitative trait loci analysis. Importantly, mutation of the G allele of rs1399178 to A significantly diminishes its binding affinity to nuclear respiratory factor 1 (NRF1). Structural analysis provides further insight into this alteration in the protein–nucleic acid complex formation.

**Conclusion:**

Based on our data, a model is proposed in which rs1399178 confers schizophrenia risk by modifying *NRF1* binding profiles, thereby regulating the abundance of target genes through promoter‐enhancer switching. This study provides novel insights into the regulatory mechanisms of schizophrenia risk variants, highlighting the intricate nature of genetic interactions and potential therapeutic targets.

## Graphical abstract

The identification of rs1399178 as a bifunctional regulatory element, mediating promoter‐enhancer switch and interacting with NRF1, sheds light on the complex regulatory networks involved in schizophrenia pathogenesis.

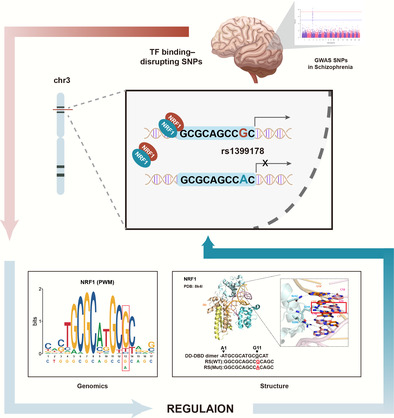

## 1 Introduction

Schizophrenia, considered one of the most severe mental disorders, is a chronic psychiatric condition with a diverse genetic and neurobiological foundation that impacts early brain development [1]. Schizophrenia is usually characterized as a combination of psychotic symptoms, including delusions, hallucinations, disordered thought, and reduced emotional expression [2, 3]. According to global estimates from the World Health Organization (WHO), ~24 million individuals (0.32% of the global population) are affected by schizophrenia, equating to approximately one in every 300 people. The global lifetime prevalence of schizophrenia is ~1% [4]. Although its prevalence is comparatively lower than other psychiatric disorders, such as major depression or bipolar disorder, schizophrenia is notable for having one of the highest global rates of disability. In fact, schizophrenia leads to significant economic costs and places a substantial mental burden on patients, their families, and society as a whole, collectively contributing to a diminished quality of life [5, 6].

Despite significant progress in schizophrenia research over the past decade, a comprehensive understanding of its pathogenesis remains elusive. Genomic studies have unveiled the intricate genetic architecture of schizophrenia, with recent emphasis on identifying rare and common risk alleles to deepen insights into its etiology. Notably, over 200 risk loci have been identified across diverse populations [7, 8, 9, 10, 11, 12, 13]. Lee et al.'s research emphasizes the significance of single‐nucleotide polymorphisms (SNPs), contributing to ~23% of the heritability of schizophrenia. This underscores the critical role of common variants in the disorder [14]. However, challenges persist in identifying functional or causal variants at reported loci and elucidating their regulatory mechanisms, mainly owing to the complexities of linkage disequilibrium (LD) and gene regulation [15, 16]. Individual risk alleles may govern multiple genes through intricate chromatin interactions, resulting in effects on genes located distally in the linear genome [17, 18]. On the other hand, the majority of risk loci identified through genome‐wide association studies (GWAS) are located in non‐coding regions [13, 19]. Bacanu et al. found that variations associated with schizophrenia exhibit significant enrichment in regulatory regions, suggesting that schizophrenia may exert its influence by disrupting regulatory function and altering gene expression [20, 21]. Luo et al. used functional genomics approaches to identify 132 schizophrenia risk variants influencing transcription factor (TF) binding [22]. Considering the presumed neurobiological origin of schizophrenia, research efforts focused on identifying risk variants regulating human brain tissue or neuronal cells [23, 24, 25]. Despite the existence of up to 1600 TFs in the human genome, Luo et al. deliberately included a limited number of TFs (34) in their study [22, 26]. However, this restriction poses challenges to discerning risk SNP loci that disrupt the binding of other TFs [22]. The proliferation of sequencing data in recent years, along with the progress of additional databases, has significantly expanded the available information during this five‐year period. Leveraging this expanded and novel data pool is poised to enhance our understanding of the regulatory mechanisms underlying schizophrenia.

Here, we systematically identify functional variants at schizophrenia risk loci derived from GWAS, and explore how these variants exert their biological effects and regulatory mechanisms in the context of schizophrenia [11]. Leveraging functional genomics and expression quantitative trait loci (eQTL) analyses, coupled with a series of functional validations, we annotated SNPs from GWAS, pinpointing schizophrenia regulatory loci. Based on the results of the reporter gene assay, we selected the rs1399178 locus for functional genomics validation, which indicated this variant disrupts the binding of NRF1. Structural evidence elucidating the molecular mechanisms behind this recognition alteration further underscored the significance of our results. Our findings highlight the intricate genetic regulatory structure of schizophrenia risk variants, providing novel insights into target genes.

## 2 Methods

### 2.1 Functional Genomics Pipelines Used to Identify Risk SNPs Affecting TF Binding

#### 2.1.1 GWAS Used in This Study

GWAS schizophrenia risk SNPs identified by Li et al. were used in this study [11]. Li et al. first carried out a GWAS approach in the Chinese population (containing 7699 cases and 18,327 controls). A total of 124 genome‐wide significant (*p* < 5 × 10^−8^) SNPs were identified based on the combined results of the trans‐ancestry meta‐analysis and replication samples.

#### 2.1.2 Extraction of SNPs in LD With the Index SNPs

For each risk locus in the East‐Asian individuals from the 1000 Genomes Project (Phase 3_Version 5) [27], we extracted SNPs that were in LD with the 124 index SNPs using the “LDlinkR” R package. All downstream analyses were performed using R v4.1.3. We selected the widely used *r* ^2^ threshold (*r* ^2^ > 0.3) following previous work [22, 28]. In total, 21,300 SNPs (including the index SNPs and SNPs in LD with the index SNPs) were obtained.

#### 2.1.3 Functional Genomics Pipelines Used to Identify Risk SNPs That Disrupt TF Binding

**FIGURE 1:**
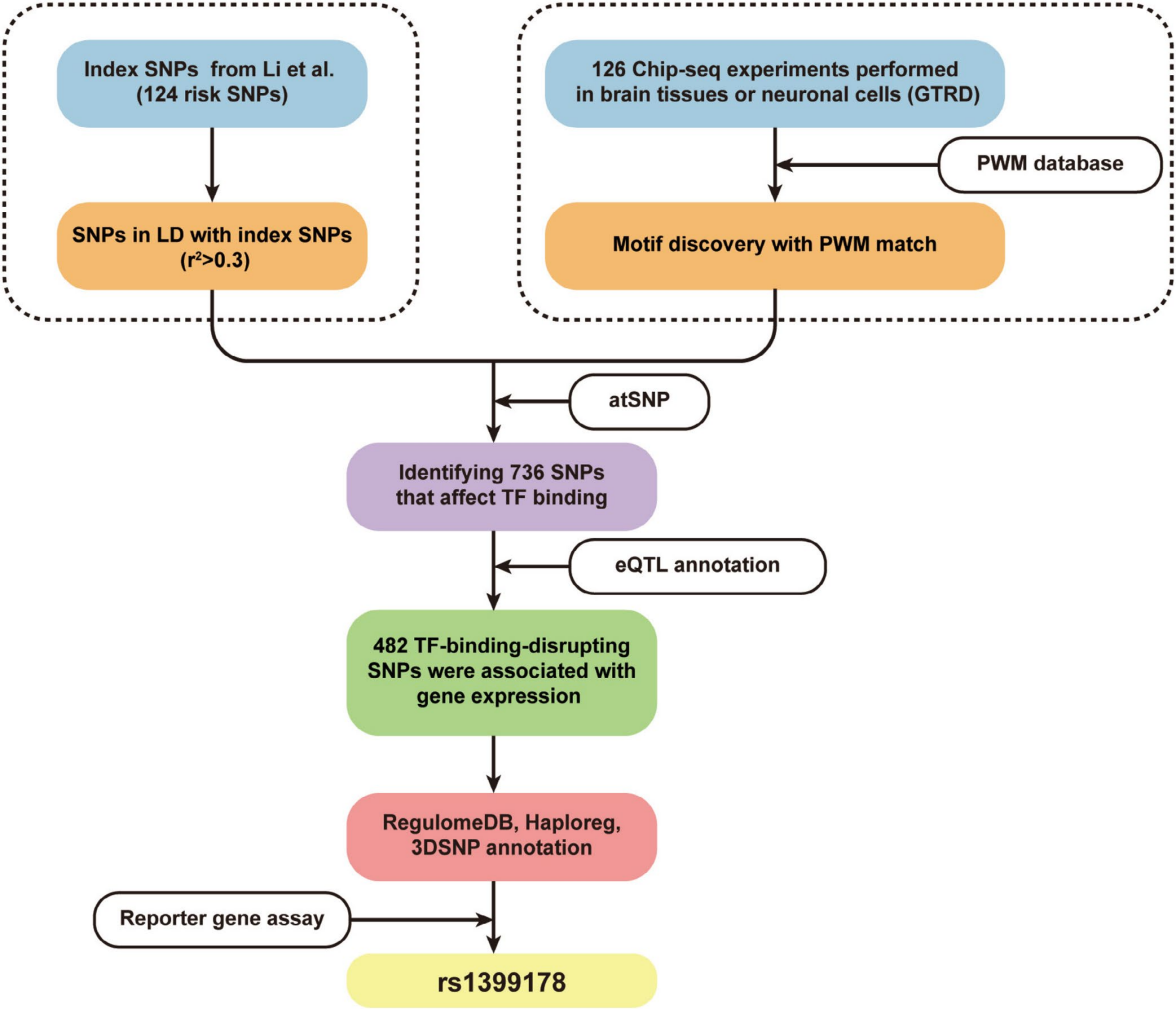
Flowchart of functional genomics analysis. ChIP‐seq assays performed using neuronal cell lines and brain tissues were used for peak calling and motif discovery. The identified motifs were then compared with the PWMs from the respective database (see Section 2), and the matched motifs were used for downstream analyses. SNPs in linkage disequilibrium (LD, *r* ^2^ ≥ 0.3) with the index SNPs (identified by SCZ GWAS, a total of 124 index SNPs) were extracted. A total of 23,000 SNPs from 124 SCZ risk loci were mapped to the identified motifs to investigate their association with the disruption of transcription factor biding. In total, 736 TF binding‐disrupting SNPs were identified.

The functional genomics flow chart for identifying TF‐disrupting SNPs is shown in Figure 1. Briefly, we first utilized 126 chromatin immunoprecipitation (ChIP)‐seq data from the Gene Transcription Regulation Database (GTRD, version 21.12), including only ChIP‐seq experiments performed in human brain tissues or neuronal cells (including neuronal cell lines and iPSCs‐derived neural progenitor cells) [29]. The detailed information about the TFs and ChIP‐seq experiments is found in Supporting Information.

To obtain DNA‐binding motifs (PWMs) enriched in the genomic sequences surrounding ChIP‐seq signals, the top 500 ChIP‐seq peaks (±20 bp, ranked by peak height) were used for motif discovery with runStreme function of the “memes” R package (with parameters “nmotifs = 5, minw = 6, maxw = 20”) [30]. Taking a shuffled set of input sequences as background, peaks with a false discovery rate (FDR) > 5% were excluded. We then examined the binding specificity of the included TFs by comparing the motifs from ChIP‐seq with the 6091 human motif sequences available from the “MotifDb” R package (created from public sources containing HOCOMOCO, JASPAR, SwissRegulon and HOMER). The matched PWMs were utilized to investigate whether the potential schizophrenia SNPs were located in the binding motif of these TFs.

To quantify the in silico effect of SNPs on TF binding, we used the “atSNP” R package to calculate P values for each SNP‐transcription factor pair (with parameters “half.window.size = 20”) [31]. For this analysis, we required a significant binding likelihood (*p* < 0.001) for at least one of the alleles (reflecting potential TF occupancy of at least one allele), and a significant difference in binding likelihood between the two alleles (*p* value_rank < 0.05 after Bonferroni correction). In total, we identified 736 SNPs that disrupted the binding of TFs (Table S1).

### 2.2 eQTL Analysis

The eQTL summary data utilized in this analysis were sourced from the eQTL Catalogue (https://www.ebi.ac.uk/eqtl/), a repository housing uniformly standardized eQTL summary data compiled from various published sources [32]. Given that schizophrenia primarily manifests as a brain condition, only eQTL datasets derived from human brain tissues were considered. Specifically, datasets from the Common Mind Consortium (*N* = 586), the Genotype‐Tissue Expression v8 (encompassing 13 brain regions, with N ranging from 114 to 209), the Religious Orders Study and Memory and Aging Project (*N* = 560), and the Braineac2 database (specifically, putamen with *N* = 102 and substantia nigra with *N* = 65) were used. Only SNPs exhibiting p‐values below 0.001 after FDR correction were considered for subsequent analysis.

### 2.3 Functional Annotation of TF Binding‐Disrupting SNPs Using RegulomeDB, Haploreg, and 3DSNP

We used three widely recognized online functional annotation tools to enrich for the most functional SNPs at each risk loci, including RegulomeDB, HaploReg v4.1, and 3DSNP v2.0 [33, 34, 35]. All TF binding‐disrupting SNPs identified in this study have been scored to evaluate the functional significance of SNPs using RegulomeDB, HaploReg, and 3DSNP, one by one. We also investigated whether the variation in rs1399178 within topologically associating domain (TAD) structures could be associated with gene regulation [17]. Briefly, we collected in situ Hi‐C data for the GM12878 (lymphoblastoid cells), IMR90 (fetal lung fibroblasts), and hESC (human embryonic stem cells) cell lines from the 4DN data portal (https://data.4dnucleome.org/), and analyzed the TAD distribution with resolutions of 50 kb [36, 37]. Detailed information is provided in Supporting Information.

### 2.4 Association Analysis and Meta‐Analysis

Schizophrenia cases used in this study have been described in previous studies [11]. The association between rs1399178 and schizophrenia, and the meta‐analysis, were assessed with PLINK [38].

### 2.5 Dual Luciferase Reporter Gene Assays

We incorporated these target DNA sequences into the regulatory region, i.e., the promoter or enhancer, of the reporter gene vector. Briefly, the DNA fragments (~300–800 bp) containing the different alleles of target SNPs were cloned into pGL3 promoter (RRID: Addgene_212939) or pGL4.11[luc2P] vectors. HEK293T and SH‐SY5Y cells were transfected with the constructed pGL3 promoter or pGL4.11[luc2P] vectors. The pRL‐TK Renilla vector served as the internal control.

### 2.6 Construction of Expression Plasmids, Protein Expression, and Protein Purification

The full‐length *NRF1* gene was PCR amplified from a human cDNA library (reference sequence NCBI: NP_001035199.1, 503 aa). Shorter fragments of *NRF1* were generated using standard PCR‐based methods and confirmed by DNA sequencing. Recombinant proteins were expressed in *Escherichia coli* BL21 (DE3) cells in LB medium at 16°C and purified using a nickel‐NTA agarose (for Trx‐His6‐/GB1‐His6‐/His6‐tagged proteins), followed by size‐exclusion chromatography with a column buffer containing 50 mM Tris pH 8.0, 100 mM NaCl, 1 mM EDTA, and 1 mM DTT. When needed, the tags were cleaved using HRV 3C protease and separated by another step of size‐exclusion chromatography. The protein purities were evaluated by SDS‐PAGE.

### 2.7 Chromatin Immunoprecipitation‐qPCR

ChIP‐qPCR was conducted to assess the binding of TFs NRF1 to the genomic sequence encompassing rs1399178 in SH‐SY5Y cells. The SimpleChIP Enzymatic Chromatin IP Kit (CST, Cat. No: #9003) was used in accordance with manufacturer's instructions. Three immunoprecipitation (IP) groups were established using IgG (CST, Cat. No: 2729, RRID: AB_1031062), H3 (CST, Cat. No: 4620, RRID: AB_1904005), and NRF1 (Abcam, Cat. No: ab175932, RRID: AB_2629496) antibodies. Cross‐linked chromatin fragments (8 μg) were used for the NRF1 antibody as the experimental group, IgG served as the negative control, and H3 as the positive control. DNA purification preceded subsequent qPCR analysis, for which primers were designed to span 100 bp up‐ and down‐stream from rs1399178.

### 2.8 Electrophoretic Mobility Shift Assay

Nuclear extracts of SH‐SY5Y were obtained using the nuclear protein extraction kit (Beyotime, Cat. No: P0028) and quantified using the BCA protein assay kit (Pierce, Cat. No: 23227). Oligonucleotides harboring the G and A alleles of rs1399178 (37 bp) were labeled at their 3′ ends with biotin using the EMSA probe biotin labeling kit (Beyotime, Cat. No: GS008). Single‐strand oligonucleotides were annealed to form double‐stranded complementary sequences. The EMSA procedure was conducted using the Chemiluminescent EMSA Kit (Thermo Scientific, Cat. No: 20148) in accordance with manufacturer's instructions.

### 2.9 Isothermal Titration Calorimetry Assay

Isothermal titration calorimetry (ITC) assay measurements were performed on a Microcal VP‐ITC calorimeter at 25°C. The proteins used for ITC measurements were dissolved in an assay buffer composed of 50 mM Tris pH 8.0, 100 mM NaCl, 1 mM EDTA, and 1 mM DTT. High concentration of DNA (500 μM for RS(G) and RS(A)) was individually loaded into the syringe and titrated into the cell containing low concentration of corresponding interactors (50 μM for DD‐DBD). For each titration point, a 10 μL aliquot of a protein sample in the syringe was injected into the interacting protein in the cell at a time interval of 2–3 min. The ITC titration data were analyzed using the Origin7.0 software and fitted with the two‐site binding model.

### 2.10 Statistical Analysis

Statistical analyses were performed using R software (version 4.3.2). The details of the R packages, functions, and parameter settings used for the analysis are provided in Supporting Information. The Shapiro–Wilk test was applied to assess data normality. Since all data conformed to a normal distribution, Student's t‐test was used to evaluate the significance of differences in luciferase activity between cells transfected with vectors containing different alleles of the target SNP. Statistical significance was defined as *p* < 0.05. Quantitative data are presented as mean ± standard deviation (SD). The exact sample size (n) and the statistical test used are provided in the legend of each figure.

## 3 Results

### 3.1 Functional Genomics Identified 736 Schizophrenia Risk SNPs Disrupting TF Binding

**FIGURE 2:**
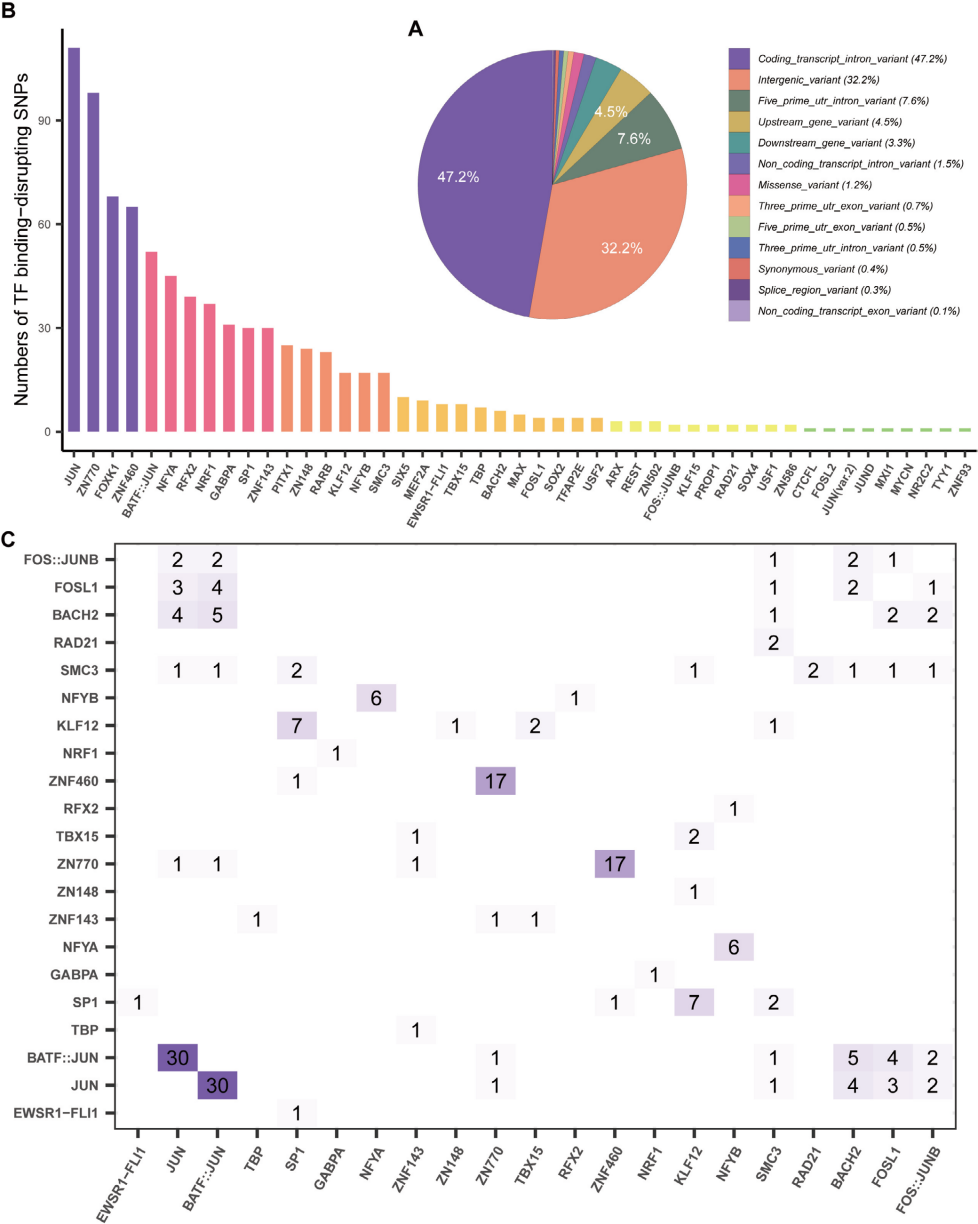
Overview of the TF binding‐disrupting SNPs. The distribution of TF binding‐disrupting SNPs in the human genome. (A) The number of SNPs that affect the binding affinity of each TF. (B) Distribution of the binding‐disrupting SNPs in the human genome. (C) Heatmap showing the number of SNPs that affect binding of two or more TFs.

To identify functional SNPs in the risk loci reported by Li et al., we used a series of functional genomics approaches, as illustrated in Figure 1. Briefly, by integrating ChIP‐seq experimental data and PWM data from public databases, we identified 736 SNPs that affect TF binding whose P‐values were lower than 0.05 after Bonferroni correction (Table S1). The disruptions involve changes that either impair or enhance the binding affinity between SNPs and motifs, leading to a classification as “TF binding‐disrupting SNPs” rather than damaging. Notably, the majority are located in intronic or intergenic regions (717/736), suggesting potential regulatory roles in transcription (Figure 2A). Among the 736 TF binding‐disrupting SNPs, 111 SNPs interfere with JUN binding (13%), 98 with ZN770 binding (12.13%), 68 with FOXK1 (7.69%), and 37 with *NRF1* (7.69%) (Figure 2B). A high number of these SNPs exhibit two or more allelic variations, and the binding affinities with TFs vary based on the specific alleles, with examples represented by rs11014185 and rs10208097. In addition, we observed several SNPs that concurrently disrupted binding to two or more TFs. As shown in Figure 2C, 30 SNPs disrupted the binding of BATF::JUN and JUN, 17 SNPs disrupted the binding of ZNF460 and ZN770, and 7 SNPs disrupted the binding of KLF12 and SPI. Specifically, rs2271319 affected the binding of RAD21 and SMC3. These findings highlight the significance of SNP genetic variation in regulating crucial and intricate molecular interactions within the gene expression regulatory network.

### 3.2 Functional Annotation of TF Binding‐Disrupting SNPs

To identify the most functional SNPs of these TF binding‐disrupting SNPs and elucidate their regulatory mechanisms, we systematically annotated these SNPs by using several bioinformatic databases for subsequent screening. The selection criteria of top functional SNPs for reporter gene assays were as follows: first, the SNP must be significantly associated with gene expression in human brains (*P_FDR* < 0.001); second, the SNP should have a RegulomeDB score of 1. After this, the remaining SNPs were scored based on functional information derived from the HaploReg and 3DSNP databases, specifically considering (i) promoter histone marks, (ii) enhancer histone marks, (iii) DNase protein binding, and (iv) motif changes. The resulting sites were then sorted. Detailed information is found in Supporting Information.

Among the initial set of 736 TF binding‐disrupting SNPs, we observed that 464 SNPs exhibited a significant association with gene expression in human brains in at least one brain eQTL dataset. Notably, 323 identified regulatory SNPs were associated with the expression of the same gene in at least two independent brain eQTL datasets, implying these genes are regulated by these TF binding‐disrupting SNPs. Out of the 464 SNPs with significant eQTL analysis, 335 SNPs have a RegulomeDB score of one. Integrating the information retrieved from HaploReg and 3DSNP, we systematically scored and ranked the remaining SNPs. The top three ranked loci were rs12146541 (G>C, which reduces binding to SMC3 after transition), rs2234053 (G>C, which enhances binding to ZN148 after transition), and rs223333 (C>A, which strengthens binding to RFX2 after transition). Based on the ranking, we randomly selected several of the top 20 SNPs for subsequent reporter gene assays, including rs1399178, rs223333, rs3776130, rs4788211, rs655293, rs7204852, rs12146541, and rs2016875.

### 3.3 Functional Validation of Identified SNPs: rs1399178 Mediates Promoter‐Enhancer Switch

We conducted dual‐luciferase reporter gene assays to validate the impact of the identified functional SNPs in HEK293T cells (Figure S1). With the exception of rs7204852, five SNPs exhibited significant changes in enhancer luciferase activity, indicating significant enhancer regulatory effects. Notably, minor alleles of rs1399178, rs223333, rs3776130, and rs655293 displayed significantly reduced luciferase activity compared to wild‐type variants, whereas the opposite was observed in rs4788211.

We selected four SNPs to evaluate promoter activity, specifically rs1399178, rs2016875, rs655293, and rs12146541, but only rs1399178 and rs12146541 demonstrated altered luciferase activity. Remarkably, a substantial increase in promoter activity was observed following variation of these two SNPs (*p* < 0.001). Collectively, these findings provide evidence for the regulatory function of TF binding‐disrupting SNPs. To delve further into the regulatory mechanisms of the functional SNPs, particular attention was given to SNP rs1399178, which exhibited significant changes in luciferase activity in both enhancer and promoter contexts. At the same time, association analysis showed that rs1399178 was significantly associated with schizophrenia in the European population (*p* = 1.27 × 10^−3^; 33,640 schizophrenia cases and 43,456 controls) and trans‐ancestry meta‐analysis (*p* = 2.92 × 10^−3^; 48,223 schizophrenia cases and 60,717 controls) (Table S2).

**FIGURE 3:**
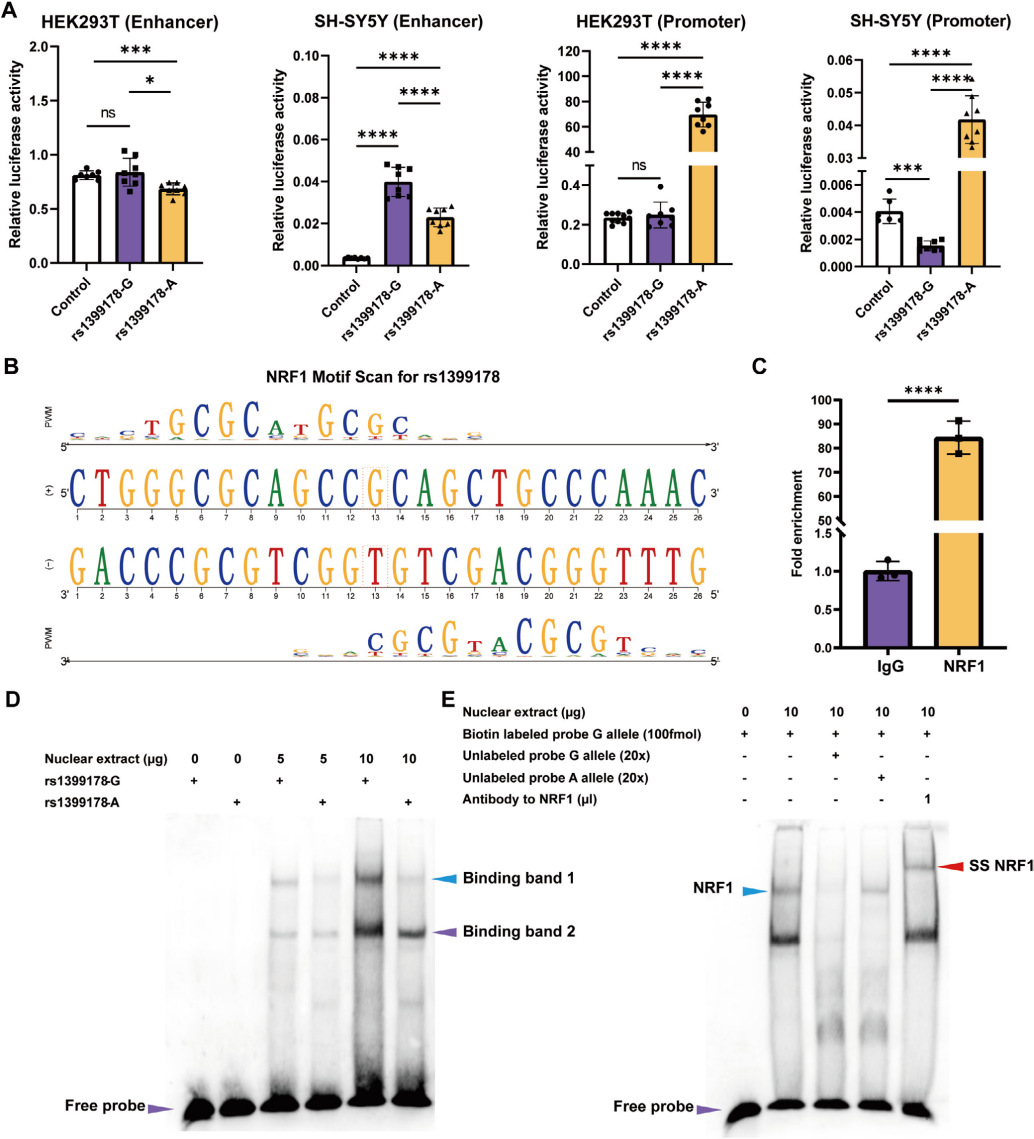
Validation of the regulatory effects of rs1399178. (A) The genomic region containing rs1399178 has enhancer and promoter activities in SH‐SY5Y but not in HEK293T. Compared with the A allele, the G allele exhibits higher enhancer activity and lower promoter activity in HEK293T and SH‐SY5Y (*n* = 8 for each group). (B) rs1399178 is located in the binding motif of NRF1. (C) Significant enrichment of NRF1 on genomic sequence containing rs1399178 (*n* = 3 for each group). (D, E) SNP rs1399178 affects NRF1‐binding affinity. Competitive experiments showing that the G allele had stronger NRF1‐binding affinity compared with the A allele. Super‐shift assay showing that rs1399178 could bind to NRF1 (the purple arrow indicates the free probe, the blue arrow marks the band representing NRF1 binding, and the red arrow denotes the super‐shift band observed in the lane with added NRF1 antibody). *p < 0.05, ***p < 0.001, ****p < 0.0001.

To further confirm the regulatory effects of rs1399178, we conducted reporter gene assays in both HEK293T and SH‐SY5Y cell lines (Figure 3A). Rs1399178 is located in an intergenic region of chromosome 3p22.2, positioned between the LRRFIP2 and GOLGA4. Reporter gene assays revealed that the enhancer luciferase activity of the fragments containing the G allele of rs1399178 was higher than controls in SH‐SY5Y cell lines. The opposite was observed in promoter activity. Interestingly, no significant differences were observed between the G allele and controls in HEK293T, suggesting a cell‐type specific regulatory effect of rs1399178 (i.e., rs1399178 has a regulatory effect in neuroblastoma cell lines but not in HEK293T). Of note, the G allele exhibited significantly higher enhancer activity than the A allele in both tested cell lines. Intriguingly, the A allele conferred significant higher promoter activity compared to the G allele. These results indicate that the genomic sequence containing rs1399178 exhibits both enhancer and promoter activities, with the regulatory effects of rs1399178 on enhancer activity differing from effects on promoter.

Based on the Hi‐C results, we observed that rs1399178 is positioned near the inner boundary of a TAD (Figure S2) that includes interacting genes, such as LRRFIP2, MLH1, and GOLGA4. This suggests that rs1399178 may not only impact the expression of nearby genes but could also influence distant genes within this loop via enhancer–promoter interactions. The findings from the eQTL analysis further support this observation, indicating that the presence of the risk allele is associated with decreased LRRFIP2 and increased MLH1 transcript abundance (Figure S3).

### 3.4 Identification of NRF1 as the Binder for the G Allele of rs1399178

Motif analysis showed that rs1399178 disrupts the binding of the nuclear respiratory factor 1 (*NRF1*), which displays a heightened preference for binding to the DNA sequence containing the G allele of rs1399178 (Figure 3B). ChIP‐qPCR analysis also revealed a significant enrichment of NRF1 binding on the sequence containing rs1399178 compared to the control in the SH‐SH5Y cells (Figure 3C, Figure S4). We performed EMSA to further elucidate the effect of different rs1399178 alleles on the binding affinity of NRF1, which revealed a heightened binding capacity of the G allele compared to the A allele, as evidenced by a more pronounced band (band 1 in Figure 3D). Additionally, the results of the competitive experiment indicated significant disparities in binding affinities between the two alleles. Specifically, the TF in binding band 1 demonstrated a preference for the G allele. Moreover, the super‐shift assays identified NRF1 as the TF in binding band 1, which was detected by electrophoresis upon the introduction of an anti‐NRF1 antibody (indicated by the SS NRF1 arrowhead on the right side of Figure 3E). These observations suggest a preferential binding of NRF1 to the genomic sequence harboring the G allele of rs1399178 in vitro.

### 3.5 Structural Insights Into NRF1 Recognition of DNA

**FIGURE 4:**
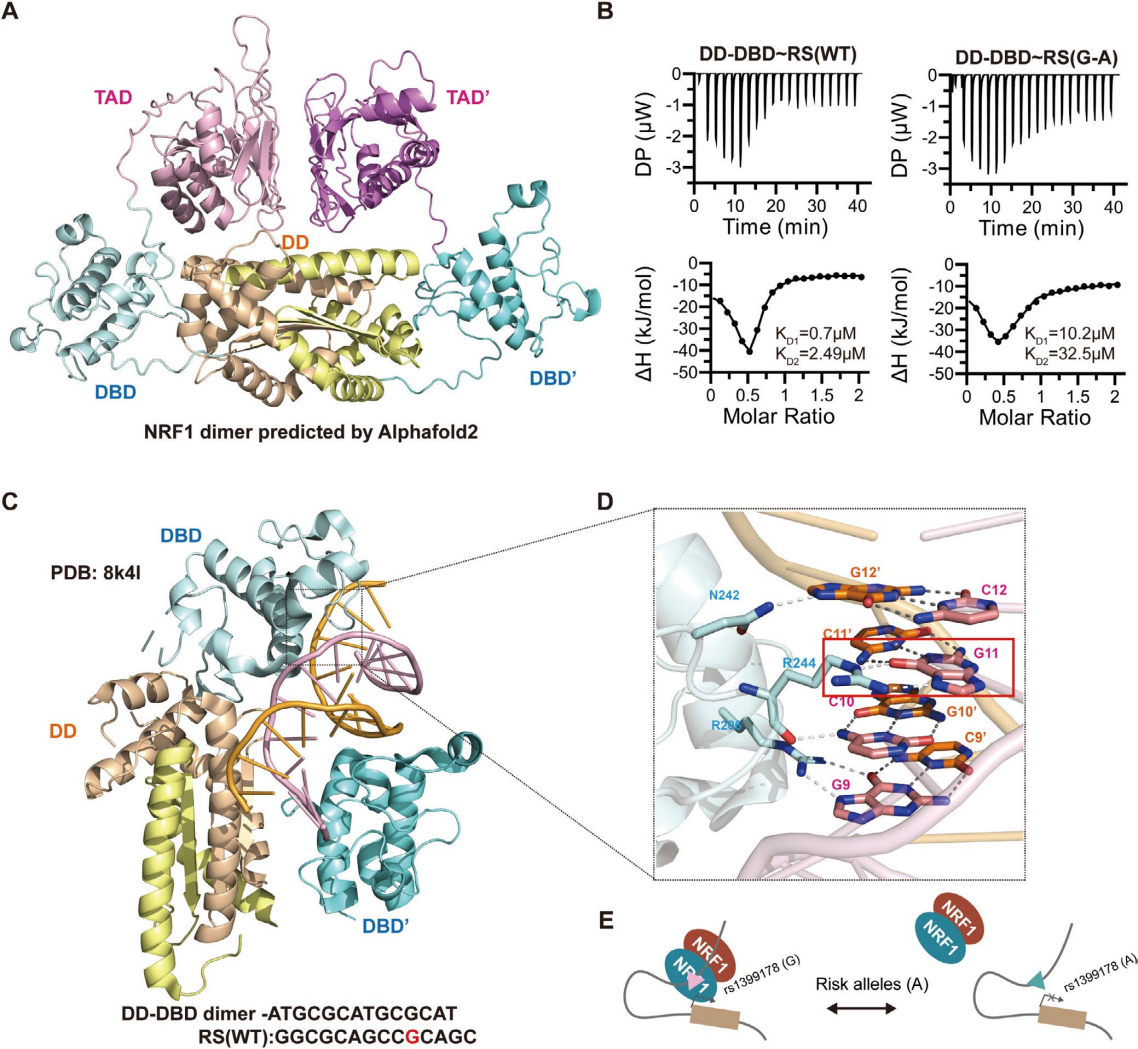
NRF1 comprises a MADS‐box like domain via dimerization. (A) Cartoon diagram depicting the full‐length NRF1 structure. (B) ITC‐based measurements comparing DD‐DBD binding to RS(WT) and RS(G‐A). (C) Cartoon diagram illustrating the binding of DD‐DBD to DNA (PDB ID: 8k4l). (D) Close‐up view of the interactions between DBD and DNA. (E) A schematic diagram illustrating the mechanism by which SNP rs1399178 affects NRF1 binding and downstream gene expression. The G allele of rs1399178 enhances NRF1‐binding affinity, promoting its interaction with the DNA‐binding site. This interaction regulates the transcriptional activity of target genes, potentially influencing downstream biological processes.

To gain further insight into the role of rs1399178 in interfering NRF1‐ DNA recognition, we headed to generate a 3D structure model of full‐length NRF1 by Alphafold2. Sequence alignment revealed that NRF1 is highly conserved among species and belongs to the MADS‐box family (Figures S5 and S6). Alphafold2 prediction suggests that full‐length NRF1 forms a dimer and each monomer comprises three domains: a Dimerization domain (DD, residues 49–177), a DNA‐binding domain (DBD, residues 201–288), and a Transactivation domain (TAD, residues 301–476) (Figure 4A, Figure S7). Traditionally, MADS‐box proteins bind to CArG‐box sequences (CC‐“adenine‐rich”‐GG), though lineage‐specific amino acid variations in the MADS‐box influence DNA‐binding specificity [39, 40]. The NRF1‐responsive element is characterized by a GC‐rich palindrome with the core sequence YGCGCAYGCGCR (Y can be C or T, R can be A or G) [41]. To test the impact of rs1399178, we synthesized two DNA fragments: RS(WT) (5′‐GGCGCAGCCGCAGC‐3′) and RS(G‐A) (5′‐GGCGCAGCCACAGC‐3′) and used ITC to measure the binding affinity of the DD‐DBD complex to these sequences. The G to A mutation at rs1399178 resulted in a tenfold decrease in binding strength, in line with observed biological effects (Figure 4B). We analyzed the complex structure of NRF1 (DD‐DBD) with a consensus DNA sequence ATGCGCATGCGCAT (PDB code:8k4l). The DD‐DBD dimer‐dsDNA structure suggests that DD mediate dimerization of the protein, whereas DBD recognizes short TGCGC DNA sequence [42]. Interface analysis showed that R206 and G9, R244 and C10, G11, N242and G12' form hydrogen bonds for the specific DNA recognition (Figure 4C,D). The mutation from G11 to A in rs1399178 disrupted the hydrogen bond formation between R244 and G11, thereby affecting the stability of the complex.

## 4 Discussion

Following the successful initial GWAS screening of schizophrenia‐related alleles, which identified a single locus around the ZNF804A gene, numerous similar studies have been reported [7]. Each study builds upon the iterative findings of the earlier works, both confirming and expanding upon these discoveries [8, 10, 11, 12, 13, 43, 44]. Presently, over 200 risk loci have been documented, which contributed to the substantial progress in understanding the genetics of schizophrenia, surpassing insights gained in any other field within biological psychiatry [45]. However, formidable challenges remain in interpreting causal variants from these reported risk loci and elucidating their mechanisms of action in the post‐GWAS era. Here, to investigate potential functional regulatory mechanisms of schizophrenia risk loci, we selected 124 risk loci from a GWAS study conducted by Li et al. in 2017 [11]. We leverage several comprehensive datasets and used a multifaceted functional genomics approach to explore the regulatory consequences of these SNPs. Our analysis identified 736 TF binding‐disrupting SNPs that affect TF binding, notably concentrated in intronic and intergenic regions. eQTL analysis suggests that they may influence the expression of multiple genes (Figure S8). Reporter gene assay of randomly selected TF binding‐disrupting SNPs confirmed that the majority of these mutations exhibit regulatory functions. We note that changes of the same SNP to different genotypes may lead to varying binding affinities for the same TF. Furthermore, many SNPs disrupted the binding of two or more TFs. Together, these findings demonstrate the complexity of identifying causal variants and interpreting their regulatory mechanisms based on GWAS‐derived SNP loci, urging some caution in the interpretation. Moreover, our results underscore the significance of integrative approaches combining genomics, functional assays, and structural analysis to unravel the intricate nature of psychiatric disorders, such as schizophrenia.

Regulatory elements are categorized as either promoters or enhancers based on their genomic location and can be associated with histone markers and regulatory functions. Here, we studied in detail a bifunctional regulatory element, rs1399178, which serves as both promoter and enhancer. Reporter gene assays conducted in SH‐SY5Y and HEK293T cell lines demonstrated the bifunctional regulatory effect of rs1399178. The G allele consistently displayed significantly higher enhancer activity and lower promoter activity than the A allele in both cell lines. This observation aligns with the hypothesis that the genomic sequence harboring rs1399178 may mediate a switch between enhancer and promoter activities through the TF. Similarly, Li et al. demonstrated that high‐risk schizophrenia SNPs rs1801311 1exhibit cell line‐specific regulatory activity [46]. Furthermore, leveraging 3D genome data, we ascertained the presence of rs1399178 in an active gene‐regulating TAD, which justifies its classification as a bifunctional element. Our results also suggest the involvement of this variant in long‐range regulatory activities, possibly acting as an enhancer to modulate the expression of other genes. Within the local TAD, factors such as local chromatin architecture and the abundance/occupancy of different TFs may influence dual gene regulation. Based on the eQTL analysis, we found that the risk allele A of rs1399178 is associated with lower expression of *LRRFIP2* and higher expression of *MLH1*. *MLH1* encodes a mismatch repair protein that is essential for maintaining genomic stability. Dysregulation of *MLH1* can lead to genomic instability and abnormal neurodevelopment, thereby increasing the risk of schizophrenia [47]. Using a Bayesian framework to integrate heterogeneous genomic data, *MLH1* has been identified as a top‐ranked risk gene for schizophrenia. Additionally, experimental studies have validated its association with the disorder for the first time. Furthermore, *MLH1* may have genetic overlap with other neuropsychiatric disorders, such as bipolar disorder and depression, highlighting its central role in mental illness. Notably, *LRRFIP2* has also been implicated in other brain and mental disorders, such as Alzheimer's disease [48, 49]. The biological mechanisms involving *LRRFIP2* are evident across multiple pathological contexts, suggesting that this gene may influence schizophrenia risk through diverse regulatory pathways. The mechanisms through which rs1399178 exerts its effects remain unclear, specifically whether it induces changes in loop structures by altering TF‐binding properties. To determine whether this functional SNP was associated with schizophrenia across multiple populations, we conducted a case–control association study. In both European populations and in the trans‐ancestry analyses spanning diverse ethnic groups, rs1399178 exhibits a significant association with schizophrenia. Interestingly, this effect is not observed in the Chinese population, which underscores the importance of stratifying schizophrenia populations for more nuanced research into the genetic basis of this condition [13, 45]. Notably, rs1399178 has a different minor allele frequency (MAF) between Europeans (MAF, 0.431, minor allele: A) and Chinese (MAF, 0.069) populations. Further research is required to evaluate the impact of genetic variation on hierarchical genome structures [50].

Intriguingly, the disruption of NRF1 binding by rs1399178 was clearly observed, with a heightened preference for binding to the G allele. Lower binding affinity of NRF1 was observed at the risk alleles through EMSA and ITC. Recently, Liu elucidated the molecular mechanism by which NRF1 recognizes specific DNA sequences in dimeric form [42]. Our sequences exhibit striking similarity to the consensus binding sequence GCGCATGCGC of NRF1 as enumerated in the article. The results demonstrates that substitution of G11 in the conserved binding sequence GCGCATGCGC with either T or A results in a respective decrease in binding affinity by 4‐ and 6‐fold, consistent with our findings. The diminished binding affinity significantly compromises NRF1's transcriptional activity on downstream genes. NRF1 has been reported to regulate ~471 downstream genes, including mitochondria‐ and neurodevelopment‐related loci [51, 52]. The in vitro binding assay demonstrated that the A allele of rs1399178 significantly weakens the binding strength. The structural insights into NRF1's DNA recognition and dimer formation provide a basis for understanding its role in transcriptional regulation. Through the validation of rs1399178, we have further substantiated the rationale of our analytical framework, establishing that TF binding‐disrupting SNPs have a significant influence on the binding of TFs.

Our study possesses several notable strengths. First, given the highly tissue‐specific nature of genetic regulatory elements, this investigation exclusively incorporates the most recent and comprehensive ChIP‐Seq data from brain tissues or neuronal cell lines. This stringent criterion ensures the sole examination of risk variants located within actively regulated regions of the brain that are characterized by corresponding TF binding. Second, we systematically characterize the regulatory mechanisms of reported schizophrenia risk loci using a relatively high‐throughput approach. By integrating functional genomics and experimental validation, we verified several preciously identified TF‐associated SNPs. Third, our transformation of SNPs into specific target genes through eQTL analysis provides novel insights for future research into the mechanisms of schizophrenia and drug development. Fourth, the use of multiple molecular approaches revealed that rs1399178 is a bifunctional regulatory element located within an active TAD. This suggests that the locus may regulate various genes within the loop through distinct regulatory mechanisms, contributing to a deeper understanding of the mechanisms underlying schizophrenia. Finally, we provided evidence for the molecular mechanism of the binding between rs1399178 and NRF1, further validating the characteristic of NRF1 in recognizing specific DNA sequences and offering new insights into investigating schizophrenia susceptibility loci.

Our study also has several limitations. First, our analysis relied solely on SH‐SY5Y and HEK293T cell lines for reporter gene assays. Given the observed cell‐type‐specific effects, emphasizing the importance of considering tissue‐specific regulatory mechanisms, it is imperative to incorporate additional cell lines relevant to schizophrenia for a more comprehensive investigation in the future. Second, this study exclusively focused on single nucleotide variants, neglecting other types of genetic mutations, such as copy number variations, chromatin structural loci, and rare mutations, all of which are implicated in schizophrenia. Further research is thus required to understand the genetic mechanisms of schizophrenia in relation to these types of variants. Third, we did not experimentally validate the downstream target genes affected by rs1399178.

## 5 Conclusion

We identified 736 SNPs that impact the binding of 47 TFs and validated the regulatory effects of several of these SNPs through reporter gene experiments. We elucidated the bifunctional regulatory element of rs1399178, an inter‐genetic schizophrenia risk variant that mediates promoter‐enhancer switch. We demonstrated that the A allele of rs1399178 attenuates the binding affinity of NRF1, due to specific amino acids of NRF1's MADS‐box domain. Our findings contribute to a more nuanced understanding of the regulatory mechanisms associated with rs1399178 in the context of schizophrenia. The combination of genomics, functional assays, structural analysis, and in‐depth exploration of NRF1's recognition mechanisms provide a comprehensive framework for future research into the genetic mechanisms of schizophrenia and potential therapeutic interventions.

## Author Contributions

G.H. and Q.L. conceived the concept; L.J., B.H., G.H., and Q.L. designed experiments; G.H. led the project with assistance from L.J., B.H., D.R., X.W., L.H., and Q.L.; L.J. performed experiments with assistance from B.H., D.R., L.L., Y.B., F.Y., K.H., K.L., X.L., and T.Y.; L.J., B.H., L.L., Z.L., Y.S., and Q.L. analyzed data; L.J., B.H., Q.L., and G.H. wrote the manuscript with input from all coauthors.

## Conflicts of Interest

The authors declare no conflicts of interest.

## Supporting information


Data S1.

## Data Availability

The data that support the findings of this study are available in GTRD, eQTL Catalogue, RegulomeDB, HaploReg, 3DSNP v2.0 and 4DN data portal. These data were derived from the following resources available in the public domain: GTRD, http://gtrd.biouml.org/; eQTL Catalogue, https://www.ebi.ac.uk/eqtl/; RegulomeDB, https://www.regulomedb.org/; HaploReg, http://www.broadinstitute.org/mammals/haploreg/haploreg.php; 3DSNP v2.0, https://omic.tech/3dsnpv2/; 4DN data portal, htps://data.4dnucleome.org/. Other data can be available from the corresponding author upon reasonable request.
