# Supplementary material for: Identification of Schizophrenia‐Risk Regulatory Variant rs1399178 in the Non‐coding Region With Its Impact on NRF1 Binding
Source: CNS Neurosci Ther. 2025 Feb 28;31(3):e70275. doi: 10.1111/cns.70275 (PMC11868986; doi:10.1111/cns.70275)
Supplement: Supplementary file 1 — Data S1. [file CNS-31-e70275-s001.docx]

**Supplementary Materials**

**Description of Additional Supplementary Tables and Figures**

**Supplementary Tables**

Supplementary Table 1. 736 SNPs that disrupt the binding of TFs from the 23000 SCZ SNPs.

Supplementary Table 2. rs1399178 is significantly associated with schizophrenia.

**Supplementary Figures**

Supplementary Figure 1. Validation of the regulatory effects of the TF binding‑disrupting SNPs with dual luciferase reporter gene assays.

Supplementary Figure 2. rs1399178 is located in a multi loop TAD defined using a Hi-C resolution of 50 kb.

Supplementary Figure 3. Association significance between rs1399178 and its nearby genes in the human brain.

Supplementary Figure 4. Sequence alignment of NRF1.

Supplementary Figure 5. Predicted structure of NRF1 dimer by alphafold2.

Supplementary Figure 6. NRF1 MADS-box Like Domain Dimerization and Structural Analysis.

Supplementary Figure 7. Numbers of Genes disrupted by TF binding-disrupting SNPs.

Supplementary Figure 8. Experimental validation and efficiency analysis of Chip-qPCR Assay.

**Supplementary Table 1. 736 SNPs that disrupt the binding of TFs from the 23000 SCZ SNPs.**

| snpid | Ref | Alt | Chr | Pos | Disrupted TFs | pval_ref | pval_snp | pval_rank | pval_rank_bof |
| --- | --- | --- | --- | --- | --- | --- | --- | --- | --- |
| rs10039233:G | A | G | chr5 | 50351630 | EWSR1-FLI1 | 1.35E-06 | 5.98E-09 | 0 | 0 |
| rs10039233:T | A | T | chr5 | 50351630 | EWSR1-FLI1 | 1.35E-06 | 0.000102 | 0 | 0 |
| rs10043101 | T | G | chr5 | 50386092 | JUN | 9.80E-07 | 0.269402 | 0 | 0 |
| rs10053586 | T | C | chr5 | 140831175 | BATF::JUN | 0.010943 | 6.20E-05 | 0 | 0 |
| rs10070003 | A | C | chr5 | 153145532 | TBP | 0.000578 | 0.382695 | 0 | 0 |
| rs10072421:T | C | T | chr5 | 45548857 | FOXK1 | 0.096041 | 0.000404 | 0 | 0 |
| rs10077234:A | C | A | chr5 | 138578510 | SP1 | 0 | 8.20E-05 | 0 | 0 |
| rs10077234:T | C | T | chr5 | 138578510 | SP1 | 0 | 2.33E-06 | 0 | 0 |
| rs10084932 | A | T | chr4 | 169431550 | BATF::JUN | 0.025851 | 2.73E-05 | 0 | 0 |
| rs10091031:T | A | T | chr8 | 4332658 | GABPA | 0.238585 | 0.000303 | 1.83E-06 | 0.028517 |
| rs10101096 | A | C | chr8 | 38434629 | NFYA | 0.070321 | 0.000249 | 0 | 0 |
| rs10102053 | C | T | chr8 | 59836470 | ZNF143 | 0.000487 | 0.022393 | 9.93E-07 | 0.015503 |
| rs10106136 | A | T | chr8 | 110447798 | JUN | 0.038733 | 3.36E-05 | 0 | 0 |
| rs1013016:A | G | A | chr7 | 105115369 | JUN | 0.019957 | 3.55E-05 | 1.88E-06 | 0.029304 |
| rs1013016:A | G | A | chr7 | 105115369 | BATF::JUN | 0.008554 | 1.54E-05 | 0 | 0 |
| rs10134840 | G | A | chr14 | 84231151 | JUN | 0.019045 | 3.36E-05 | 1.88E-06 | 0.029304 |
| rs10166751 | T | A | chr2 | 200018259 | NFYA | 0.010935 | 7.84E-05 | 0 | 0 |
| rs1019040 | T | C | chr7 | 105132389 | TFAP2E | 0.000255 | 2.64E-06 | 0 | 0 |
| rs10204513 | G | C | chr2 | 27998058 | ZN148 | 0.000377 | 0.329103 | 0 | 0 |
| rs10208097:C | T | C | chr2 | 193142779 | USF2 | 0.000436 | 3.81E-10 | 0 | 0 |
| rs10216482 | T | C | chr8 | 38200843 | ZN770 | 1.19E-06 | 0 | 0 | 0 |
| rs10221847 | T | C | chr2 | 192933504 | SIX5 | 0.023788 | 7.33E-05 | 1.97E-07 | 0.003068 |
| rs1022649 | A | G | chr6 | 111798778 | FOXK1 | 0.000375 | 0.064176 | 0 | 0 |
| rs1030166 | G | A | chr5 | 140805888 | FOXK1 | 0.087617 | 0.000511 | 0 | 0 |
| rs1037710 | C | T | chr2 | 193135265 | JUN | 2.43E-05 | 0.016849 | 0 | 0 |
| rs10454830:G | T | G | chr5 | 50585146 | TBX15 | 0.000105 | 9.37E-07 | 0 | 0 |
| rs10460398 | C | T | chr2 | 199350019 | JUN | 2.43E-05 | 0.344566 | 0 | 0 |
| rs10461329 | G | C | chr4 | 169597376 | ZN770 | 2.84E-06 | 0.510004 | 0 | 0 |
| rs10476933:A | C | A | chr5 | 140536078 | SOX4 | 0 | 0.004554 | 0 | 0 |
| rs10476933:G | C | G | chr5 | 140536078 | SOX4 | 0 | 0.005566 | 0 | 0 |
| rs10503253 | C | A | chr8 | 4323322 | SIX5 | 0.000158 | 0.030027 | 1.97E-07 | 0.003068 |
| rs10515686:T | G | T | chr5 | 153169559 | BATF::JUN | 0.021547 | 3.68E-05 | 0 | 0 |
| rs10515686:T | G | T | chr5 | 153169559 | JUN | 0.101857 | 2.43E-05 | 0 | 0 |
| rs10744560:G | C | G | chr12 | 2277933 | JUN | 0.01502 | 0.000237 | 0 | 0 |
| rs10744560:G | C | G | chr12 | 2277933 | BATF::JUN | 0.010943 | 2.33E-05 | 0 | 0 |
| rs10744560:G | C | G | chr12 | 2277933 | JUN | 0.026651 | 2.56E-05 | 0 | 0 |
| rs10747490 | G | A | chr1 | 97860240 | ZN770 | 0 | 3.01E-06 | 0 | 0 |
| rs10750866 | A | G | chr11 | 57637306 | PITX1 | 0.15655 | 2.42E-06 | 0 | 0 |
| rs10791108 | T | A | chr11 | 130979459 | FOXK1 | 0.000331 | 0.068764 | 0 | 0 |
| rs10867832:C | G | C | chr9 | 82234655 | RFX2 | 0.012483 | 1.17E-06 | 0 | 0 |
| rs10883797 | T | C | chr10 | 102899925 | ZNF460 | 2.64E-05 | 3.68E-07 | 1.14E-06 | 0.017762 |
| rs10933 | C | T | chr3 | 52685800 | SP1 | 9.22E-07 | 0.000431 | 6.81E-07 | 0.010634 |
| rs10940090:G | C | G | chr5 | 50156321 | ZNF143 | 0.022393 | 0.000391 | 9.93E-07 | 0.015503 |
| rs10950407 | T | G | chr7 | 1856030 | ZNF143 | 0.020542 | 0.000223 | 9.93E-07 | 0.015503 |
| rs10950407 | T | G | chr7 | 1856030 | TBX15 | 0.001238 | 7.26E-06 | 0 | 0 |
| rs10950415 | A | G | chr7 | 1874841 | EWSR1-FLI1 | 7.00E-06 | 0.000492 | 5.43E-08 | 0.000847 |
| rs10950425:G | C | G | chr7 | 1887328 | NRF1 | 2.00E-07 | 0.00061 | 4.43E-07 | 0.006913 |
| rs11014185:A | T | A | chr10 | 18425418 | PROP1 | 0.000896 | 0.622818 | 0 | 0 |
| rs11014185:G | T | G | chr10 | 18425418 | PROP1 | 0.000896 | 0.859755 | 0 | 0 |
| rs11057223 | T | C | chr12 | 123270508 | KLF12 | 0.000996 | 7.50E-06 | 1.82E-06 | 0.028406 |
| rs11057223 | T | C | chr12 | 123270508 | TBX15 | 0.000218 | 2.09E-06 | 2.37E-07 | 0.003705 |
| rs11062170 | G | C | chr12 | 2239678 | RFX2 | 0.000563 | 0.802686 | 1.51E-06 | 0.023553 |
| rs11064688 | T | C | chr12 | 110004036 | PITX1 | 2.42E-06 | 0.165614 | 0 | 0 |
| rs11068810 | T | C | chr12 | 109896191 | ZN770 | 3.33E-07 | 0 | 0 | 0 |
| rs11068824 | C | T | chr12 | 109898440 | RARB | 3.19E-05 | 0.011087 | 0 | 0 |
| rs11069045 | T | G | chr12 | 109956331 | ZN148 | 0.329103 | 0.000697 | 0 | 0 |
| rs1108074 | C | T | chr12 | 2224318 | BATF::JUN | 0.011109 | 2.39E-05 | 0 | 0 |
| rs1108074 | C | T | chr12 | 2224318 | JUN | 0.269402 | 1.16E-06 | 0 | 0 |
| rs111249650 | A | T | chr17 | 20008517 | GABPA | 0.238585 | 0.000306 | 2.09E-06 | 0.03268 |
| rs11128855:G | T | G | chr3 | 17767711 | ZNF460 | 0.002168 | 2.40E-05 | 1.14E-06 | 0.017762 |
| rs111324888:G | C | G | chr4 | 169448837 | GABPA | 0.26695 | 0.000787 | 2.09E-06 | 0.03268 |
| rs11136730:T | A | T | chr8 | 4330471 | PITX1 | 0.165614 | 2.42E-06 | 0 | 0 |
| rs111449880 | G | A | chr11 | 46406305 | ZN770 | 3.04E-07 | 0 | 6.27E-07 | 0.009789 |
| rs11153120:G | C | G | chr6 | 108653250 | ZN148 | 0.000299 | 0.329103 | 0 | 0 |
| rs111658964 | T | C | chr5 | 152810765 | ZN770 | 1.15E-06 | 0 | 0 | 0 |
| rs11167615:C | T | C | chr5 | 153188802 | NFYA | 0.01995 | 0.000139 | 0 | 0 |
| rs11191416 | T | G | chr10 | 102845159 | ZNF143 | 0.020542 | 0.000681 | 9.93E-07 | 0.015503 |
| rs11191424 | G | A | chr10 | 102866129 | JUN | 0.572182 | 1.09E-06 | 0 | 0 |
| rs11191426 | G | T | chr10 | 102867473 | ZNF460 | 8.13E-09 | 1.55E-06 | 4.50E-08 | 0.000702 |
| rs11191607:A | G | A | chr10 | 103199431 | ZN770 | 0 | 2.84E-06 | 0 | 0 |
| rs11191607:A | G | A | chr10 | 103199431 | ZNF460 | 2.21E-08 | 3.71E-06 | 4.50E-08 | 0.000702 |
| rs11191607:T | G | T | chr10 | 103199431 | ZN770 | 0 | 5.84E-06 | 0 | 0 |
| rs11191607:T | G | T | chr10 | 103199431 | ZNF460 | 2.21E-08 | 8.66E-06 | 0 | 0 |
| rs11191667 | G | A | chr10 | 103369141 | JUN | 0.422046 | 4.59E-05 | 0 | 0 |
| rs11210146 | A | G | chr1 | 73156324 | FOXK1 | 0.000474 | 0.079985 | 0 | 0 |
| rs11210193 | A | G | chr1 | 73278190 | GABPA | 0.238585 | 0.000892 | 2.09E-06 | 0.03268 |
| rs11210220 | T | G | chr1 | 73383972 | JUN | 4.81E-05 | 0.940977 | 0 | 0 |
| rs11210288 | T | C | chr1 | 73572540 | JUN | 0.305891 | 4.81E-05 | 0 | 0 |
| rs11210292:G | C | G | chr1 | 73587940 | MAX | 0.006349 | 6.06E-06 | 2.25E-06 | 0.035063 |
| rs11210898:G | C | G | chr1 | 43638790 | TBX15 | 0.00015 | 2.09E-06 | 2.37E-07 | 0.003705 |
| rs1121194 | T | C | chr7 | 105340686 | ZN770 | 3.04E-07 | 0 | 6.27E-07 | 0.009789 |
| rs11242436 | A | G | chr5 | 138601629 | BATF::JUN | 0.010943 | 2.73E-05 | 0 | 0 |
| rs11242436 | A | G | chr5 | 138601629 | JUN | 0.01809 | 8.41E-07 | 0 | 0 |
| rs112444116:A | G | A | chr18 | 79884045 | KLF12 | 2.80E-05 | 0.003059 | 1.82E-06 | 0.028406 |
| rs112490454:G | A | G | chr1 | 150419916 | NRF1 | 0.006232 | 7.56E-07 | 3.54E-07 | 0.005519 |
| rs112646892 | C | T | chr1 | 243609000 | ZN770 | 0 | 3.04E-07 | 6.27E-07 | 0.009789 |
| rs112779734:C | T | C | chr22 | 41038194 | ZNF460 | 3.37E-07 | 8.12E-10 | 0 | 0 |
| rs112919142 | G | A | chr6 | 30877149 | ZNF460 | 4.61E-07 | 4.33E-05 | 1.14E-06 | 0.017762 |
| rs113579391:A | C | A | chr1 | 150274698 | RARB | 0.011087 | 3.19E-05 | 0 | 0 |
| rs113627959 | C | A | chr17 | 19902737 | PITX1 | 2.30E-05 | 0.268551 | 0 | 0 |
| rs113833946 | T | C | chr17 | 19955593 | ZNF143 | 0.020542 | 0.000252 | 9.93E-07 | 0.015503 |
| rs113837569:C | G | C | chr12 | 109862839 | RARB | 3.19E-05 | 0.011087 | 0 | 0 |
| rs11533038 | T | C | chr9 | 82241776 | GABPA | 0.252522 | 0.000691 | 2.09E-06 | 0.03268 |
| rs115397636 | C | T | chr6 | 28716355 | NFYA | 0.000128 | 0.01995 | 0 | 0 |
| rs115397636 | C | T | chr6 | 28716355 | NFYB | 7.46E-05 | 0.558594 | 0 | 0 |
| rs11606677 | G | A | chr11 | 57615904 | RFX2 | 1.25E-06 | 0.435187 | 0 | 0 |
| rs11611677:A | C | A | chr12 | 123333016 | ZNF460 | 8.12E-10 | 1.16E-07 | 1.14E-06 | 0.017762 |
| rs11611679:G | C | G | chr12 | 123333023 | ZN770 | 0 | 7.13E-07 | 0 | 0 |
| rs11611679:G | C | G | chr12 | 123333023 | ZNF460 | 8.12E-10 | 5.55E-07 | 0 | 0 |
| rs11611679:T | C | T | chr12 | 123333023 | ZNF460 | 8.12E-10 | 2.77E-07 | 0 | 0 |
| rs11611679:T | C | T | chr12 | 123333023 | ZN770 | 0 | 1.19E-06 | 0 | 0 |
| rs1163082 | T | C | chr10 | 103256350 | ZN770 | 7.13E-07 | 0 | 0 | 0 |
| rs1163086 | A | G | chr10 | 103279822 | JUN | 5.34E-05 | 0.035489 | 0 | 0 |
| rs11633501 | T | C | chr15 | 61539481 | JUN | 0.417621 | 2.43E-05 | 0 | 0 |
| rs11633501 | T | C | chr15 | 61539481 | BATF::JUN | 0.013005 | 3.68E-05 | 0 | 0 |
| rs11636157 | A | C | chr15 | 61550358 | NFYA | 0.078589 | 0.0003 | 0 | 0 |
| rs116375696 | G | A | chr12 | 109880690 | NRF1 | 8.02E-07 | 0.00652 | 3.54E-07 | 0.005519 |
| rs11652894 | C | T | chr17 | 18049125 | RARB | 3.19E-05 | 0.011087 | 0 | 0 |
| rs11654622 | T | C | chr17 | 1350669 | RARB | 3.19E-05 | 0.011087 | 0 | 0 |
| rs11682011 | G | A | chr2 | 28081194 | NRF1 | 1.24E-06 | 0.00652 | 4.43E-07 | 0.006913 |
| rs11693528:G | C | G | chr2 | 199871784 | ZN770 | 0 | 2.84E-06 | 0 | 0 |
| rs11693528:T | C | T | chr2 | 199871784 | ZN770 | 0 | 1.19E-06 | 0 | 0 |
| rs116959331 | A | G | chr22 | 41536770 | ZN770 | 1.02E-06 | 0 | 0 | 0 |
| rs117085262:G | C | G | chr14 | 84277010 | ZN770 | 0 | 1.19E-06 | 0 | 0 |
| rs117085262:T | C | T | chr14 | 84277010 | ZN770 | 0 | 7.13E-07 | 0 | 0 |
| rs11710807 | C | T | chr3 | 37018830 | RARB | 0.011087 | 3.19E-05 | 0 | 0 |
| rs11720064 | G | T | chr3 | 37060028 | PROP1 | 0.534486 | 0.000776 | 0 | 0 |
| rs117220747 | T | C | chr8 | 17156365 | ZN148 | 0.329103 | 0.000108 | 0 | 0 |
| rs11738711 | G | A | chr5 | 50246086 | NFYA | 0.08213 | 0.000217 | 0 | 0 |
| rs11750075 | G | A | chr5 | 50534852 | EWSR1-FLI1 | 0.000492 | 7.00E-06 | 5.43E-08 | 0.000847 |
| rs11759525 | G | A | chr6 | 28866510 | ZNF143 | 0.000252 | 0.020542 | 9.93E-07 | 0.015503 |
| rs11764212:A | C | A | chr7 | 2027958 | MEF2A | 0.159137 | 4.61E-06 | 0 | 0 |
| rs11764212:T | C | T | chr7 | 2027958 | MEF2A | 0.159137 | 1.72E-05 | 0 | 0 |
| rs11774769 | G | A | chr8 | 38180285 | ZNF460 | 7.29E-08 | 1.36E-05 | 4.50E-08 | 0.000702 |
| rs117938174:G | T | G | chr4 | 23409898 | ZN502 | 1.52E-05 | 0.027342 | 2.25E-06 | 0.035166 |
| rs11811400 | A | G | chr1 | 73084304 | NFYA | 0.016821 | 0.000139 | 0 | 0 |
| rs118120999 | G | A | chr1 | 150457964 | ZNF460 | 8.13E-09 | 7.28E-07 | 1.14E-06 | 0.017762 |
| rs11882106 | A | G | chr19 | 30503860 | JUN | 2.43E-05 | 0.143493 | 0 | 0 |
| rs11902254 | A | C | chr2 | 57853962 | BATF::JUN | 0.005389 | 2.66E-05 | 0 | 0 |
| rs11926827 | C | G | chr3 | 37224634 | ZN148 | 0.329103 | 0.000377 | 0 | 0 |
| rs11946332 | C | G | chr4 | 169702831 | PITX1 | 2.30E-05 | 0.268551 | 0 | 0 |
| rs11952137:A | T | A | chr5 | 50204266 | JUN | 2.56E-05 | 0.076424 | 0 | 0 |
| rs11966136 | T | G | chr6 | 28871296 | NRF1 | 0.007514 | 4.67E-07 | 9.35E-09 | 0.000146 |
| rs11988759 | C | T | chr8 | 110592965 | FOXK1 | 0.071129 | 0.000375 | 0 | 0 |
| rs11989357:G | C | G | chr8 | 38337043 | ZN770 | 2.69E-06 | 0 | 0 | 0 |
| rs11996851:G | T | G | chr8 | 142326352 | ZNF93 | 0.000825 | 8.75E-06 | 8.28E-07 | 0.012918 |
| rs12040333:C | G | C | chr1 | 43848369 | TYY1 | 0.019788 | 2.12E-06 | 1.66E-06 | 0.025913 |
| rs12053349:G | C | G | chr2 | 193044700 | ZNF143 | 0.022393 | 0.000487 | 9.93E-07 | 0.015503 |
| rs12136559:G | T | G | chr1 | 73130110 | MAX | 6.06E-06 | 0.00886 | 0 | 0 |
| rs12137388 | C | T | chr1 | 73169630 | FOXK1 | 0.06641 | 0.000292 | 0 | 0 |
| rs12137601 | G | A | chr1 | 73616547 | JUN | 0.657305 | 1.13E-07 | 0 | 0 |
| rs12139217:A | G | A | chr1 | 73086322 | JUN | 0.029334 | 2.71E-05 | 0 | 0 |
| rs12146541:C | G | C | chr11 | 57741206 | SMC3 | 0.000223 | 0.067639 | 4.78E-08 | 0.000746 |
| rs12166627 | T | A | chr22 | 41014911 | PITX1 | 2.42E-06 | 0.15655 | 0 | 0 |
| rs12193701 | A | T | chr6 | 31363840 | BATF::JUN | 0.000159 | 0.03258 | 0 | 0 |
| rs12193701 | A | T | chr6 | 31363840 | JUN | 1.25E-07 | 0.016849 | 0 | 0 |
| rs12196602 | T | A | chr6 | 108601129 | ZN770 | 0 | 1.02E-06 | 0 | 0 |
| rs12196602 | T | A | chr6 | 108601129 | ZNF460 | 5.91E-08 | 6.21E-06 | 1.14E-06 | 0.017762 |
| rs12201301 | G | A | chr6 | 31039780 | BATF::JUN | 3.68E-05 | 0.013284 | 0 | 0 |
| rs12208205 | A | T | chr6 | 31387377 | NFYA | 0.000495 | 0.076269 | 0 | 0 |
| rs12212799 | G | A | chr6 | 30991666 | ZNF460 | 3.68E-07 | 2.40E-05 | 1.14E-06 | 0.017762 |
| rs12214258 | G | T | chr6 | 31052033 | ZN770 | 0 | 1.15E-06 | 0 | 0 |
| rs12214258 | G | T | chr6 | 31052033 | ZNF460 | 8.12E-10 | 9.81E-06 | 0 | 0 |
| rs12219609:C | G | C | chr10 | 103408775 | USF1 | 0.061815 | 1.83E-05 | 6.38E-07 | 0.009953 |
| rs12268583 | C | T | chr10 | 103319405 | ZNF460 | 3.68E-07 | 2.40E-05 | 1.14E-06 | 0.017762 |
| rs12305489 | A | C | chr12 | 110734451 | ZN770 | 3.33E-07 | 0 | 0 | 0 |
| rs12306277:C | T | C | chr12 | 110568377 | ZNF460 | 3.37E-07 | 8.12E-10 | 0 | 0 |
| rs12332385:A | T | A | chr5 | 127878652 | BATF::JUN | 0.025851 | 2.66E-05 | 0 | 0 |
| rs12332385:A | T | A | chr5 | 127878652 | JUN | 0.022934 | 7.21E-07 | 0 | 0 |
| rs12364435 | T | C | chr11 | 24336595 | JUN | 0.357412 | 9.31E-07 | 0 | 0 |
| rs12364435 | T | C | chr11 | 24336595 | BATF::JUN | 0.014534 | 6.20E-05 | 0 | 0 |
| rs12386951 | C | G | chr8 | 38295074 | CTCFL | 0.155735 | 0.000238 | 4.30E-07 | 0.006705 |
| rs12386951 | C | G | chr8 | 38295074 | SP1 | 0.129194 | 5.78E-06 | 7.90E-07 | 0.012325 |
| rs12386951 | C | G | chr8 | 38295074 | ZNF460 | 4.33E-05 | 5.55E-07 | 1.14E-06 | 0.017762 |
| rs12413386:C | T | C | chr10 | 103402563 | ZNF460 | 3.37E-07 | 8.12E-10 | 0 | 0 |
| rs12418827:T | G | T | chr11 | 109519923 | RFX2 | 0.902343 | 0.000971 | 1.51E-06 | 0.023553 |
| rs12438659 | G | A | chr15 | 78532582 | RARB | 3.19E-05 | 0.011087 | 0 | 0 |
| rs12447915 | C | T | chr16 | 30078587 | NFYA | 0.0003 | 0.050948 | 0 | 0 |
| rs12464988 | C | T | chr2 | 27835359 | FOXK1 | 0.079985 | 0.000474 | 0 | 0 |
| rs12485286 | A | C | chr3 | 50378211 | ZNF143 | 0.019221 | 4.10E-05 | 9.93E-07 | 0.015503 |
| rs12486847:A | T | A | chr3 | 52657096 | JUN | 5.62E-05 | 0.068565 | 0 | 0 |
| rs12486847:C | T | C | chr3 | 52657096 | JUN | 5.62E-05 | 0.45326 | 0 | 0 |
| rs12498978:A | T | A | chr4 | 169607321 | BATF::JUN | 2.33E-05 | 0.013005 | 0 | 0 |
| rs12498978:C | T | C | chr4 | 169607321 | BATF::JUN | 2.33E-05 | 0.014534 | 0 | 0 |
| rs12534625 | T | G | chr7 | 137382870 | GABPA | 0.252522 | 0.000582 | 2.09E-06 | 0.03268 |
| rs12550717 | G | A | chr8 | 10409196 | FOXK1 | 0.0586 | 0.000331 | 0 | 0 |
| rs12575721 | T | C | chr11 | 46802131 | GABPA | 0.228968 | 0.000222 | 1.83E-06 | 0.028517 |
| rs12596749 | A | T | chr16 | 4484482 | JUN | 0.064298 | 2.43E-05 | 0 | 0 |
| rs12623056 | G | A | chr2 | 192944950 | JUN | 0.349834 | 5.34E-05 | 0 | 0 |
| rs12624148 | C | T | chr2 | 27733614 | FOXK1 | 0.079985 | 0.000436 | 0 | 0 |
| rs12629699 | T | C | chr3 | 52585776 | ZN770 | 1.19E-06 | 0 | 0 | 0 |
| rs12651909 | C | T | chr5 | 50373396 | NFYB | 5.92E-05 | 0.630255 | 0 | 0 |
| rs12651909 | C | T | chr5 | 50373396 | NFYA | 0.000128 | 0.018451 | 0 | 0 |
| rs12655983 | T | G | chr5 | 45307824 | JUN | 5.25E-08 | 3.36E-05 | 1.88E-06 | 0.029304 |
| rs12655983 | T | G | chr5 | 45307824 | BATF::JUN | 2.39E-05 | 0.014534 | 0 | 0 |
| rs12656748 | C | T | chr5 | 109707528 | EWSR1-FLI1 | 7.00E-06 | 0.000429 | 5.43E-08 | 0.000847 |
| rs12657267 | T | C | chr5 | 152593173 | BATF::JUN | 0.011441 | 2.73E-05 | 0 | 0 |
| rs12657267 | T | C | chr5 | 152593173 | JUN | 0.086625 | 3.36E-05 | 0 | 0 |
| rs1265744 | C | T | chr12 | 110588979 | ZN770 | 0 | 1.19E-06 | 0 | 0 |
| rs12667625 | G | A | chr7 | 105093091 | REST | 2.20E-05 | 0.063741 | 1.45E-06 | 0.022629 |
| rs12667946:C | T | C | chr7 | 1887490 | NRF1 | 0.016255 | 2.49E-06 | 4.43E-07 | 0.006913 |
| rs12679965 | G | A | chr8 | 4330382 | RARB | 3.19E-05 | 0.011087 | 0 | 0 |
| rs12680715 | T | G | chr8 | 59779158 | JUN | 5.62E-05 | 0.086625 | 0 | 0 |
| rs12693677 | T | A | chr2 | 192976014 | RFX2 | 0.682419 | 1.54E-06 | 0 | 0 |
| rs12696125 | A | T | chr3 | 161919527 | JUN | 0.077585 | 5.62E-05 | 0 | 0 |
| rs12697055:T | G | T | chr5 | 50317827 | JUN | 0.021786 | 2.43E-05 | 0 | 0 |
| rs12697055:T | G | T | chr5 | 50317827 | JUN | 0.02011 | 0.000329 | 0 | 0 |
| rs12697055:T | G | T | chr5 | 50317827 | BATF::JUN | 0.049162 | 0.000435 | 0 | 0 |
| rs12705308 | C | T | chr7 | 105364521 | ZNF460 | 8.13E-09 | 2.94E-06 | 0 | 0 |
| rs12898254 | C | A | chr15 | 83901447 | ARX | 0.008637 | 2.66E-05 | 0 | 0 |
| rs12901723:C | G | C | chr15 | 83942878 | ZN148 | 0.329103 | 7.08E-05 | 0 | 0 |
| rs12901723:T | G | T | chr15 | 83942878 | ZN148 | 0.329103 | 0.000276 | 0 | 0 |
| rs12917201:C | G | C | chr15 | 83841662 | EWSR1-FLI1 | 7.00E-06 | 0.000713 | 0 | 0 |
| rs12917201:C | G | C | chr15 | 83841662 | SP1 | 0.003231 | 1.12E-05 | 1.77E-06 | 0.027553 |
| rs12939020 | G | A | chr17 | 18046849 | ZNF460 | 7.29E-08 | 9.81E-06 | 4.50E-08 | 0.000702 |
| rs12963180:A | T | A | chr18 | 79860789 | JUN | 0.014803 | 2.56E-05 | 1.88E-06 | 0.029304 |
| rs12992313 | G | A | chr2 | 199402310 | GABPA | 0.000691 | 0.259455 | 2.09E-06 | 0.03268 |
| rs12992890 | T | C | chr2 | 27837964 | ZN770 | 3.04E-07 | 0 | 6.27E-07 | 0.009789 |
| rs12994036:T | A | T | chr2 | 192958423 | FOXK1 | 0.076615 | 0.000436 | 0 | 0 |
| rs13009957 | G | A | chr2 | 232848527 | ZNF460 | 8.12E-10 | 3.37E-07 | 0 | 0 |
| rs13010276:G | C | G | chr2 | 193096130 | JUN | 0.005663 | 1.09E-06 | 0 | 0 |
| rs13016447:C | T | C | chr2 | 199921131 | ZNF460 | 3.37E-07 | 8.12E-10 | 0 | 0 |
| rs13021208 | C | T | chr2 | 27678861 | NRF1 | 8.05E-07 | 0.564297 | 0 | 0 |
| rs13063160 | T | C | chr3 | 52568258 | JUN | 1.09E-06 | 0.014803 | 0 | 0 |
| rs13065851 | T | A | chr3 | 52610820 | PITX1 | 2.42E-06 | 0.165614 | 0 | 0 |
| rs13076193:A | C | A | chr3 | 52691945 | ZN770 | 0 | 2.84E-06 | 0 | 0 |
| rs13076193:T | C | T | chr3 | 52691945 | ZN770 | 0 | 1.15E-06 | 0 | 0 |
| rs13081031 | C | T | chr3 | 52640526 | ZN770 | 0 | 4.28E-06 | 0 | 0 |
| rs13100015:A | C | A | chr3 | 161985507 | ZNF460 | 8.12E-10 | 1.11E-06 | 0 | 0 |
| rs13100015:G | C | G | chr3 | 161985507 | ZNF460 | 8.12E-10 | 4.61E-07 | 0 | 0 |
| rs13100015:T | C | T | chr3 | 161985507 | ZNF460 | 8.12E-10 | 3.37E-07 | 0 | 0 |
| rs13149311 | C | T | chr4 | 103056543 | NFYA | 0.01995 | 0.000101 | 0 | 0 |
| rs13165162:C | A | C | chr5 | 45790938 | SOX2 | 0.000604 | 0.971603 | 0 | 0 |
| rs13184940 | T | C | chr5 | 140793333 | BATF::JUN | 1.54E-05 | 0.010943 | 0 | 0 |
| rs13186181:G | A | G | chr5 | 50500583 | SP1 | 0.000102 | 5.72E-07 | 2.87E-06 | 0.044749 |
| rs13187428 | T | G | chr5 | 109759844 | JUN | 6.86E-07 | 0.344566 | 0 | 0 |
| rs13187428 | T | G | chr5 | 109759844 | BATF::JUN | 0.000307 | 0.07098 | 0 | 0 |
| rs13237370 | A | T | chr7 | 105183843 | FOXK1 | 0.000436 | 0.096041 | 0 | 0 |
| rs13238384 | T | G | chr7 | 113954228 | FOXK1 | 0.071129 | 0.000375 | 0 | 0 |
| rs13250438 | T | C | chr8 | 110559321 | FOXK1 | 0.000375 | 0.071129 | 0 | 0 |
| rs13258063:G | C | G | chr8 | 8236592 | ZNF143 | 0.024883 | 0.000753 | 9.93E-07 | 0.015503 |
| rs13267290 | A | C | chr8 | 110466092 | FOXK1 | 0.073812 | 0.000404 | 0 | 0 |
| rs1327201 | T | C | chr6 | 111820529 | FOXK1 | 0.000436 | 0.079985 | 0 | 0 |
| rs132911:G | C | G | chr22 | 41408156 | SIX5 | 0.030027 | 0.000158 | 1.97E-07 | 0.003068 |
| rs132922 | G | A | chr22 | 41416080 | ZN770 | 0 | 1.19E-06 | 0 | 0 |
| rs1338831 | A | C | chr6 | 64435782 | JUN | 3.36E-05 | 0.032262 | 0 | 0 |
| rs13426868 | A | G | chr2 | 104399896 | ZNF460 | 1.11E-06 | 8.12E-10 | 0 | 0 |
| rs1349507 | C | T | chr2 | 199217898 | SMC3 | 0.000154 | 0.053498 | 4.78E-08 | 0.000746 |
| rs1349507 | C | T | chr2 | 199217898 | RAD21 | 1.36E-05 | 0.185801 | 1.63E-06 | 0.025409 |
| rs138124594 | G | A | chr3 | 37165024 | RARB | 0.011087 | 3.19E-05 | 0 | 0 |
| rs138377250:A | C | A | chr12 | 123207831 | RFX2 | 1.77E-06 | 0.010172 | 0 | 0 |
| rs138502279:T | G | T | chr16 | 13627064 | ZN770 | 0 | 2.84E-06 | 0 | 0 |
| rs138802037 | G | A | chr8 | 38327425 | BATF::JUN | 0.049162 | 0.00043 | 0 | 0 |
| rs1397236 | G | A | chr3 | 162043290 | FOXK1 | 0.076615 | 0.000375 | 0 | 0 |
| rs1399178 | G | A | chr3 | 37217178 | NRF1 | 4.38E-07 | 0.024623 | 9.35E-09 | 0.000146 |
| rs140851627:C | G | C | chr14 | 29768574 | ZNF143 | 0.024883 | 0.000909 | 9.93E-07 | 0.015503 |
| rs1421174:A | G | A | chr17 | 20046877 | TBX15 | 5.99E-07 | 5.07E-05 | 2.37E-07 | 0.003705 |
| rs1421174:T | G | T | chr17 | 20046877 | TBX15 | 5.99E-07 | 0.000124 | 0 | 0 |
| rs142245917:T | C | T | chr3 | 181345568 | BATF::JUN | 0.053835 | 0.00043 | 0 | 0 |
| rs142494453:A | G | A | chr1 | 97753600 | JUN | 1.25E-07 | 0.014803 | 0 | 0 |
| rs142494453:C | G | C | chr1 | 97753600 | JUN | 1.25E-07 | 0.005974 | 0 | 0 |
| rs143449160 | C | T | chr12 | 110031581 | RARB | 3.19E-05 | 0.011087 | 0 | 0 |
| rs143493854 | G | A | chr16 | 13636950 | BATF::JUN | 0.046388 | 0.000289 | 0 | 0 |
| rs143493854 | G | A | chr16 | 13636950 | JUN | 0.01502 | 0.000116 | 0 | 0 |
| rs143493854 | G | A | chr16 | 13636950 | BACH2 | 0.035887 | 9.62E-07 | 0 | 0 |
| rs144256346:G | C | G | chr11 | 57603939 | RARB | 3.19E-05 | 0.011087 | 0 | 0 |
| rs144256346:T | C | T | chr11 | 57603939 | RARB | 3.19E-05 | 0.011087 | 0 | 0 |
| rs144937153:T | C | T | chr5 | 152916512 | NFYA | 0.08213 | 0.000217 | 0 | 0 |
| rs145237336 | G | A | chr12 | 123271423 | RFX2 | 0.008146 | 8.97E-07 | 0 | 0 |
| rs146910176 | C | T | chr12 | 110784850 | ZNF460 | 8.12E-10 | 3.37E-07 | 0 | 0 |
| rs1472237 | T | C | chr7 | 113985756 | FOXK1 | 0.00059 | 0.102451 | 0 | 0 |
| rs148227879:C | A | C | chr1 | 98161714 | TBP | 0.000875 | 0.612726 | 0 | 0 |
| rs148564912 | G | A | chr1 | 150639840 | ZNF460 | 8.12E-10 | 1.11E-06 | 0 | 0 |
| rs149205384:C | G | C | chr5 | 152918069 | NFYA | 0.0003 | 0.08213 | 0 | 0 |
| rs149205384:T | G | T | chr5 | 152918069 | NFYA | 0.0003 | 0.076269 | 0 | 0 |
| rs1494233 | G | A | chr8 | 110580085 | RFX2 | 0.009705 | 1.33E-06 | 0 | 0 |
| rs150300:G | C | G | chr15 | 89400959 | ZN148 | 0.329103 | 0.000324 | 0 | 0 |
| rs150398891 | G | A | chr2 | 200249011 | ZN770 | 0 | 1.19E-06 | 0 | 0 |
| rs151176288:T | A | T | chr3 | 37258104 | FOXK1 | 0.064176 | 0.000331 | 0 | 0 |
| rs151849:T | A | T | chr5 | 127834951 | JUN | 3.36E-05 | 0.086625 | 0 | 0 |
| rs151856:G | A | G | chr5 | 127886099 | NRF1 | 0.004481 | 5.33E-07 | 3.54E-07 | 0.005519 |
| rs1536057 | C | T | chr6 | 108564420 | NRF1 | 8.02E-07 | 0.932937 | 0 | 0 |
| rs1561245 | C | T | chr2 | 72904038 | FOXK1 | 0.06641 | 0.000375 | 0 | 0 |
| rs1567453 | T | C | chr3 | 162064249 | GABPA | 0.252522 | 0.000582 | 2.09E-06 | 0.03268 |
| rs1568750:T | A | T | chr2 | 224477880 | NFYA | 0.06719 | 0.000217 | 0 | 0 |
| rs1576436 | G | T | chr1 | 73618121 | ZNF143 | 0.000235 | 0.020542 | 9.93E-07 | 0.015503 |
| rs1605978:C | G | C | chr3 | 162095335 | SIX5 | 0.019857 | 3.61E-05 | 1.97E-07 | 0.003068 |
| rs1616484 | A | G | chr12 | 123183405 | JUN | 9.80E-07 | 0.005974 | 0 | 0 |
| rs1630820:G | C | G | chr12 | 123032370 | ZNF460 | 4.61E-07 | 8.12E-10 | 0 | 0 |
| rs1642917:T | A | T | chr2 | 224447992 | ZN770 | 0 | 3.33E-07 | 0 | 0 |
| rs16832256 | C | A | chr3 | 181014717 | RFX2 | 2.01E-06 | 0.024853 | 0 | 0 |
| rs169365:C | G | C | chr22 | 41443710 | ZN148 | 0.329103 | 0.00056 | 0 | 0 |
| rs16966529 | G | A | chr16 | 9865264 | MEF2A | 0.218661 | 3.35E-05 | 1.52E-06 | 0.02375 |
| rs17006365:A | T | A | chr2 | 27900507 | FOXK1 | 0.000404 | 0.089092 | 0 | 0 |
| rs17029770:A | G | A | chr2 | 104370572 | MEF2A | 0.264866 | 4.04E-05 | 0 | 0 |
| rs17029770:T | G | T | chr2 | 104370572 | MEF2A | 0.264866 | 3.40E-05 | 0 | 0 |
| rs17052256 | A | G | chr3 | 52559103 | FOXK1 | 0.000334 | 0.0586 | 0 | 0 |
| rs1718682 | A | G | chr8 | 8281422 | TBX15 | 0.000238 | 3.58E-06 | 2.37E-07 | 0.003705 |
| rs17195098 | C | T | chr3 | 36852474 | FOXK1 | 0.079985 | 0.000474 | 0 | 0 |
| rs17204675 | T | C | chr3 | 37094306 | BATF::JUN | 2.59E-05 | 0.013284 | 0 | 0 |
| rs1740377:T | C | T | chr6 | 111721915 | JUN | 0.037147 | 5.07E-05 | 0 | 0 |
| rs17480230 | G | A | chr2 | 224535147 | JUN | 0.081301 | 2.43E-05 | 0 | 0 |
| rs17729512 | A | G | chr8 | 88293773 | JUN | 2.43E-05 | 0.01809 | 0 | 0 |
| rs17787804 | C | T | chr11 | 46485335 | JUN | 0.029334 | 3.94E-05 | 0 | 0 |
| rs17794609 | C | G | chr16 | 13697608 | JUN | 2.43E-05 | 0.022934 | 0 | 0 |
| rs17794609 | C | G | chr16 | 13697608 | BATF::JUN | 3.77E-05 | 0.025851 | 0 | 0 |
| rs1801368 | C | T | chr7 | 1936821 | NRF1 | 2.27E-07 | 0.33356 | 0 | 0 |
| rs1801368 | C | T | chr7 | 1936821 | NRF1 | 2.13E-06 | 0.000237 | 2.12E-06 | 0.033056 |
| rs1805584 | T | C | chr3 | 180914242 | RFX2 | 1.54E-06 | 0.011807 | 0 | 0 |
| rs1822339 | A | G | chr11 | 24337816 | NFYA | 0.000181 | 0.070321 | 0 | 0 |
| rs1837017:C | T | C | chr16 | 64264254 | KLF12 | 0.001919 | 1.57E-05 | 1.82E-06 | 0.028406 |
| rs1837017:C | T | C | chr16 | 64264254 | SP1 | 0.001187 | 5.50E-06 | 1.53E-06 | 0.02395 |
| rs185666755:A | C | A | chr5 | 90816568 | KLF15 | 1.20E-08 | 0.0003 | 1.95E-06 | 0.030493 |
| rs185666755:G | C | G | chr5 | 90816568 | KLF15 | 1.20E-08 | 0.0003 | 1.95E-06 | 0.030493 |
| rs187461 | T | C | chr5 | 127707692 | FOXK1 | 0.000355 | 0.064176 | 0 | 0 |
| rs1876633 | A | G | chr5 | 90939550 | FOXK1 | 0.000511 | 0.083532 | 0 | 0 |
| rs1879202 | C | T | chr8 | 88285677 | ZN148 | 1.92E-05 | 0.01119 | 0 | 0 |
| rs1885250 | G | A | chr1 | 73400107 | JUN | 0.677987 | 5.62E-05 | 0 | 0 |
| rs1922957:A | C | A | chr6 | 64289475 | NFYB | 0.000124 | 0.662254 | 5.73E-07 | 0.008936 |
| rs1922961:C | G | C | chr6 | 64256945 | NFYA | 0.000233 | 0.070321 | 0 | 0 |
| rs1922961:T | G | T | chr6 | 64256945 | NFYA | 0.000233 | 0.061024 | 0 | 0 |
| rs1922961:T | G | T | chr6 | 64256945 | NFYB | 0.000109 | 0.562897 | 5.73E-07 | 0.008936 |
| rs193172700 | A | G | chr2 | 58132922 | JUN | 0.077585 | 5.62E-05 | 0 | 0 |
| rs1968048 | C | G | chr12 | 110469768 | ZN770 | 0 | 1.72E-06 | 0 | 0 |
| rs1971397:G | C | G | chr3 | 136808458 | ZN770 | 0 | 0.104522 | 0 | 0 |
| rs1971397:T | C | T | chr3 | 136808458 | ZN770 | 0 | 1.15E-06 | 0 | 0 |
| rs1972544:G | A | G | chr5 | 140779372 | PITX1 | 0.15655 | 2.42E-06 | 0 | 0 |
| rs1973512:T | A | T | chr1 | 43662376 | ZN770 | 0 | 3.33E-07 | 0 | 0 |
| rs1979002 | G | T | chr2 | 192974203 | NFYB | 0.000257 | 0.936246 | 5.73E-07 | 0.008936 |
| rs1983631:A | C | A | chr22 | 41248070 | NRF1 | 7.56E-07 | 0.008839 | 9.35E-09 | 0.000146 |
| rs1983631:A | C | A | chr22 | 41248070 | NRF1 | 1.11E-05 | 0.001243 | 2.12E-06 | 0.033056 |
| rs1983631:T | C | T | chr22 | 41248070 | NRF1 | 7.56E-07 | 0.005459 | 4.43E-07 | 0.006913 |
| rs1984658 | G | A | chr12 | 122998879 | ZN770 | 0 | 1.72E-06 | 0 | 0 |
| rs1988093:C | G | C | chr5 | 90734625 | RFX2 | 1.54E-06 | 0.014138 | 0 | 0 |
| rs1988093:T | G | T | chr5 | 90734625 | RFX2 | 1.54E-06 | 0.010172 | 0 | 0 |
| rs199951952:T | A | T | chr5 | 152920682 | NFYB | 0.662254 | 0.000102 | 0 | 0 |
| rs2001338 | A | G | chr8 | 10393707 | NRF1 | 0.006232 | 1.12E-06 | 4.43E-07 | 0.006913 |
| rs2010476 | C | G | chr16 | 64339990 | ZN770 | 0 | 1.72E-06 | 0 | 0 |
| rs201091693 | G | T | chr6 | 64446989 | ZN148 | 6.76E-06 | 0.003016 | 0 | 0 |
| rs201237786 | T | C | chr3 | 136226861 | JUN | 3.94E-05 | 0.029334 | 0 | 0 |
| rs2016875:A | C | A | chr8 | 38401517 | RFX2 | 3.57E-09 | 0.136711 | 0 | 0 |
| rs2016875:T | C | T | chr8 | 38401517 | RFX2 | 3.57E-09 | 0.123692 | 0 | 0 |
| rs202636 | T | C | chr22 | 41456475 | ZNF143 | 0.020542 | 0.00038 | 9.93E-07 | 0.015503 |
| rs202650:C | G | C | chr22 | 41441537 | GABPA | 0.076591 | 6.52E-05 | 1.83E-06 | 0.028517 |
| rs203451:C | A | C | chr17 | 19919583 | ZN770 | 1.19E-06 | 0 | 0 | 0 |
| rs2071203 | C | T | chr3 | 50274469 | SMC3 | 7.63E-05 | 0.062119 | 4.78E-08 | 0.000746 |
| rs2078422 | T | C | chr6 | 28508720 | ZN770 | 0 | 3.33E-07 | 0 | 0 |
| rs2090793:C | G | C | chr2 | 57794393 | GABPA | 0.228968 | 0.000197 | 1.83E-06 | 0.028517 |
| rs2092564 | G | C | chr22 | 41304026 | PITX1 | 2.42E-06 | 0.165614 | 0 | 0 |
| rs2101653:G | A | G | chr3 | 161935881 | FOXK1 | 0.000436 | 0.079985 | 0 | 0 |
| rs2101653:T | A | T | chr3 | 161935881 | FOXK1 | 0.000436 | 0.081644 | 0 | 0 |
| rs2102508:C | A | C | chr3 | 162046460 | RFX2 | 1.54E-06 | 0.012483 | 0 | 0 |
| rs2102949 | G | A | chr12 | 123192216 | ZN770 | 0 | 1.19E-06 | 0 | 0 |
| rs2110194 | C | T | chr3 | 37055596 | FOXK1 | 0.143525 | 0.000759 | 0 | 0 |
| rs2131433:A | C | A | chr17 | 1394253 | ARX | 0.020603 | 0.000219 | 0 | 0 |
| rs2134094 | C | T | chr1 | 73613145 | NFYA | 0.000217 | 0.03686 | 0 | 0 |
| rs2136912 | T | G | chr8 | 59765206 | ZN770 | 0 | 7.13E-07 | 0 | 0 |
| rs2140486:G | C | G | chr2 | 199825379 | SP1 | 1.07E-05 | 0.030901 | 1.46E-06 | 0.022853 |
| rs2140486:G | C | G | chr2 | 199825379 | KLF12 | 7.50E-06 | 0.000996 | 1.82E-06 | 0.028406 |
| rs2140486:G | C | G | chr2 | 199825379 | SP1 | 9.22E-07 | 0.000195 | 2.87E-06 | 0.044749 |
| rs2140486:T | C | T | chr2 | 199825379 | KLF12 | 7.50E-06 | 0.000996 | 1.82E-06 | 0.028406 |
| rs2148197:A | T | A | chr10 | 103132218 | FOXK1 | 0.098598 | 0.000551 | 0 | 0 |
| rs2175385 | T | A | chr8 | 59776456 | FOXK1 | 0.000404 | 0.073812 | 0 | 0 |
| rs2175881 | C | T | chr3 | 37159627 | ZN770 | 0 | 1.19E-06 | 0 | 0 |
| rs2192932:T | A | T | chr7 | 105012818 | NFYA | 0.064223 | 0.000282 | 0 | 0 |
| rs2202974 | A | G | chr3 | 162086230 | FOXK1 | 0.000511 | 0.083532 | 0 | 0 |
| rs2208335 | G | A | chr6 | 83585701 | JUN | 0.037147 | 2.43E-05 | 0 | 0 |
| rs2209590 | A | G | chr10 | 18428753 | NFYB | 0.630255 | 0.000109 | 5.73E-07 | 0.008936 |
| rs223333 | C | A | chr4 | 102868653 | RFX2 | 0.682419 | 1.33E-06 | 0 | 0 |
| rs2234053:C | G | C | chr22 | 41381311 | ZN148 | 0.000257 | 4.97E-07 | 0 | 0 |
| rs2236657 | C | A | chr11 | 134034290 | FOXK1 | 0.073812 | 0.000449 | 0 | 0 |
| rs2236950:A | C | A | chr3 | 50383123 | SMC3 | 0.000329 | 0.083057 | 4.78E-08 | 0.000746 |
| rs2236950:T | C | T | chr3 | 50383123 | SMC3 | 0.000329 | 0.083057 | 4.78E-08 | 0.000746 |
| rs2240696 | A | G | chr5 | 140788485 | NRF1 | 8.02E-07 | 0.001443 | 9.96E-07 | 0.015537 |
| rs2240915 | T | A | chr3 | 52825510 | ZN770 | 3.33E-07 | 0 | 0 | 0 |
| rs2241823 | C | A | chr3 | 63979417 | SMC3 | 1.60E-05 | 0.053498 | 0 | 0 |
| rs2269201:A | G | A | chr5 | 109716958 | KLF12 | 2.80E-05 | 0.003059 | 1.82E-06 | 0.028406 |
| rs2269201:C | G | C | chr5 | 109716958 | KLF12 | 2.80E-05 | 0.003059 | 1.82E-06 | 0.028406 |
| rs2269432:G | T | G | chr3 | 50296800 | ZNF143 | 0.011568 | 0.000184 | 9.93E-07 | 0.015503 |
| rs2271319:A | C | A | chr12 | 109906482 | RAD21 | 0.005993 | 2.33E-06 | 2.22E-06 | 0.034647 |
| rs2271319:A | C | A | chr12 | 109906482 | SMC3 | 0.051986 | 7.47E-06 | 0 | 0 |
| rs2271319:G | C | G | chr12 | 109906482 | SMC3 | 0.051986 | 3.17E-05 | 4.78E-08 | 0.000746 |
| rs2271319:T | C | T | chr12 | 109906482 | SMC3 | 0.051986 | 7.89E-05 | 4.78E-08 | 0.000746 |
| rs2282755:C | G | C | chr3 | 50405369 | SP1 | 0 | 1.16E-06 | 0 | 0 |
| rs2282755:T | G | T | chr3 | 50405369 | SP1 | 0 | 1.81E-06 | 0 | 0 |
| rs2284901:C | A | C | chr14 | 32816317 | ZNF460 | 1.93E-06 | 8.13E-09 | 0 | 0 |
| rs2289250 | C | A | chr3 | 52648930 | JUN | 8.41E-07 | 0.005663 | 0 | 0 |
| rs2293051:C | G | C | chr12 | 117280881 | ZN148 | 0.000521 | 0.329103 | 0 | 0 |
| rs2337631 | C | A | chr12 | 123248777 | SP1 | 5.59E-05 | 0.015874 | 1.48E-06 | 0.023039 |
| rs2337954 | G | A | chr5 | 45651767 | NFYA | 0.000139 | 0.016821 | 0 | 0 |
| rs2357464 | A | G | chr2 | 192979313 | BATF::JUN | 0.011441 | 6.62E-05 | 0 | 0 |
| rs2404546 | C | G | chr16 | 4429733 | ZNF460 | 3.42E-08 | 2.38E-06 | 1.14E-06 | 0.017762 |
| rs2404546 | C | G | chr16 | 4429733 | ZN770 | 0 | 3.33E-07 | 0 | 0 |
| rs2409033 | T | C | chr5 | 127898882 | FOXK1 | 0.000375 | 0.079985 | 0 | 0 |
| rs2413646 | C | T | chr22 | 41510321 | USF2 | 3.21E-10 | 0.000351 | 0 | 0 |
| rs2430484 | G | T | chr7 | 104941515 | NFYA | 0.000249 | 0.078589 | 0 | 0 |
| rs2438361:T | G | T | chr5 | 90794620 | RFX2 | 0.712082 | 0.000893 | 1.51E-06 | 0.023553 |
| rs245156 | G | A | chr5 | 127909451 | FOXK1 | 0.071129 | 0.000377 | 0 | 0 |
| rs245174:G | A | G | chr5 | 127888309 | ZNF460 | 3.37E-07 | 8.12E-10 | 0 | 0 |
| rs245198 | G | T | chr5 | 127842216 | JUN | 3.94E-05 | 0.020939 | 1.88E-06 | 0.029304 |
| rs2460164 | A | T | chr5 | 90762373 | JUN | 0.019957 | 1.23E-06 | 0 | 0 |
| rs2460164 | A | T | chr5 | 90762373 | BATF::JUN | 0.014534 | 2.81E-05 | 0 | 0 |
| rs246041 | C | G | chr5 | 140951543 | ZNF143 | 0.000516 | 0.017255 | 9.93E-07 | 0.015503 |
| rs246042 | G | A | chr5 | 140951382 | JUN | 0.417621 | 2.43E-05 | 0 | 0 |
| rs246056 | G | A | chr5 | 140946015 | NRF1 | 9.36E-06 | 0.264239 | 9.35E-09 | 0.000146 |
| rs2482508:T | C | T | chr10 | 102799204 | PITX1 | 0.268551 | 2.30E-05 | 0 | 0 |
| rs251353:G | C | G | chr5 | 140848579 | SP1 | 0.000574 | 9.22E-07 | 6.81E-07 | 0.010634 |
| rs251356:G | T | G | chr5 | 140849941 | NRF1 | 0.003782 | 7.15E-07 | 4.43E-07 | 0.006913 |
| rs2530225:A | G | A | chr5 | 140722954 | FOXK1 | 0.079985 | 0.000375 | 0 | 0 |
| rs2530240:A | T | A | chr5 | 140680962 | NFYA | 0.000282 | 0.06719 | 0 | 0 |
| rs256017:C | G | C | chr5 | 138564350 | ZN770 | 1.19E-06 | 0 | 0 | 0 |
| rs2569147:T | G | T | chr5 | 127761244 | JUN | 0.103951 | 5.07E-05 | 0 | 0 |
| rs2569160:G | T | G | chr5 | 140649189 | FOXK1 | 0.073812 | 0.000404 | 0 | 0 |
| rs260749 | G | A | chr2 | 199251833 | FOXK1 | 0.079985 | 0.000436 | 0 | 0 |
| rs2609417 | C | A | chr5 | 50150088 | NFYA | 0.000282 | 0.073772 | 0 | 0 |
| rs2616578 | G | T | chr3 | 2427426 | RFX2 | 0.004152 | 6.05E-07 | 0 | 0 |
| rs267692 | G | T | chr1 | 29996615 | NFYA | 0.134735 | 0.0003 | 0 | 0 |
| rs2678891 | T | G | chr2 | 57939508 | JUN | 5.07E-05 | 0.051744 | 0 | 0 |
| rs2683619:T | C | T | chr2 | 57805624 | ARX | 0.002142 | 4.88E-06 | 0 | 0 |
| rs2710331 | T | C | chr3 | 52803839 | NFYB | 0.000139 | 0.440143 | 1.15E-06 | 0.017872 |
| rs2710331 | T | C | chr3 | 52803839 | NFYA | 0.000203 | 0.073772 | 0 | 0 |
| rs2714070:C | A | C | chr11 | 123508679 | NFYA | 0.054482 | 0.000209 | 0 | 0 |
| rs2717003 | A | G | chr2 | 57916303 | ZN770 | 4.28E-06 | 0 | 0 | 0 |
| rs2717040:C | T | C | chr2 | 57942359 | JUN | 2.43E-05 | 0.305891 | 0 | 0 |
| rs2717040:G | T | G | chr2 | 57942359 | JUN | 2.43E-05 | 0.160706 | 0 | 0 |
| rs2764766 | C | G | chr5 | 127877933 | SMC3 | 0.091755 | 0.000494 | 4.78E-08 | 0.000746 |
| rs27683:T | A | T | chr5 | 50865708 | SOX2 | 0.306983 | 2.09E-05 | 0 | 0 |
| rs2784735:G | C | G | chr1 | 8361032 | ZNF143 | 0.022393 | 0.000576 | 9.93E-07 | 0.015503 |
| rs2788117 | T | C | chr9 | 82174174 | JUN | 9.31E-07 | 0.101857 | 0 | 0 |
| rs281777:C | T | C | chr2 | 199974890 | ZN770 | 0 | 3.33E-07 | 0 | 0 |
| rs281777:G | T | G | chr2 | 199974890 | ZN770 | 0 | 2.69E-06 | 0 | 0 |
| rs283487 | A | G | chr2 | 232779301 | FOXK1 | 0.000404 | 0.076615 | 0 | 0 |
| rs28362949 | G | T | chr11 | 57604438 | JUN | 0.029334 | 5.34E-05 | 1.88E-06 | 0.029304 |
| rs28460372:G | T | G | chr8 | 38342703 | PITX1 | 0.165614 | 2.42E-06 | 0 | 0 |
| rs284844 | A | G | chr10 | 102794772 | ZNF143 | 0.020542 | 0.000993 | 9.93E-07 | 0.015503 |
| rs28509440:G | A | G | chr12 | 123401953 | FOSL1 | 6.80E-05 | 0.007328 | 0 | 0 |
| rs28509440:G | A | G | chr12 | 123401953 | BATF::JUN | 3.77E-05 | 0.026834 | 0 | 0 |
| rs28509440:G | A | G | chr12 | 123401953 | BACH2 | 7.09E-06 | 0.080963 | 1.06E-06 | 0.016573 |
| rs28509440:T | A | T | chr12 | 123401953 | BACH2 | 7.09E-06 | 0.077818 | 1.06E-06 | 0.016573 |
| rs28509440:T | A | T | chr12 | 123401953 | BATF::JUN | 3.77E-05 | 0.027923 | 0 | 0 |
| rs28509440:T | A | T | chr12 | 123401953 | FOSL1 | 6.80E-05 | 0.007328 | 0 | 0 |
| rs28516750:A | G | A | chr12 | 123383447 | PITX1 | 2.42E-06 | 0.15655 | 0 | 0 |
| rs28555361 | C | G | chr17 | 18030712 | RFX2 | 0.698121 | 1.77E-06 | 0 | 0 |
| rs28567541 | T | C | chr17 | 20051789 | BATF::JUN | 6.02E-05 | 0.036865 | 0 | 0 |
| rs28567541 | T | C | chr17 | 20051789 | JUN | 0.000183 | 0.01502 | 0 | 0 |
| rs28574664 | C | T | chr12 | 123401837 | ZN770 | 0 | 3.33E-07 | 0 | 0 |
| rs28574664 | C | T | chr12 | 123401837 | ZNF460 | 2.13E-07 | 2.40E-05 | 1.14E-06 | 0.017762 |
| rs2859354:A | G | A | chr6 | 28491467 | GABPA | 0.000609 | 0.252522 | 2.09E-06 | 0.03268 |
| rs2859354:T | G | T | chr6 | 28491467 | GABPA | 0.000609 | 0.252522 | 2.09E-06 | 0.03268 |
| rs2866416 | G | C | chr4 | 102959554 | FOXK1 | 0.073812 | 0.000404 | 0 | 0 |
| rs28888731:A | T | A | chr12 | 123401187 | RFX2 | 1.62E-08 | 0.001883 | 0 | 0 |
| rs2910027 | C | T | chr5 | 153151031 | NFYA | 0.000139 | 0.02458 | 0 | 0 |
| rs2910034:C | G | C | chr5 | 153162881 | NR2C2 | 2.82E-07 | 0.000224 | 0 | 0 |
| rs2921705 | G | A | chr2 | 232927855 | SMC3 | 0.000494 | 0.094928 | 4.78E-08 | 0.000746 |
| rs2926136:C | T | C | chr16 | 64243713 | RFX2 | 0.002661 | 6.22E-09 | 0 | 0 |
| rs2926656 | C | T | chr11 | 57697917 | SMC3 | 0.000117 | 0.053498 | 4.78E-08 | 0.000746 |
| rs2948293:T | C | T | chr8 | 8234198 | FOXK1 | 0.076615 | 0.000436 | 0 | 0 |
| rs295126:A | C | A | chr2 | 200362129 | PITX1 | 2.42E-06 | 0.165614 | 0 | 0 |
| rs295126:G | C | G | chr2 | 200362129 | PITX1 | 2.42E-06 | 0.165614 | 0 | 0 |
| rs2953328:A | C | A | chr1 | 243860378 | MEF2A | 4.04E-05 | 0.250051 | 1.52E-06 | 0.02375 |
| rs2955352 | T | C | chr17 | 18058075 | ZN770 | 1.72E-06 | 0 | 0 | 0 |
| rs2955353:C | T | C | chr17 | 18045665 | TFAP2E | 0.003525 | 4.15E-05 | 0 | 0 |
| rs2955584 | A | G | chr8 | 8234652 | FOXK1 | 0.000375 | 0.068764 | 0 | 0 |
| rs2962809 | A | G | chr5 | 153148011 | JUN | 0.379427 | 5.07E-05 | 0 | 0 |
| rs2962809 | A | G | chr5 | 153148011 | BATF::JUN | 0.011441 | 2.73E-05 | 0 | 0 |
| rs2962809 | A | G | chr5 | 153148011 | FOSL1 | 0.152631 | 7.00E-05 | 0 | 0 |
| rs2967186 | G | A | chr16 | 64268575 | RFX2 | 1.54E-06 | 0.4837 | 0 | 0 |
| rs2974946:T | C | T | chr7 | 113873557 | JUN | 8.41E-07 | 0.101857 | 0 | 0 |
| rs3006936 | A | G | chr1 | 243511470 | REST | 6.36E-05 | 0.12897 | 1.45E-06 | 0.022629 |
| rs302321 | C | A | chr12 | 29775455 | NFYA | 0.034807 | 0.000282 | 0 | 0 |
| rs3109469 | C | T | chr3 | 181175789 | FOXK1 | 0.083532 | 0.000511 | 0 | 0 |
| rs3117338 | G | A | chr6 | 29243732 | TBP | 0.375422 | 0.000578 | 0 | 0 |
| rs3131103:G | C | G | chr6 | 28925757 | RFX2 | 0.014138 | 1.54E-06 | 0 | 0 |
| rs3132580 | G | A | chr6 | 30952347 | FOXK1 | 0.076615 | 0.000436 | 0 | 0 |
| rs3134787 | T | G | chr6 | 31392976 | ZN770 | 1.02E-06 | 0 | 0 | 0 |
| rs314283:G | T | G | chr6 | 104991461 | ZN148 | 0.329103 | 0.00042 | 0 | 0 |
| rs34180096:C | T | C | chr7 | 104968145 | ZNF460 | 3.37E-07 | 8.12E-10 | 0 | 0 |
| rs34181670 | T | C | chr2 | 27796989 | BATF::JUN | 6.62E-05 | 0.039359 | 0 | 0 |
| rs34315079 | A | C | chr3 | 180893179 | ZNF460 | 1.11E-06 | 8.13E-09 | 4.50E-08 | 0.000702 |
| rs34385996 | G | T | chr8 | 110613385 | NFYB | 0.000144 | 0.244269 | 1.15E-06 | 0.017872 |
| rs34438032:A | G | A | chr3 | 136618187 | NFYA | 0.050948 | 0.000128 | 0 | 0 |
| rs34558707:A | C | A | chr7 | 104991083 | ZNF460 | 3.37E-07 | 3.53E-05 | 1.14E-06 | 0.017762 |
| rs34558707:T | C | T | chr7 | 104991083 | ZNF460 | 3.37E-07 | 2.40E-05 | 1.14E-06 | 0.017762 |
| rs34601168 | A | G | chr5 | 50330561 | GABPA | 0.252522 | 0.000609 | 2.09E-06 | 0.03268 |
| rs34779679:C | A | C | chr7 | 114038462 | JUN | 1.09E-06 | 0.005974 | 0 | 0 |
| rs34779679:G | A | G | chr7 | 114038462 | JUN | 1.09E-06 | 0.014803 | 0 | 0 |
| rs34860170 | G | A | chr5 | 154307513 | ZNF460 | 8.12E-10 | 3.37E-07 | 0 | 0 |
| rs34872913 | C | T | chr8 | 110566501 | MAX | 1.46E-05 | 0.012639 | 0 | 0 |
| rs34872913 | C | T | chr8 | 110566501 | MAX | 0.000153 | 0.938116 | 0 | 0 |
| rs34901547 | T | G | chr6 | 64193497 | NFYA | 0.050948 | 0.000172 | 0 | 0 |
| rs34921234 | A | C | chr15 | 84549423 | NFYB | 0.244269 | 0.000124 | 1.15E-06 | 0.017872 |
| rs34966409:A | C | A | chr3 | 2447205 | ZNF143 | 0.000716 | 0.024883 | 9.93E-07 | 0.015503 |
| rs34966409:G | C | G | chr3 | 2447205 | ZNF143 | 0.000716 | 0.024883 | 9.93E-07 | 0.015503 |
| rs34966409:T | C | T | chr3 | 2447205 | ZNF143 | 0.000716 | 0.024883 | 9.93E-07 | 0.015503 |
| rs35072719:A | G | A | chr7 | 111583418 | SP1 | 0 | 1.16E-06 | 0 | 0 |
| rs35072719:C | G | C | chr7 | 111583418 | KLF12 | 7.50E-06 | 0.000915 | 1.82E-06 | 0.028406 |
| rs35072719:C | G | C | chr7 | 111583418 | SP1 | 0 | 1.74E-05 | 0 | 0 |
| rs35072719:T | G | T | chr7 | 111583418 | SP1 | 0 | 2.79E-05 | 0 | 0 |
| rs35072719:T | G | T | chr7 | 111583418 | KLF12 | 7.50E-06 | 0.000915 | 1.82E-06 | 0.028406 |
| rs35159785 | G | A | chr15 | 84135262 | BATF::JUN | 6.20E-05 | 0.010943 | 0 | 0 |
| rs35207602 | G | C | chr6 | 64196733 | SIX5 | 4.39E-05 | 0.021001 | 1.97E-07 | 0.003068 |
| rs35328743:A | C | A | chr6 | 30455996 | JUN | 0.01502 | 0.000116 | 0 | 0 |
| rs35328743:A | C | A | chr6 | 30455996 | FOSL1 | 0.132617 | 6.00E-05 | 0 | 0 |
| rs35328743:A | C | A | chr6 | 30455996 | BATF::JUN | 0.064275 | 0.000289 | 0 | 0 |
| rs35365548 | T | G | chr6 | 30455568 | ZNF143 | 0.000681 | 0.020542 | 9.93E-07 | 0.015503 |
| rs35530507:C | A | C | chr8 | 110534071 | SP1 | 0.013487 | 5.78E-06 | 2.36E-06 | 0.036844 |
| rs35541918 | T | C | chr5 | 50251831 | JUN | 0.016849 | 2.56E-05 | 0 | 0 |
| rs35604463 | G | A | chr14 | 99245695 | ZN148 | 5.71E-05 | 0.0553 | 0 | 0 |
| rs35678296 | A | T | chr3 | 181236330 | JUN | 0.032262 | 5.62E-05 | 1.88E-06 | 0.029304 |
| rs35739573:A | G | A | chr2 | 192939794 | JUN | 0.971942 | 5.62E-05 | 0 | 0 |
| rs35739573:A | G | A | chr2 | 192939794 | BATF::JUN | 0.046388 | 8.94E-05 | 0 | 0 |
| rs35757884 | G | T | chr7 | 105380087 | RFX2 | 1.54E-06 | 0.013622 | 0 | 0 |
| rs35797074 | A | G | chr3 | 162065913 | MEF2A | 4.61E-06 | 0.264866 | 0 | 0 |
| rs35797753:A | G | A | chr7 | 1915278 | GABPA | 1.08E-05 | 0.203453 | 1.47E-06 | 0.023017 |
| rs35797753:C | G | C | chr7 | 1915278 | GABPA | 1.08E-05 | 0.203453 | 1.47E-06 | 0.023017 |
| rs35977430 | C | T | chr17 | 1407156 | BACH2 | 9.62E-07 | 0.002175 | 1.06E-06 | 0.016573 |
| rs35977430 | C | T | chr17 | 1407156 | JUN | 0.000116 | 0.01502 | 0 | 0 |
| rs35993922:A | C | A | chr7 | 105392555 | RFX2 | 1.54E-06 | 0.491654 | 0 | 0 |
| rs35993922:G | C | G | chr7 | 105392555 | RFX2 | 1.54E-06 | 0.467567 | 0 | 0 |
| rs370234933:G | C | G | chr3 | 50312941 | GABPA | 0.000541 | 0.252522 | 2.09E-06 | 0.03268 |
| rs370234933:T | C | T | chr3 | 50312941 | GABPA | 0.000541 | 0.252522 | 2.09E-06 | 0.03268 |
| rs370412981 | A | C | chr2 | 58175242 | NFYA | 0.08213 | 0.000447 | 0 | 0 |
| rs3732385 | C | A | chr3 | 36830702 | NFYB | 5.92E-05 | 0.662254 | 0 | 0 |
| rs3732385 | C | A | chr3 | 36830702 | NFYA | 0.000119 | 0.01995 | 0 | 0 |
| rs3740974 | A | C | chr11 | 46375773 | MAX | 0.005529 | 1.20E-06 | 0 | 0 |
| rs3740974 | A | C | chr11 | 46375773 | MXI1 | 0.00848 | 6.92E-06 | 2.29E-06 | 0.035741 |
| rs3753174:A | G | A | chr5 | 109772236 | RFX2 | 0.008146 | 8.97E-07 | 0 | 0 |
| rs376852830:T | G | T | chr8 | 8281382 | EWSR1-FLI1 | 5.98E-09 | 4.59E-06 | 0 | 0 |
| rs3774323 | T | C | chr3 | 37092960 | ZNF460 | 8.13E-09 | 1.55E-06 | 4.50E-08 | 0.000702 |
| rs3774354 | G | A | chr3 | 52783659 | FOXK1 | 0.073812 | 0.000404 | 0 | 0 |
| rs37746:G | T | G | chr7 | 111278451 | NFYB | 0.936246 | 0.000546 | 1.15E-06 | 0.017872 |
| rs37751 | C | T | chr7 | 111281668 | FOXK1 | 0.073812 | 0.000404 | 0 | 0 |
| rs37759 | C | T | chr7 | 111294626 | BATF::JUN | 2.39E-05 | 0.003966 | 0 | 0 |
| rs37759 | C | T | chr7 | 111294626 | JUN | 3.94E-05 | 0.020939 | 1.88E-06 | 0.029304 |
| rs3776130 | G | T | chr5 | 140692664 | ZN770 | 0 | 1.02E-06 | 0 | 0 |
| rs3778977 | C | T | chr7 | 2120111 | KLF12 | 2.13E-05 | 0.002357 | 1.82E-06 | 0.028406 |
| rs3786800 | T | C | chr19 | 30499793 | JUN | 2.56E-05 | 0.022934 | 0 | 0 |
| rs3789037 | A | C | chr16 | 4427005 | ZNF460 | 0.000159 | 2.38E-06 | 1.14E-06 | 0.017762 |
| rs3791135 | A | G | chr1 | 43583496 | NRF1 | 0.005964 | 8.02E-07 | 4.23E-07 | 0.006607 |
| rs3800909:G | C | G | chr7 | 2119959 | ZNF143 | 3.86E-05 | 0.017255 | 9.93E-07 | 0.015503 |
| rs3803763 | G | C | chr17 | 17793217 | ZNF143 | 8.65E-05 | 0.020542 | 9.93E-07 | 0.015503 |
| rs3806842 | C | T | chr5 | 140833091 | BATF::JUN | 0.027923 | 6.62E-05 | 0 | 0 |
| rs3823752:T | C | T | chr7 | 104984179 | FOXK1 | 0.131088 | 0.000633 | 0 | 0 |
| rs3845343 | C | T | chr1 | 73331623 | RARB | 3.19E-05 | 0.011087 | 0 | 0 |
| rs3933088 | T | C | chr14 | 99238994 | NRF1 | 0.932286 | 3.47E-07 | 0 | 0 |
| rs3935505:G | T | G | chr17 | 17978706 | NFYA | 9.25E-05 | 0.019386 | 0 | 0 |
| rs4021230 | C | G | chr18 | 79798228 | KLF12 | 0.000764 | 7.50E-06 | 1.82E-06 | 0.028406 |
| rs4021230 | C | G | chr18 | 79798228 | SP1 | 2.79E-05 | 0 | 0 | 0 |
| rs4074990 | C | G | chr1 | 73256777 | ZNF460 | 8.13E-09 | 1.24E-06 | 4.50E-08 | 0.000702 |
| rs4144797 | T | C | chr2 | 232697487 | NRF1 | 0.007514 | 7.56E-07 | 9.35E-09 | 0.000146 |
| rs4148863:G | A | G | chr12 | 123084828 | ZNF143 | 0.022393 | 0.000671 | 9.93E-07 | 0.015503 |
| rs4235024 | T | G | chr4 | 169590400 | SMC3 | 0.091755 | 0.000494 | 4.78E-08 | 0.000746 |
| rs4236273:C | G | C | chr7 | 1856705 | SP1 | 0.002652 | 4.34E-06 | 6.81E-07 | 0.010634 |
| rs4236273:C | G | C | chr7 | 1856705 | KLF12 | 0.000915 | 7.50E-06 | 1.82E-06 | 0.028406 |
| rs4242617:G | A | G | chr9 | 82287514 | NFYA | 0.050948 | 0.0003 | 0 | 0 |
| rs4319147 | G | A | chr8 | 10425148 | ZNF143 | 0.000414 | 0.022393 | 9.93E-07 | 0.015503 |
| rs4336110:G | C | G | chr3 | 52590217 | SIX5 | 0.030027 | 0.000158 | 1.97E-07 | 0.003068 |
| rs4372623 | T | C | chr14 | 84285894 | FOXK1 | 0.000375 | 0.068764 | 0 | 0 |
| rs4379243 | G | C | chr5 | 50149933 | FOXK1 | 0.064176 | 0.000375 | 0 | 0 |
| rs4383359 | A | G | chr2 | 72906954 | TBX15 | 3.89E-05 | 5.99E-07 | 2.37E-07 | 0.003705 |
| rs4383359 | A | G | chr2 | 72906954 | KLF12 | 0.001336 | 7.50E-06 | 0 | 0 |
| rs4385208:C | T | C | chr5 | 50309007 | GABPA | 0.252522 | 0.000541 | 2.09E-06 | 0.03268 |
| rs4402589 | T | G | chr16 | 29943333 | ZN770 | 3.33E-07 | 0 | 0 | 0 |
| rs4411554 | C | G | chr17 | 1416471 | RFX2 | 0.000322 | 0.964295 | 1.51E-06 | 0.023553 |
| rs4424915 | G | A | chr16 | 4468391 | ZN770 | 0 | 3.04E-07 | 6.27E-07 | 0.009789 |
| rs4425649:C | A | C | chr7 | 1832742 | ZN770 | 7.13E-07 | 0 | 0 | 0 |
| rs4425649:G | A | G | chr7 | 1832742 | ZN770 | 7.13E-07 | 0 | 0 | 0 |
| rs4459917 | G | T | chr3 | 162112364 | TBP | 0.297643 | 7.06E-05 | 0 | 0 |
| rs4464523 | G | A | chr4 | 169593715 | NRF1 | 8.05E-07 | 0.234346 | 0 | 0 |
| rs4479308:C | G | C | chr17 | 19994132 | ZN770 | 2.84E-06 | 0 | 0 | 0 |
| rs4510624 | T | C | chr5 | 153180168 | NFYA | 0.000217 | 0.08213 | 0 | 0 |
| rs4584446:G | A | G | chr1 | 98023922 | GABPA | 0.238585 | 0.000306 | 2.09E-06 | 0.03268 |
| rs4593807:A | G | A | chr1 | 243841090 | FOXK1 | 0.079985 | 0.000436 | 0 | 0 |
| rs4602407 | A | G | chr3 | 162022550 | JUN | 2.43E-05 | 0.130368 | 0 | 0 |
| rs4603346:C | A | C | chr12 | 91871760 | ZN148 | 0.009084 | 2.11E-05 | 0 | 0 |
| rs4621692 | T | C | chr7 | 1832739 | ZN770 | 7.13E-07 | 0 | 0 | 0 |
| rs4631252 | G | T | chr6 | 64430948 | TBP | 0.049879 | 8.58E-05 | 0 | 0 |
| rs4640182 | A | C | chr16 | 4421290 | ZNF460 | 1.55E-06 | 8.13E-09 | 4.50E-08 | 0.000702 |
| rs4687645 | G | A | chr3 | 52712903 | TBP | 0 | 3.89E-06 | 0 | 0 |
| rs4687645 | G | A | chr3 | 52712903 | ZNF143 | 0.000583 | 0.022393 | 9.93E-07 | 0.015503 |
| rs4687684 | C | T | chr3 | 52884497 | ZN770 | 0 | 1.19E-06 | 0 | 0 |
| rs4688683 | C | T | chr3 | 50202031 | ZNF143 | 0.00079 | 0.020542 | 9.93E-07 | 0.015503 |
| rs4688725 | T | G | chr3 | 50340745 | ZNF143 | 8.65E-05 | 0.020542 | 9.93E-07 | 0.015503 |
| rs4692725:G | A | G | chr4 | 169425103 | ZN770 | 7.13E-07 | 0 | 0 | 0 |
| rs4699044 | A | G | chr4 | 102976939 | GABPA | 0.222657 | 7.45E-05 | 1.83E-06 | 0.028517 |
| rs4719311 | C | T | chr7 | 1842177 | BATF::JUN | 0.049162 | 0.000181 | 0 | 0 |
| rs4721282 | A | G | chr7 | 1985405 | NRF1 | 0.004481 | 5.33E-07 | 3.54E-07 | 0.005519 |
| rs4721302:C | A | C | chr7 | 2010219 | KLF12 | 0.003151 | 2.80E-05 | 1.82E-06 | 0.028406 |
| rs4727623:C | T | C | chr7 | 105403992 | MEF2A | 4.04E-05 | 0.443108 | 0 | 0 |
| rs4730610 | C | T | chr7 | 113957574 | ZNF143 | 0.000993 | 0.024883 | 9.93E-07 | 0.015503 |
| rs4748471 | T | C | chr10 | 18479466 | GABPA | 0.252522 | 0.000691 | 2.09E-06 | 0.03268 |
| rs4759408 | C | A | chr12 | 123231226 | ZNF460 | 1.11E-06 | 9.10E-05 | 1.14E-06 | 0.017762 |
| rs4779042 | A | G | chr15 | 82669792 | RARB | 3.19E-05 | 0.011087 | 0 | 0 |
| rs4788197 | G | A | chr16 | 29959513 | GABPA | 2.41E-05 | 0.006996 | 2.09E-06 | 0.03268 |
| rs4788203:A | G | A | chr16 | 29967506 | ZN770 | 0 | 1.72E-06 | 0 | 0 |
| rs4788203:C | G | C | chr16 | 29967506 | ZN770 | 0 | 3.01E-06 | 0 | 0 |
| rs4788211 | C | T | chr16 | 30004244 | SMC3 | 0.000427 | 0.091755 | 4.78E-08 | 0.000746 |
| rs4788211 | C | T | chr16 | 30004244 | SP1 | 1.02E-05 | 0.008431 | 2.43E-06 | 0.037888 |
| rs4788220 | A | G | chr16 | 30052459 | ZN770 | 1.72E-06 | 0 | 0 | 0 |
| rs4788220 | A | G | chr16 | 30052459 | ZNF460 | 1.36E-05 | 7.29E-08 | 4.50E-08 | 0.000702 |
| rs479573 | C | T | chr11 | 57663562 | BATF::JUN | 2.66E-05 | 0.007093 | 0 | 0 |
| rs4805574 | A | G | chr19 | 30522854 | FOXK1 | 0.000436 | 0.096041 | 0 | 0 |
| rs4820383 | C | A | chr22 | 39565281 | JUN | 0.02011 | 0.000313 | 0 | 0 |
| rs4820383 | C | A | chr22 | 39565281 | BATF::JUN | 0.023369 | 2.59E-05 | 0 | 0 |
| rs4835688 | C | T | chr5 | 138538314 | ZNF460 | 8.12E-10 | 3.37E-07 | 0 | 0 |
| rs4856752 | A | C | chr3 | 162069072 | ZNF460 | 5.29E-05 | 3.37E-07 | 4.50E-08 | 0.000702 |
| rs4856752 | A | C | chr3 | 162069072 | ZN770 | 5.84E-06 | 0 | 0 | 0 |
| rs4875336 | G | C | chr8 | 4335006 | RFX2 | 2.01E-06 | 0.012483 | 0 | 0 |
| rs4875337 | A | T | chr8 | 4342837 | GABPA | 2.62E-05 | 0.211145 | 1.83E-06 | 0.028517 |
| rs4919680:A | T | A | chr10 | 102810361 | EWSR1-FLI1 | 1.11E-13 | 8.32E-07 | 0 | 0 |
| rs4919680:C | T | C | chr10 | 102810361 | EWSR1-FLI1 | 1.11E-13 | 4.59E-06 | 0 | 0 |
| rs4925072 | A | G | chr17 | 20053586 | JUN | 2.43E-05 | 0.269402 | 0 | 0 |
| rs4925136 | G | C | chr17 | 18049688 | RFX2 | 0.001752 | 1.36E-08 | 0 | 0 |
| rs4976993 | C | A | chr8 | 142288019 | NFYA | 0.01995 | 0.000119 | 0 | 0 |
| rs5029110:C | T | C | chr4 | 169392503 | ZNF460 | 3.37E-07 | 8.12E-10 | 0 | 0 |
| rs516921 | T | G | chr1 | 43616091 | SP1 | 0.016508 | 5.79E-06 | 1.12E-06 | 0.017529 |
| rs519357:G | A | G | chr1 | 43649351 | ZN770 | 1.02E-06 | 0 | 0 | 0 |
| rs519669:A | G | A | chr1 | 43609834 | NRF1 | 8.05E-07 | 0.001443 | 1.49E-06 | 0.023302 |
| rs519669:C | G | C | chr1 | 43609834 | NRF1 | 8.05E-07 | 0.009453 | 9.35E-09 | 0.000146 |
| rs547563:A | T | A | chr5 | 50230332 | JUN | 5.62E-05 | 0.077585 | 0 | 0 |
| rs547563:C | T | C | chr5 | 50230332 | JUN | 5.62E-05 | 0.086625 | 0 | 0 |
| rs55661562:C | A | C | chr11 | 46606253 | MEF2A | 4.04E-05 | 0.363868 | 0 | 0 |
| rs55697959 | A | G | chr8 | 4315869 | JUN | 4.81E-05 | 0.400163 | 0 | 0 |
| rs55727014 | A | G | chr5 | 153216617 | NFYA | 0.000119 | 0.03686 | 0 | 0 |
| rs55896955:A | C | A | chr11 | 46660667 | ZNF460 | 3.68E-07 | 2.40E-05 | 1.14E-06 | 0.017762 |
| rs55980974:G | A | G | chr2 | 199360952 | JUND | 0.028417 | 3.43E-06 | 0 | 0 |
| rs55980974:G | A | G | chr2 | 199360952 | FOSL1 | 0.0071 | 1.14E-06 | 0 | 0 |
| rs55980974:G | A | G | chr2 | 199360952 | SMC3 | 0.067639 | 0.00011 | 4.78E-08 | 0.000746 |
| rs55980974:G | A | G | chr2 | 199360952 | FOSL1 | 0.147313 | 2.29E-05 | 0 | 0 |
| rs55980974:G | A | G | chr2 | 199360952 | FOSL2 | 0.053029 | 3.82E-05 | 0 | 0 |
| rs55980974:G | A | G | chr2 | 199360952 | JUN(var.2) | 0.151545 | 2.07E-05 | 0 | 0 |
| rs55980974:G | A | G | chr2 | 199360952 | FOS::JUNB | 0.006744 | 2.36E-05 | 0 | 0 |
| rs55980974:G | A | G | chr2 | 199360952 | JUN | 0.00999 | 5.33E-05 | 0 | 0 |
| rs55980974:G | A | G | chr2 | 199360952 | BACH2 | 0.132192 | 2.60E-05 | 1.06E-06 | 0.016573 |
| rs55980974:G | A | G | chr2 | 199360952 | BATF::JUN | 0.008953 | 2.66E-05 | 0 | 0 |
| rs56000256:T | A | T | chr6 | 64404419 | NRF1 | 7.15E-07 | 0.001443 | 8.80E-07 | 0.013736 |
| rs56033132:G | T | G | chr2 | 224628577 | NFYA | 0.000139 | 0.018451 | 0 | 0 |
| rs56077333:T | C | T | chr15 | 78606661 | ZN770 | 3.33E-07 | 0 | 0 | 0 |
| rs56201009 | A | G | chr1 | 43633075 | NRF1 | 0.000142 | 6.75E-07 | 1.95E-06 | 0.030488 |
| rs56201009 | A | G | chr1 | 43633075 | NRF1 | 0.264239 | 4.63E-08 | 0 | 0 |
| rs56382011 | G | C | chr2 | 200174820 | NRF1 | 4.83E-06 | 0.008839 | 9.96E-07 | 0.015537 |
| rs564641:A | C | A | chr5 | 50227125 | TBP | 0.240425 | 7.06E-05 | 0 | 0 |
| rs56778427 | G | A | chr5 | 50460864 | ZN770 | 2.84E-06 | 0 | 0 | 0 |
| rs568128728:G | C | G | chr11 | 46617548 | KLF12 | 0.000996 | 7.50E-06 | 1.82E-06 | 0.028406 |
| rs56909494 | G | T | chr1 | 150396403 | SOX2 | 0.980491 | 4.70E-05 | 0 | 0 |
| rs56917743:T | A | T | chr4 | 169696003 | NFYB | 6.24E-05 | 0.481453 | 0 | 0 |
| rs570180043:A | C | A | chr2 | 232879423 | ZNF143 | 0.000438 | 0.022393 | 9.93E-07 | 0.015503 |
| rs570180043:G | C | G | chr2 | 232879423 | ZNF143 | 0.000438 | 0.022393 | 9.93E-07 | 0.015503 |
| rs570180043:T | C | T | chr2 | 232879423 | ZNF143 | 0.000438 | 0.022393 | 9.93E-07 | 0.015503 |
| rs57163995 | G | A | chr1 | 150348146 | ZNF460 | 3.68E-07 | 2.40E-05 | 1.14E-06 | 0.017762 |
| rs57163995 | G | A | chr1 | 150348146 | ZN770 | 0 | 3.33E-07 | 0 | 0 |
| rs5758335:T | G | T | chr22 | 41405457 | SP1 | 0.000147 | 0.409796 | 1.12E-06 | 0.017529 |
| rs576390306 | T | C | chr3 | 36824972 | NRF1 | 0.005704 | 4.12E-07 | 9.35E-09 | 0.000146 |
| rs57673732:A | C | A | chr17 | 20036246 | ZN770 | 0 | 2.84E-06 | 0 | 0 |
| rs57673732:A | C | A | chr17 | 20036246 | ZNF460 | 8.13E-09 | 1.11E-06 | 4.50E-08 | 0.000702 |
| rs57673732:T | C | T | chr17 | 20036246 | ZNF460 | 8.13E-09 | 8.62E-07 | 1.14E-06 | 0.017762 |
| rs57673732:T | C | T | chr17 | 20036246 | ZN770 | 0 | 7.13E-07 | 0 | 0 |
| rs57728924:G | A | G | chr17 | 17972028 | NRF1 | 0.007205 | 5.33E-07 | 9.35E-09 | 0.000146 |
| rs57918891:A | G | A | chr8 | 38190619 | JUN | 0.038733 | 2.43E-05 | 0 | 0 |
| rs58003798:C | A | C | chr12 | 109897650 | SP1 | 0.434001 | 0.000563 | 2.43E-06 | 0.037888 |
| rs58263761:A | T | A | chr1 | 8791870 | NFYA | 0.08213 | 0.000249 | 0 | 0 |
| rs58537074 | C | T | chr1 | 150609177 | ZN770 | 0 | 1.02E-06 | 0 | 0 |
| rs58537074 | C | T | chr1 | 150609177 | ZNF460 | 8.12E-10 | 2.77E-07 | 0 | 0 |
| rs585650:A | T | A | chr6 | 64385210 | JUN | 2.33E-05 | 0.025392 | 0 | 0 |
| rs585650:C | T | C | chr6 | 64385210 | JUN | 2.33E-05 | 0.101857 | 0 | 0 |
| rs58693636 | C | T | chr3 | 37061132 | GABPA | 0.000306 | 0.238585 | 2.09E-06 | 0.03268 |
| rs587278 | A | G | chr6 | 64388875 | BATF::JUN | 0.00043 | 0 | 0 | 0 |
| rs587278 | A | G | chr6 | 64388875 | JUN | 2.43E-05 | 4.30E-09 | 0 | 0 |
| rs59278403:G | A | G | chr12 | 109934844 | ZN770 | 1.02E-06 | 0 | 0 | 0 |
| rs595741:A | C | A | chr11 | 124811487 | JUN | 4.81E-05 | 0.038733 | 0 | 0 |
| rs59721702:C | G | C | chr3 | 32021957 | NFYB | 0.310708 | 0.000144 | 1.15E-06 | 0.017872 |
| rs59721702:C | G | C | chr3 | 32021957 | RFX2 | 1.77E-06 | 0.014138 | 0 | 0 |
| rs59746564 | T | C | chr15 | 82564407 | ZNF460 | 8.12E-10 | 1.11E-06 | 0 | 0 |
| rs59978327:T | G | T | chr8 | 38326077 | ZNF460 | 2.94E-06 | 0.000532 | 4.50E-08 | 0.000702 |
| rs6002277 | G | C | chr22 | 41186918 | RARB | 3.19E-05 | 0.011087 | 0 | 0 |
| rs60332615 | C | A | chr3 | 52833579 | ZN148 | 0.000697 | 0.329103 | 0 | 0 |
| rs604228:G | C | G | chr6 | 64389931 | GABPA | 0.207357 | 0.000222 | 1.83E-06 | 0.028517 |
| rs604228:G | C | G | chr6 | 64389931 | NRF1 | 0.001061 | 4.67E-07 | 4.89E-07 | 0.007635 |
| rs60490177:A | G | A | chr3 | 37213434 | KLF15 | 0.838371 | 3.83E-05 | 2.93E-06 | 0.04574 |
| rs60490177:C | G | C | chr3 | 37213434 | KLF15 | 0.838371 | 5.33E-05 | 2.93E-06 | 0.04574 |
| rs60755037 | G | A | chr7 | 1990469 | NRF1 | 4.38E-07 | 0.941899 | 0 | 0 |
| rs60832053:G | T | G | chr3 | 37186242 | SIX5 | 0.030027 | 0.000158 | 1.97E-07 | 0.003068 |
| rs610060 | G | A | chr3 | 52239405 | NRF1 | 4.12E-07 | 0.001336 | 4.43E-07 | 0.006913 |
| rs61038964 | A | C | chr12 | 109996852 | ZN770 | 0 | 3.04E-07 | 6.27E-07 | 0.009789 |
| rs61160643 | T | C | chr8 | 88263731 | NFYA | 0.031144 | 0.000172 | 0 | 0 |
| rs615556 | G | C | chr11 | 57884592 | RARB | 3.19E-05 | 0.011087 | 0 | 0 |
| rs61660810 | C | T | chr17 | 20267295 | BATF::JUN | 6.62E-05 | 0.015981 | 0 | 0 |
| rs61684558 | G | A | chr1 | 150551801 | ZN770 | 0 | 1.19E-06 | 0 | 0 |
| rs61749865 | C | T | chr17 | 19931799 | JUN | 4.81E-05 | 0.071054 | 0 | 0 |
| rs61765513 | G | T | chr1 | 73425217 | ZNF460 | 1.74E-05 | 0.001454 | 1.14E-06 | 0.017762 |
| rs61787323 | C | T | chr1 | 98174185 | JUN | 0.01502 | 0.000239 | 0 | 0 |
| rs61787323 | C | T | chr1 | 98174185 | BATF::JUN | 0.037591 | 6.83E-05 | 0 | 0 |
| rs61787828:C | A | C | chr1 | 97921399 | SIX5 | 0.030027 | 0.000116 | 1.97E-07 | 0.003068 |
| rs61869836 | T | A | chr10 | 103354990 | RARB | 0.011087 | 3.19E-05 | 0 | 0 |
| rs61882691 | C | A | chr11 | 46417165 | JUN | 0.01502 | 0.000239 | 0 | 0 |
| rs61882691 | C | A | chr11 | 46417165 | BATF::JUN | 0.049162 | 6.83E-05 | 0 | 0 |
| rs61888875:G | C | G | chr11 | 57716201 | KLF12 | 0.000764 | 7.50E-06 | 1.82E-06 | 0.028406 |
| rs61888880 | C | T | chr11 | 57754155 | ZNF460 | 8.13E-09 | 2.38E-06 | 0 | 0 |
| rs61888880 | C | T | chr11 | 57754155 | ZN770 | 0 | 3.04E-07 | 6.27E-07 | 0.009789 |
| rs619091 | A | T | chr11 | 134014242 | ZN770 | 0 | 1.19E-06 | 0 | 0 |
| rs61955125:G | C | G | chr12 | 123406560 | ZN770 | 0 | 1.02E-06 | 0 | 0 |
| rs62027818 | T | C | chr15 | 83992393 | TFAP2E | 0.005605 | 0.000131 | 0 | 0 |
| rs62072050:G | C | G | chr17 | 18003250 | SIX5 | 0.021001 | 6.20E-05 | 1.97E-07 | 0.003068 |
| rs62233810 | A | G | chr3 | 2435326 | NRF1 | 0.004292 | 5.33E-07 | 3.54E-07 | 0.005519 |
| rs62244383:C | G | C | chr3 | 31964409 | ZN770 | 0 | 1.19E-06 | 0 | 0 |
| rs62291406:A | C | A | chr3 | 180896214 | SP1 | 0.000971 | 0.790826 | 2.43E-06 | 0.037888 |
| rs62291407 | G | T | chr3 | 180896430 | FOXK1 | 0.00048 | 0.079985 | 0 | 0 |
| rs62346178 | A | G | chr4 | 169699661 | ZN770 | 0 | 1.02E-06 | 0 | 0 |
| rs62365944 | C | T | chr5 | 50469920 | NFYB | 0.000109 | 0.631315 | 5.73E-07 | 0.008936 |
| rs642173 | A | G | chr6 | 64383054 | ZN770 | 3.33E-07 | 0 | 0 | 0 |
| rs6429630:C | T | C | chr1 | 43642106 | PITX1 | 0.15655 | 2.42E-06 | 0 | 0 |
| rs6439649:A | G | A | chr3 | 136652849 | RFX2 | 1.17E-06 | 0.037194 | 0 | 0 |
| rs6439649:C | G | C | chr3 | 136652849 | RFX2 | 1.17E-06 | 0.112242 | 0 | 0 |
| rs6439649:T | G | T | chr3 | 136652849 | RFX2 | 1.17E-06 | 0.089132 | 0 | 0 |
| rs6451796 | C | T | chr5 | 45356532 | FOXK1 | 0.071129 | 0.000375 | 0 | 0 |
| rs6454776 | G | A | chr6 | 64297710 | FOS::JUNB | 0.00942 | 6.70E-05 | 0 | 0 |
| rs6454776 | G | A | chr6 | 64297710 | BACH2 | 0.080963 | 7.37E-06 | 1.06E-06 | 0.016573 |
| rs6454776 | G | A | chr6 | 64297710 | BATF::JUN | 0.014534 | 2.39E-05 | 0 | 0 |
| rs6454776 | G | A | chr6 | 64297710 | JUN | 0.876541 | 1.09E-06 | 0 | 0 |
| rs6454776 | G | A | chr6 | 64297710 | JUN | 0.01502 | 0.000172 | 0 | 0 |
| rs6471803 | G | A | chr8 | 59754672 | FOXK1 | 0.079985 | 0.000436 | 0 | 0 |
| rs649504 | T | C | chr1 | 43640277 | FOXK1 | 0.000404 | 0.073812 | 0 | 0 |
| rs6497520 | G | A | chr16 | 9812361 | NFYA | 0.086603 | 0.000447 | 0 | 0 |
| rs6500604:G | C | G | chr16 | 4451654 | ZN770 | 0.096846 | 0 | 0 | 0 |
| rs6500604:G | C | G | chr16 | 4451654 | ZNF460 | 2.64E-05 | 3.37E-07 | 1.14E-06 | 0.017762 |
| rs6500604:T | C | T | chr16 | 4451654 | ZN770 | 0.096846 | 1.02E-06 | 0 | 0 |
| rs6502618 | A | G | chr17 | 17843427 | RFX2 | 6.22E-09 | 0.001883 | 0 | 0 |
| rs654825 | C | A | chr12 | 29783693 | SP1 | 2.30E-07 | 0.000352 | 2.43E-06 | 0.037888 |
| rs6550470 | A | G | chr3 | 37303735 | JUN | 5.62E-05 | 0.037147 | 0 | 0 |
| rs655293:T | G | T | chr12 | 123043858 | RFX2 | 0.000248 | 0.247683 | 1.51E-06 | 0.023553 |
| rs655293:T | G | T | chr12 | 123043858 | RFX2 | 1.03E-06 | 0.68135 | 0 | 0 |
| rs6574777 | C | G | chr14 | 84226459 | RFX2 | 0.010172 | 1.33E-06 | 0 | 0 |
| rs6577644:T | A | T | chr3 | 17725241 | NFYA | 0.08213 | 0.000249 | 0 | 0 |
| rs6579965 | T | G | chr5 | 140789226 | ZN770 | 1.19E-06 | 0 | 0 | 0 |
| rs6604098:T | C | T | chr1 | 97939476 | PITX1 | 2.42E-06 | 0.15655 | 0 | 0 |
| rs66657210 | T | C | chr4 | 169458484 | ZNF143 | 0.020542 | 0.00038 | 9.93E-07 | 0.015503 |
| rs6678194 | G | T | chr1 | 73425398 | ZNF143 | 0.000128 | 0.020542 | 9.93E-07 | 0.015503 |
| rs6686238:G | C | G | chr1 | 43744582 | FOXK1 | 0.000436 | 0.076615 | 0 | 0 |
| rs6700009 | A | C | chr1 | 150337684 | BATF::JUN | 6.83E-05 | 0.051608 | 0 | 0 |
| rs6700009 | A | C | chr1 | 150337684 | JUN | 0.000239 | 0.01502 | 0 | 0 |
| rs6700009 | A | C | chr1 | 150337684 | ZN770 | 0 | 3.04E-07 | 6.27E-07 | 0.009789 |
| rs6700607 | T | G | chr1 | 150331637 | PITX1 | 2.42E-06 | 0.15655 | 0 | 0 |
| rs6768079 | A | G | chr3 | 162054616 | ZN770 | 1.15E-06 | 0 | 0 | 0 |
| rs6768079 | A | G | chr3 | 162054616 | ZNF460 | 8.62E-07 | 8.13E-09 | 1.14E-06 | 0.017762 |
| rs67753165 | T | A | chr7 | 105020957 | FOXK1 | 0.000436 | 0.079985 | 0 | 0 |
| rs6778329 | G | A | chr3 | 52790594 | ZN148 | 0.000827 | 0.329103 | 0 | 0 |
| rs6786584 | T | C | chr3 | 37184237 | NRF1 | 0.001336 | 5.33E-07 | 4.43E-07 | 0.006913 |
| rs6786600 | G | A | chr3 | 162047471 | NRF1 | 0.041139 | 4.83E-06 | 1.40E-07 | 0.002183 |
| rs6830407 | A | G | chr4 | 102895366 | GABPA | 0.222657 | 0.000105 | 1.83E-06 | 0.028517 |
| rs6845516 | G | A | chr4 | 169724268 | SP1 | 4.98E-05 | 0.056571 | 2.43E-06 | 0.037888 |
| rs6853233 | G | A | chr4 | 102923292 | FOXK1 | 0.079985 | 0.00043 | 0 | 0 |
| rs6874127 | A | G | chr5 | 45305513 | JUN | 2.43E-05 | 0.01809 | 0 | 0 |
| rs6884426:A | T | A | chr5 | 50492782 | FOXK1 | 0.000436 | 0.079985 | 0 | 0 |
| rs6884426:C | T | C | chr5 | 50492782 | FOXK1 | 0.000436 | 0.079985 | 0 | 0 |
| rs6894784 | A | G | chr5 | 45710935 | NRF1 | 0.938579 | 4.26E-06 | 0 | 0 |
| rs6900902:T | A | T | chr6 | 104787882 | JUN | 0.029334 | 2.43E-05 | 0 | 0 |
| rs6936518 | G | A | chr6 | 64281288 | JUN | 0.022934 | 2.43E-05 | 0 | 0 |
| rs6937254 | G | A | chr6 | 108691257 | TBX15 | 9.37E-07 | 0.000156 | 0 | 0 |
| rs6957894:C | G | C | chr7 | 1847726 | GABPA | 0.070091 | 6.94E-05 | 1.83E-06 | 0.028517 |
| rs6970034:A | G | A | chr7 | 1908452 | BATF::JUN | 0.044437 | 0.000111 | 0 | 0 |
| rs6970034:A | G | A | chr7 | 1908452 | BACH2 | 0.077818 | 1.14E-05 | 1.06E-06 | 0.016573 |
| rs6987131:C | T | C | chr8 | 59804889 | JUN | 0.022934 | 1.03E-06 | 0 | 0 |
| rs7016434 | T | C | chr8 | 38200094 | ZNF460 | 6.40E-05 | 9.43E-07 | 1.14E-06 | 0.017762 |
| rs704364 | A | G | chr3 | 63889058 | JUN | 2.56E-05 | 0.016849 | 0 | 0 |
| rs7074749 | C | T | chr10 | 103356299 | PITX1 | 2.42E-06 | 0.15655 | 0 | 0 |
| rs7096249 | G | A | chr10 | 102858767 | SMC3 | 0.000223 | 0.079663 | 4.78E-08 | 0.000746 |
| rs7137243 | C | T | chr12 | 123332534 | ZN770 | 0 | 1.72E-06 | 0 | 0 |
| rs71408818:A | C | A | chr15 | 83822010 | SP1 | 2.33E-06 | 0.000574 | 1.48E-06 | 0.023039 |
| rs71408818:G | C | G | chr15 | 83822010 | SP1 | 2.33E-06 | 0.001187 | 6.81E-07 | 0.010634 |
| rs71408818:T | C | T | chr15 | 83822010 | SP1 | 2.33E-06 | 0.000882 | 6.81E-07 | 0.010634 |
| rs71655410 | C | G | chr1 | 73495471 | SP1 | 5.78E-06 | 0.341174 | 7.90E-07 | 0.012325 |
| rs7166248:G | A | G | chr15 | 82671937 | ZNF460 | 3.37E-07 | 8.12E-10 | 0 | 0 |
| rs7183881 | A | G | chr15 | 82671819 | ZN770 | 1.19E-06 | 0 | 0 | 0 |
| rs7191234:G | C | G | chr16 | 13707531 | NRF1 | 0.203417 | 4.12E-07 | 0 | 0 |
| rs7191664:C | T | C | chr16 | 4491923 | ZN770 | 3.33E-07 | 0 | 0 | 0 |
| rs7204624 | A | G | chr16 | 4409833 | RFX2 | 2.01E-06 | 0.012483 | 0 | 0 |
| rs7204852:A | C | A | chr16 | 30028857 | SP1 | 1.81E-06 | 0.000431 | 1.48E-06 | 0.023039 |
| rs7204852:G | C | G | chr16 | 30028857 | SP1 | 1.81E-06 | 0.000729 | 6.81E-07 | 0.010634 |
| rs7214919 | A | G | chr17 | 1430496 | PITX1 | 2.30E-05 | 0.268551 | 0 | 0 |
| rs7218819:C | T | C | chr17 | 1456386 | ZN148 | 0.329103 | 0.000521 | 0 | 0 |
| rs72487570 | T | C | chr12 | 123300843 | PITX1 | 0.268551 | 2.30E-05 | 0 | 0 |
| rs72720790 | G | A | chr1 | 177289737 | JUN | 3.36E-05 | 0.068565 | 0 | 0 |
| rs72720790 | G | A | chr1 | 177289737 | BATF::JUN | 6.62E-05 | 0.022437 | 0 | 0 |
| rs72802877 | A | G | chr5 | 152862295 | FOXK1 | 0.000436 | 0.076615 | 0 | 0 |
| rs72802883 | A | C | chr5 | 152867047 | JUN | 1.09E-06 | 0.005663 | 0 | 0 |
| rs72802893 | A | G | chr5 | 152876625 | GABPA | 0.275121 | 0.000925 | 2.09E-06 | 0.03268 |
| rs72932296 | G | A | chr2 | 200154353 | GABPA | 0.050176 | 0.000209 | 2.09E-06 | 0.03268 |
| rs72974205:G | C | G | chr2 | 224543751 | ZN586 | 0.05604 | 7.05E-06 | 0 | 0 |
| rs730050:G | C | G | chr3 | 52314348 | SP1 | 0.158421 | 0.000882 | 1.77E-06 | 0.027553 |
| rs73019028 | G | C | chr1 | 150464807 | ZN502 | 0.384857 | 4.01E-05 | 0 | 0 |
| rs7306815 | T | C | chr12 | 110009450 | JUN | 1.48E-06 | 0.005663 | 0 | 0 |
| rs73176687:A | C | A | chr22 | 41386419 | RFX2 | 1.77E-06 | 0.010172 | 0 | 0 |
| rs73230075:A | G | A | chr3 | 136776913 | ZN770 | 0 | 1.19E-06 | 0 | 0 |
| rs73230075:T | G | T | chr3 | 136776913 | ZN770 | 0 | 4.28E-06 | 0 | 0 |
| rs73292845 | T | C | chr17 | 1341596 | JUN | 0.101857 | 8.41E-07 | 0 | 0 |
| rs73536722 | C | A | chr19 | 30484088 | NFYA | 0.08213 | 0.000335 | 0 | 0 |
| rs736685:G | T | G | chr16 | 4458605 | ZN502 | 0.000504 | 0.932037 | 2.25E-06 | 0.035166 |
| rs740416 | G | A | chr12 | 2390726 | SOX2 | 0.971603 | 0.000481 | 0 | 0 |
| rs74301867:A | G | A | chr7 | 113996752 | FOXK1 | 0.071129 | 0.000404 | 0 | 0 |
| rs7438 | A | G | chr4 | 169721095 | JUN | 0.055937 | 3.18E-05 | 0 | 0 |
| rs7442762 | G | T | chr5 | 50358137 | RFX2 | 0.684763 | 8.97E-07 | 0 | 0 |
| rs7450304:T | G | T | chr6 | 111866450 | ZN770 | 2.84E-06 | 0 | 0 | 0 |
| rs74776107 | T | G | chr17 | 1370609 | MEF2A | 4.61E-06 | 0.370376 | 0 | 0 |
| rs7486723 | T | C | chr12 | 123279026 | RARB | 3.19E-05 | 0.011087 | 0 | 0 |
| rs75225286 | A | G | chr12 | 123318143 | KLF12 | 0.003151 | 2.80E-05 | 1.82E-06 | 0.028406 |
| rs7525762 | T | C | chr1 | 72906133 | GABPA | 0.222657 | 0.000105 | 1.83E-06 | 0.028517 |
| rs75319020:G | C | G | chr12 | 109925262 | ZN148 | 0.329103 | 0.000827 | 0 | 0 |
| rs75455415 | C | T | chr14 | 71925752 | GABPA | 0.000616 | 0.252522 | 2.09E-06 | 0.03268 |
| rs75543711 | G | A | chr17 | 1352780 | ZN770 | 0.529201 | 8.78E-05 | 0 | 0 |
| rs7558724 | A | G | chr2 | 104366872 | JUN | 4.59E-05 | 0.032262 | 0 | 0 |
| rs7559221 | C | A | chr2 | 134085723 | RFX2 | 0.003802 | 6.05E-07 | 0 | 0 |
| rs75721433 | C | G | chr17 | 19978747 | NRF1 | 0.007873 | 1.24E-06 | 4.43E-07 | 0.006913 |
| rs7574313 | C | G | chr2 | 199816317 | RFX2 | 1.54E-06 | 0.014138 | 0 | 0 |
| rs75756232 | G | C | chr2 | 232721313 | FOXK1 | 0.000449 | 0.079985 | 0 | 0 |
| rs757593 | A | C | chr16 | 4391616 | PITX1 | 2.30E-05 | 0.268551 | 0 | 0 |
| rs7612624 | T | C | chr3 | 52859410 | PITX1 | 2.42E-06 | 0.165614 | 0 | 0 |
| rs7615360 | T | G | chr3 | 36978459 | ZN770 | 1.02E-06 | 0 | 0 | 0 |
| rs76220111 | A | G | chr3 | 181152240 | SOX4 | 2.05E-05 | 0.013009 | 0 | 0 |
| rs76248139 | G | A | chr17 | 17989598 | SIX5 | 8.91E-05 | 0.028137 | 1.97E-07 | 0.003068 |
| rs7634972 | C | T | chr3 | 37164225 | MAX | 0.000101 | 0.035181 | 1.10E-06 | 0.01713 |
| rs7638808 | A | G | chr3 | 52538040 | BATF::JUN | 2.66E-05 | 0.016747 | 0 | 0 |
| rs7638808 | A | G | chr3 | 52538040 | JUN | 2.43E-05 | 0.022934 | 0 | 0 |
| rs7651664 | G | A | chr3 | 162098893 | FOXK1 | 0.073812 | 0.000331 | 0 | 0 |
| rs7663888 | C | A | chr4 | 175937870 | FOXK1 | 0.000375 | 0.06641 | 0 | 0 |
| rs77106645 | C | G | chr12 | 123316947 | ZN770 | 0 | 1.02E-06 | 0 | 0 |
| rs7728093:G | A | G | chr5 | 50540370 | EWSR1-FLI1 | 4.68E-07 | 1.51E-14 | 0 | 0 |
| rs7729211 | A | G | chr5 | 50412353 | MEF2A | 3.59E-05 | 0.430308 | 0 | 0 |
| rs77335224 | C | T | chr10 | 102876519 | ZN770 | 0 | 1.15E-06 | 0 | 0 |
| rs77335224 | C | T | chr10 | 102876519 | ZNF143 | 0.000993 | 0.024883 | 9.93E-07 | 0.015503 |
| rs77376275 | A | G | chr17 | 1384467 | ZN770 | 1.19E-06 | 0 | 0 | 0 |
| rs7757969:G | T | G | chr6 | 111810829 | ZN586 | 7.70E-06 | 0.025282 | 0 | 0 |
| rs7762381 | T | C | chr6 | 111912290 | ZN770 | 1.72E-06 | 0 | 0 | 0 |
| rs77627697 | G | A | chr14 | 84326233 | RFX2 | 0.011807 | 1.54E-06 | 0 | 0 |
| rs77756625 | C | A | chr10 | 63531947 | JUN | 0.130368 | 4.81E-05 | 0 | 0 |
| rs77810433 | A | G | chr3 | 36822409 | RARB | 4.03E-05 | 0.012489 | 0 | 0 |
| rs7788115 | A | T | chr7 | 86843015 | JUN | 1.09E-06 | 0.029334 | 0 | 0 |
| rs7789569 | T | C | chr7 | 105287139 | PITX1 | 2.30E-05 | 0.268551 | 0 | 0 |
| rs78005057 | C | T | chr10 | 63333623 | SMC3 | 0.000117 | 0.067639 | 4.78E-08 | 0.000746 |
| rs78037592 | G | T | chr2 | 200241011 | FOXK1 | 0.000375 | 0.06641 | 0 | 0 |
| rs7804907:T | C | T | chr7 | 86798892 | FOXK1 | 0.079985 | 0.000436 | 0 | 0 |
| rs7820821:A | C | A | chr8 | 110523388 | BATF::JUN | 0.025851 | 6.62E-05 | 0 | 0 |
| rs783536:A | T | A | chr15 | 82584034 | ZN770 | 7.13E-07 | 0 | 0 | 0 |
| rs78354120:G | C | G | chr6 | 30528492 | ZN148 | 0.329103 | 0.000697 | 0 | 0 |
| rs783545:G | C | G | chr15 | 82574513 | RARB | 0.012489 | 4.03E-05 | 0 | 0 |
| rs7843531 | G | A | chr8 | 88303186 | SP1 | 2.30E-07 | 0.076716 | 2.56E-07 | 0.004003 |
| rs7843531 | G | A | chr8 | 88303186 | SP1 | 2.33E-06 | 0.003105 | 2.61E-07 | 0.004078 |
| rs7843531 | G | A | chr8 | 88303186 | SMC3 | 0.000117 | 0.067639 | 4.78E-08 | 0.000746 |
| rs7843531 | G | A | chr8 | 88303186 | KLF12 | 7.50E-06 | 0.00106 | 1.82E-06 | 0.028406 |
| rs7843531 | G | A | chr8 | 88303186 | SP1 | 1.42E-07 | 4.13E-05 | 1.77E-06 | 0.027553 |
| rs7848263:C | A | C | chr9 | 82379894 | JUN | 1.09E-06 | 0.005974 | 0 | 0 |
| rs7902804:T | A | T | chr10 | 102825648 | ZN770 | 0 | 7.10E-07 | 0 | 0 |
| rs7909591 | G | T | chr10 | 102899261 | PITX1 | 2.30E-05 | 0.268551 | 0 | 0 |
| rs7921655 | C | T | chr10 | 103353944 | RARB | 3.19E-05 | 0.011087 | 0 | 0 |
| rs7949190 | A | G | chr11 | 124726845 | ZN770 | 1.19E-06 | 0 | 0 | 0 |
| rs7955408 | G | C | chr12 | 109899070 | ZN770 | 1.15E-06 | 0 | 0 | 0 |
| rs79599549 | A | G | chr4 | 23480853 | BATF::JUN | 0.007093 | 2.66E-05 | 0 | 0 |
| rs79665790 | G | A | chr12 | 123130285 | GABPA | 0.000691 | 0.203453 | 2.09E-06 | 0.03268 |
| rs79708380:A | G | A | chr12 | 29756524 | JUN | 0.876541 | 2.43E-05 | 0 | 0 |
| rs7974099:G | A | G | chr12 | 123286928 | NRF1 | 0.092629 | 8.02E-07 | 0 | 0 |
| rs79809573 | G | A | chr7 | 114053278 | BATF::JUN | 0.046388 | 0.000435 | 0 | 0 |
| rs80105733 | G | A | chr10 | 63100977 | ZN770 | 0 | 1.15E-06 | 0 | 0 |
| rs8021537 | C | T | chr14 | 84164203 | NFYA | 0.000203 | 0.034807 | 0 | 0 |
| rs8028363:A | T | A | chr15 | 82578136 | JUN | 0.071054 | 3.18E-05 | 0 | 0 |
| rs8070290:C | A | C | chr17 | 1428243 | ZNF460 | 1.11E-06 | 8.12E-10 | 0 | 0 |
| rs8077530:C | T | C | chr17 | 17950140 | JUN | 2.43E-05 | 0.019957 | 0 | 0 |
| rs812381:G | C | G | chr5 | 140685618 | ZNF460 | 3.71E-06 | 1.13E-08 | 0 | 0 |
| rs812381:G | C | G | chr5 | 140685618 | ZN770 | 0.096846 | 0 | 0 | 0 |
| rs812381:T | C | T | chr5 | 140685618 | ZN770 | 0.096846 | 1.15E-06 | 0 | 0 |
| rs8188289:A | C | A | chr5 | 50166394 | JUN | 0.068565 | 2.43E-05 | 0 | 0 |
| rs864975 | T | C | chr3 | 63899939 | JUN | 9.80E-07 | 0.624834 | 0 | 0 |
| rs866758:C | G | C | chr3 | 136399028 | FOXK1 | 0.079985 | 0.000436 | 0 | 0 |
| rs871527 | C | G | chr1 | 150489612 | NFYA | 0.000249 | 0.070321 | 0 | 0 |
| rs895882:C | T | C | chr2 | 199438292 | NFYB | 0.481453 | 0.000198 | 1.15E-06 | 0.017872 |
| rs917305:G | C | G | chr16 | 4390592 | ZN148 | 0.329103 | 6.08E-05 | 0 | 0 |
| rs925649 | C | T | chr3 | 37229373 | PITX1 | 2.42E-06 | 0.15655 | 0 | 0 |
| rs9261765:C | A | C | chr6 | 30405950 | USF1 | 0.129108 | 1.07E-05 | 2.51E-06 | 0.039228 |
| rs9261765:C | A | C | chr6 | 30405950 | USF1 | 0.007227 | 6.42E-06 | 0 | 0 |
| rs9261765:C | A | C | chr6 | 30405950 | MYCN | 0.001198 | 8.81E-07 | 7.05E-07 | 0.011003 |
| rs9261857:T | C | T | chr6 | 30416402 | TFAP2E | 7.81E-05 | 0.003525 | 0 | 0 |
| rs9261903 | C | T | chr6 | 30422800 | SP1 | 1.89E-05 | 0.016508 | 2.43E-06 | 0.037888 |
| rs9262174 | G | T | chr6 | 30762705 | ZNF460 | 3.37E-07 | 5.29E-05 | 4.50E-08 | 0.000702 |
| rs9262174 | G | T | chr6 | 30762705 | ZN770 | 0 | 2.84E-06 | 0 | 0 |
| rs9266683:A | G | A | chr6 | 31380687 | GABPA | 0.000287 | 0.238585 | 1.83E-06 | 0.028517 |
| rs9266683:T | G | T | chr6 | 31380687 | GABPA | 0.000287 | 0.238585 | 1.83E-06 | 0.028517 |
| rs9295865:A | G | A | chr6 | 30441607 | FOXK1 | 0.073812 | 0.000436 | 0 | 0 |
| rs9312452 | T | C | chr4 | 169595422 | JUN | 3.02E-05 | 0.022934 | 0 | 0 |
| rs9324383 | C | T | chr1 | 98020017 | BATF::JUN | 6.62E-05 | 0.013005 | 0 | 0 |
| rs9351225:A | T | A | chr6 | 64306094 | FOXK1 | 0.047756 | 0.000281 | 0 | 0 |
| rs9351268 | G | A | chr6 | 64470022 | USF2 | 1.49E-10 | 0.00037 | 0 | 0 |
| rs9356264:A | T | A | chr6 | 164736766 | ZNF460 | 8.12E-10 | 7.29E-08 | 1.14E-06 | 0.017762 |
| rs9377682:C | G | C | chr6 | 104903048 | SP1 | 0.033444 | 1.02E-05 | 1.05E-06 | 0.0164 |
| rs9377682:C | G | C | chr6 | 104903048 | KLF12 | 0.001919 | 1.57E-05 | 1.82E-06 | 0.028406 |
| rs9384804 | A | C | chr6 | 111755779 | JUN | 5.07E-05 | 0.079947 | 0 | 0 |
| rs9384809 | C | A | chr6 | 111821605 | RFX2 | 7.70E-09 | 0.002018 | 0 | 0 |
| rs9400240:A | G | A | chr6 | 108676408 | JUN | 0.297704 | 5.62E-05 | 0 | 0 |
| rs9451255:G | A | G | chr6 | 64283543 | FOXK1 | 0.000375 | 0.068764 | 0 | 0 |
| rs9451255:T | A | T | chr6 | 64283543 | FOXK1 | 0.000375 | 0.06641 | 0 | 0 |
| rs9461509 | G | A | chr6 | 29118882 | ZN148 | 5.28E-05 | 0.022075 | 0 | 0 |
| rs9468734:G | C | G | chr6 | 30433395 | SMC3 | 0.000494 | 0.094928 | 4.78E-08 | 0.000746 |
| rs9468734:T | C | T | chr6 | 30433395 | SMC3 | 0.000494 | 0.094928 | 4.78E-08 | 0.000746 |
| rs9468743 | C | T | chr6 | 30434647 | JUN | 0.038733 | 1.39E-06 | 0 | 0 |
| rs9501020 | A | G | chr6 | 30454295 | SMC3 | 0.067639 | 0.000154 | 4.78E-08 | 0.000746 |
| rs9611545 | G | C | chr22 | 41291661 | ZN770 | 0 | 0.565789 | 0 | 0 |
| rs9611567 | A | G | chr22 | 41373750 | JUN | 0.01502 | 0.000141 | 0 | 0 |
| rs9619935:G | C | G | chr22 | 41060009 | ZN770 | 0 | 1.19E-06 | 0 | 0 |
| rs9619935:T | C | T | chr22 | 41060009 | ZN770 | 0 | 1.15E-06 | 0 | 0 |
| rs9682667:C | T | C | chr3 | 50311906 | SP1 | 0.000241 | 0.045157 | 1.77E-06 | 0.027553 |
| rs9747590:G | A | G | chr17 | 1403793 | ZN770 | 1.02E-06 | 0 | 0 | 0 |
| rs9760180 | A | G | chr4 | 102904879 | ZNF460 | 2.40E-05 | 3.37E-07 | 1.14E-06 | 0.017762 |
| rs9820538 | C | T | chr3 | 63954833 | ZNF460 | 8.12E-10 | 3.37E-07 | 0 | 0 |
| rs9830582:A | C | A | chr3 | 17417340 | SP1 | 2.36E-07 | 8.20E-05 | 6.81E-07 | 0.010634 |
| rs9830582:G | C | G | chr3 | 17417340 | SP1 | 2.36E-07 | 0.000195 | 5.31E-07 | 0.008288 |
| rs9830582:T | C | T | chr3 | 17417340 | SP1 | 2.36E-07 | 0.000195 | 5.31E-07 | 0.008288 |
| rs9835523 | G | C | chr3 | 136424091 | RARB | 3.19E-05 | 0.011087 | 0 | 0 |
| rs9845788:C | G | C | chr3 | 136195873 | KLF12 | 7.50E-06 | 0.000915 | 1.82E-06 | 0.028406 |
| rs9845788:C | G | C | chr3 | 136195873 | ZN148 | 6.76E-06 | 0.006949 | 0 | 0 |
| rs9852386 | G | A | chr3 | 37180838 | ZNF460 | 8.12E-10 | 3.37E-07 | 0 | 0 |
| rs9853959:G | A | G | chr3 | 36809393 | ZNF460 | 3.37E-07 | 8.12E-10 | 0 | 0 |
| rs9865841 | A | G | chr3 | 136157283 | NFYA | 7.84E-05 | 0.010935 | 0 | 0 |
| rs9865841 | A | G | chr3 | 136157283 | NFYB | 6.24E-05 | 0.574619 | 0 | 0 |
| rs9876665:A | G | A | chr3 | 36806792 | USF2 | 1.81E-10 | 0.00037 | 0 | 0 |
| rs9890341:A | G | A | chr17 | 17919861 | ZN148 | 0.329103 | 0.000827 | 0 | 0 |
| rs9890341:C | G | C | chr17 | 17919861 | ZN148 | 0.329103 | 8.18E-05 | 0 | 0 |
| rs9908299:G | C | G | chr17 | 17871254 | PITX1 | 2.30E-05 | 0.268551 | 0 | 0 |
| rs9911121:A | G | A | chr17 | 1387854 | RARB | 3.19E-05 | 0.011087 | 0 | 0 |
| rs9911121:C | G | C | chr17 | 1387854 | RARB | 3.19E-05 | 0.011087 | 0 | 0 |
| rs9911850 | A | G | chr17 | 17993245 | ZNF460 | 9.81E-06 | 7.29E-08 | 4.50E-08 | 0.000702 |
| rs9912775 | C | T | chr17 | 20084167 | JUN | 0.022934 | 2.56E-05 | 0 | 0 |
| rs9916716:A | C | A | chr17 | 1377155 | ZN770 | 0 | 7.10E-07 | 0 | 0 |
| rs9923354:G | T | G | chr16 | 4400420 | RARB | 0.011087 | 3.19E-05 | 0 | 0 |
| rs9939774 | C | T | chr16 | 30057033 | ZN770 | 0 | 3.04E-07 | 6.27E-07 | 0.009789 |
| rs9939774 | C | T | chr16 | 30057033 | ZNF460 | 8.13E-09 | 1.84E-06 | 0 | 0 |
| rs9985697 | G | A | chr4 | 102913321 | REST | 0.055468 | 1.46E-05 | 1.45E-06 | 0.022629 |
|  |  |  |  |  |  |  |  |  |  |

**Supplementary Table 2. rs1399178 is significantly associated with schizophrenia.**

| **CHR** | **SNP** | **BP** | **A1/A2** | **FRQ** | **OR** | **SE** | **P** | **Dataset** | **Population** | **Case** | **Control** |
| --- | --- | --- | --- | --- | --- | --- | --- | --- | --- | --- | --- |
| 3 | rs1399178 | 37258669 | A/G | 0.27 | 1.018 | 0.008 | 3.75E-02 | MIX_PGC3 | Transancestry | 67390 | 94015 |
| 3 | rs1399178 | 37258669 | A/G | 0.434 | 1.02 | 0.009 | 2.40E-02 | EUR_PGC3 | European | 53386 | 77258 |
| 3 | rs1399178 | 37258669 | A/G | 0.327 | 1.031 | 0.01 | 2.92E-03 | MIX_2019 | Transancestry | 48223 | 60717 |
| 3 | rs1399178 | 37258669 | A/G | 0.431 | 1.036 | 0.011 | 1.27E-03 | EUR_2019 | European | 33640 | 43456 |
| 3 | rs1399178 | 37258669 | A/G | 0.065 | 0.988 | 0.034 | 7.20E-01 | EAS_2019 | East Asian | 14583 | 17261 |
| 3 | rs1399178 | 37258669 | A/G | 0.069 | 0.975 | 0.057 | 6.53E-01 | CHN_2017 | Chinese | 7699 | 18327 |


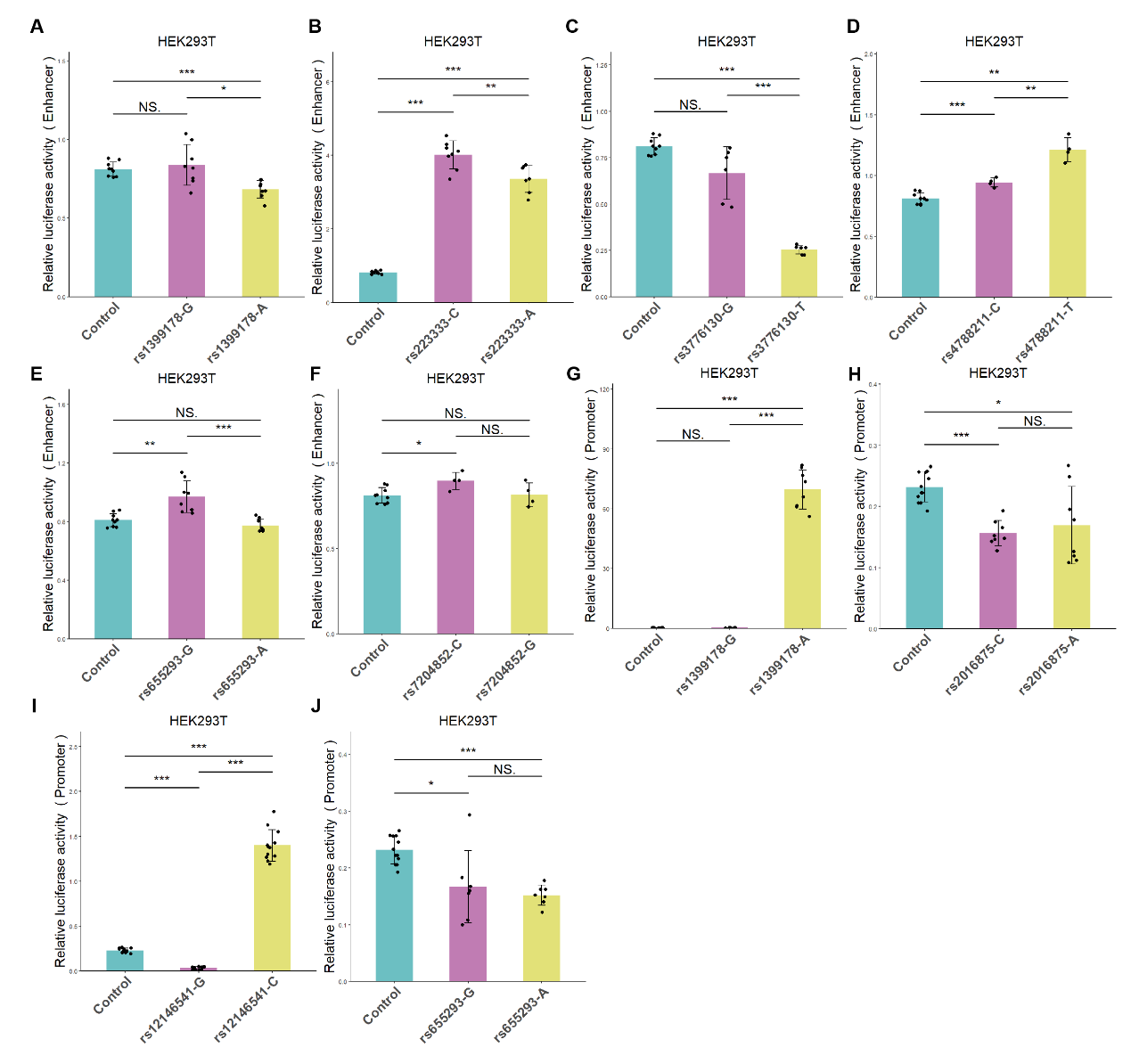


**Supplementary Figure 1. Validation of the regulatory effects of the TF binding‑disrupting SNPs with dual luciferase reporter gene assays.** DNA fragments containing different alleles of the test SNP were cloned into the corresponding vectors and luciferase activity were measured. Two-tailed Student’s t test was used to compare if the difference was significant. *P<0.05, **P<0.01, ***P<0.001. n=8 for each group.


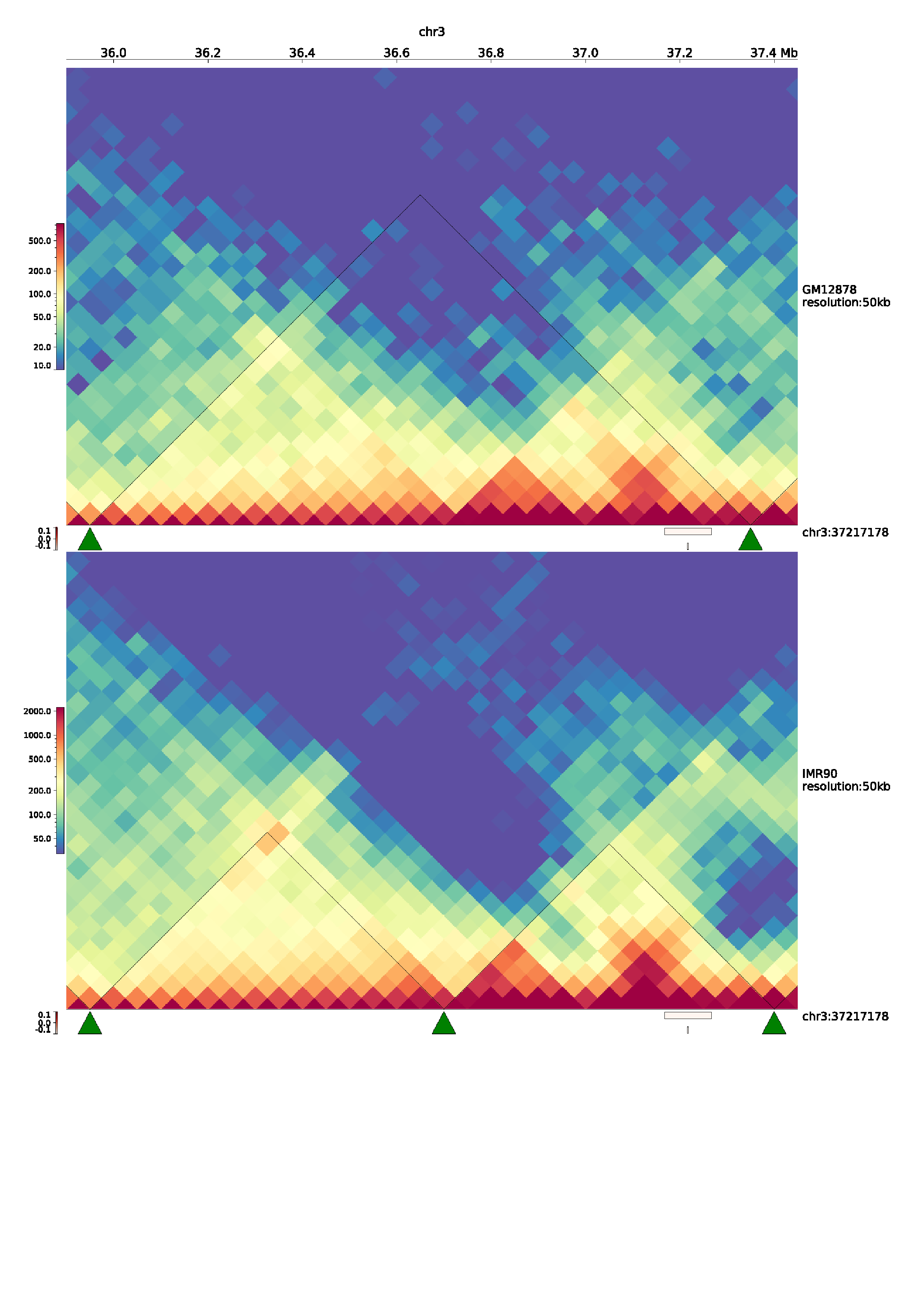


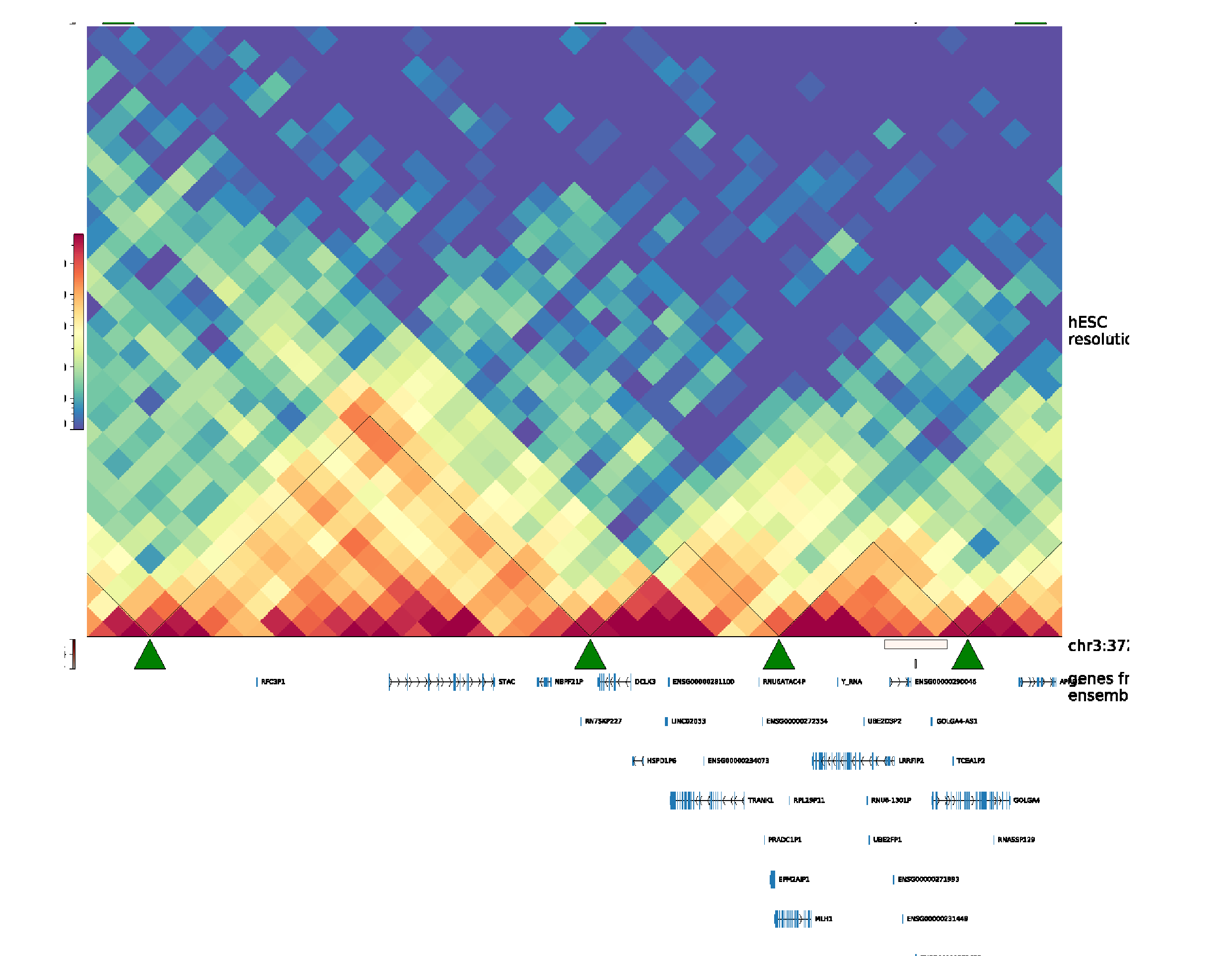


**Supplementary Figure 2. rs1399178 is located in a multi loop TAD defined using a Hi-C resolution of 50 kb.**


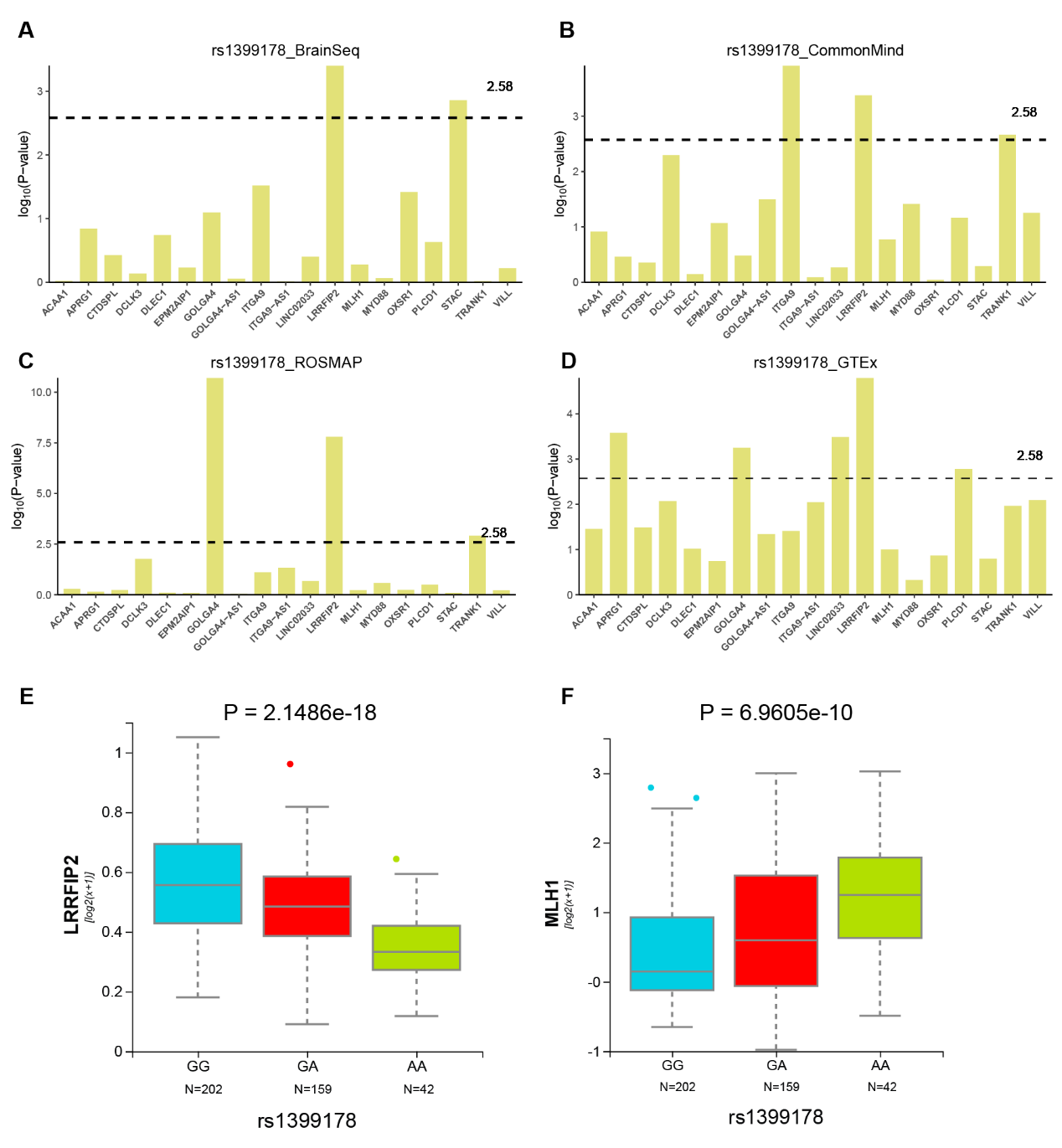


**Supplementary Figure 3. Association significance between rs1399178 and its nearby genes in the human brain.** (A-D) eQTL analysis of genes with rs1399178. Data were from the eQTL Catalogue (https://www.ebi.ac.uk/eqtl/). (E-F) The box-plot of eQTL anslysis. (Data from LIBD, n=403, https://eqtl.brainseq.org/phase1/eqtl/). A allele of rs1399178 corresponds to lower expression of LRRFIP2 and higher expression of MLH1.


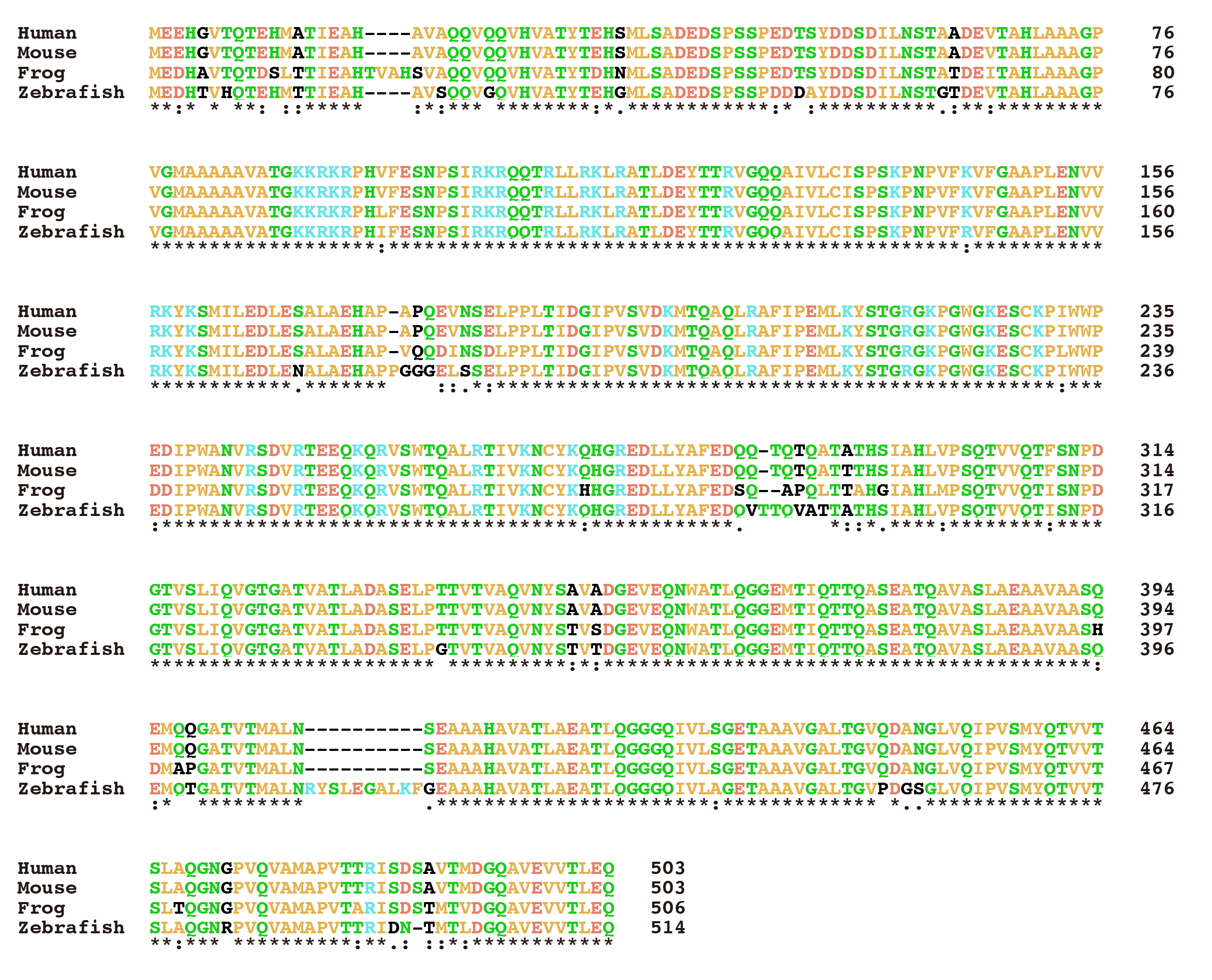


**Supplementary Figure 4. Sequence alignment of NRF1.**


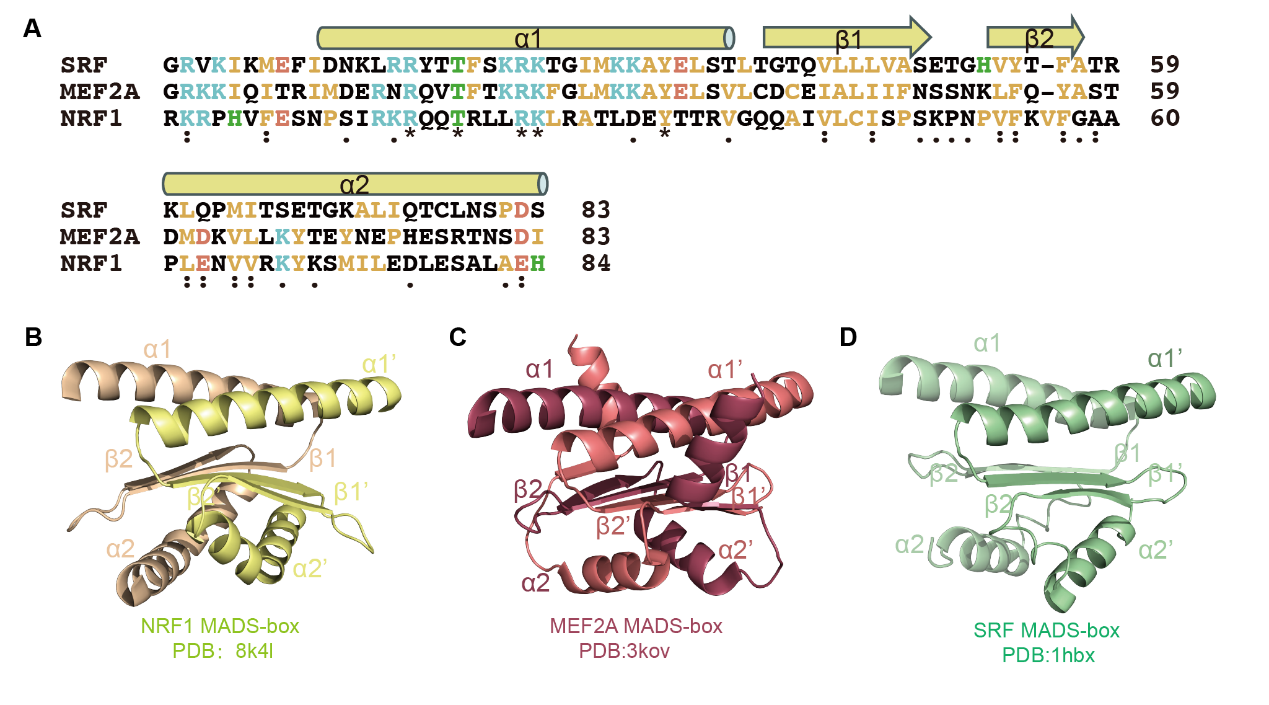


**Supplementary Figure 5: NRF1 comprises a MADS-box domain via dimerization.** (A) Sequence alignment of NRF1, MEF2A, and SRF within the MADS-box region. (B) Ribbon diagram illustrating the MADS-box domain of NRF1(PDB ID: 8k4l). (C) Ribbon diagram displaying the MADS-box of MEF2A (PDB ID: 3kov). (D) Ribbon diagram illustrating the MADS-box of SRF (PDB ID: 1hbx).


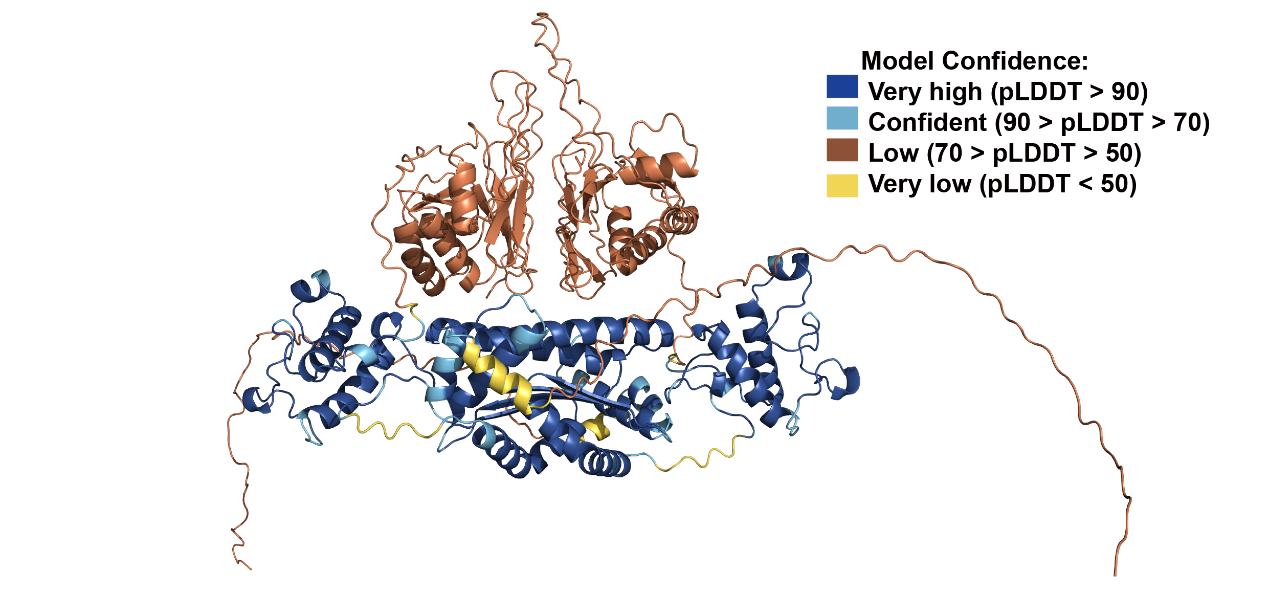


**Supplementary Figure 6. Predicted structure of NRF1 dimer by alphafold2.**


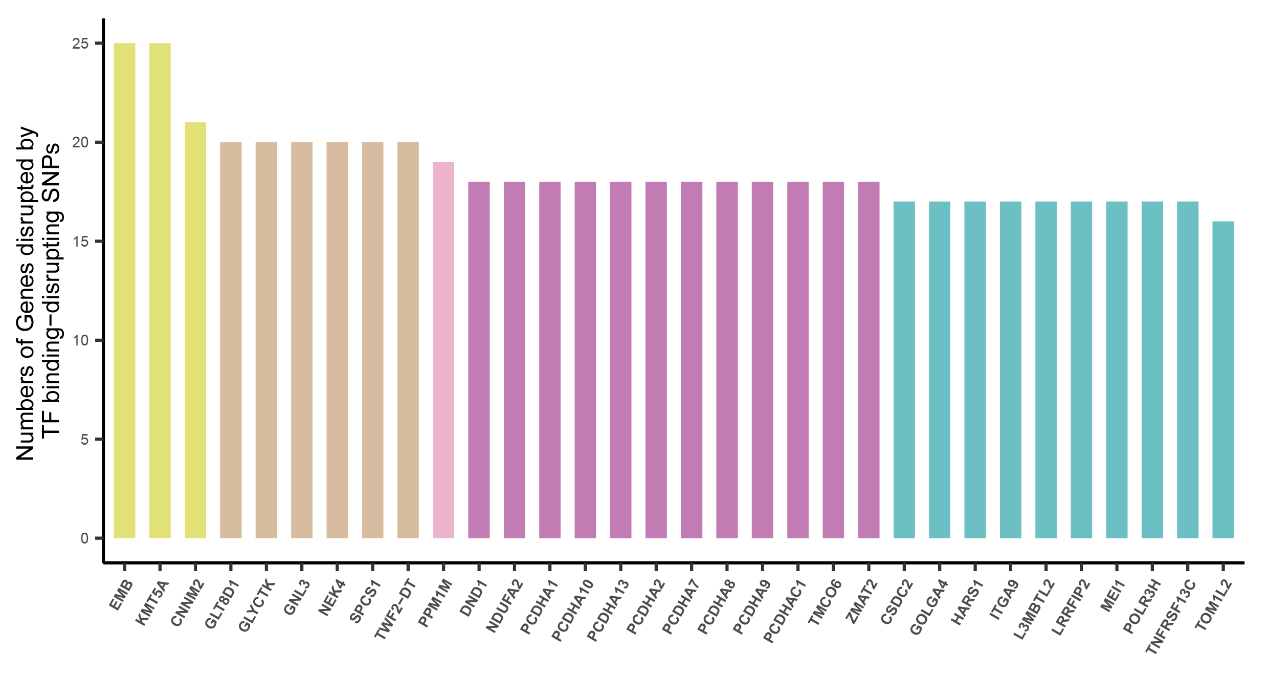


**Supplementary Figure 7. Numbers of Genes disrupted by TF binding-disrupting SNPs.**


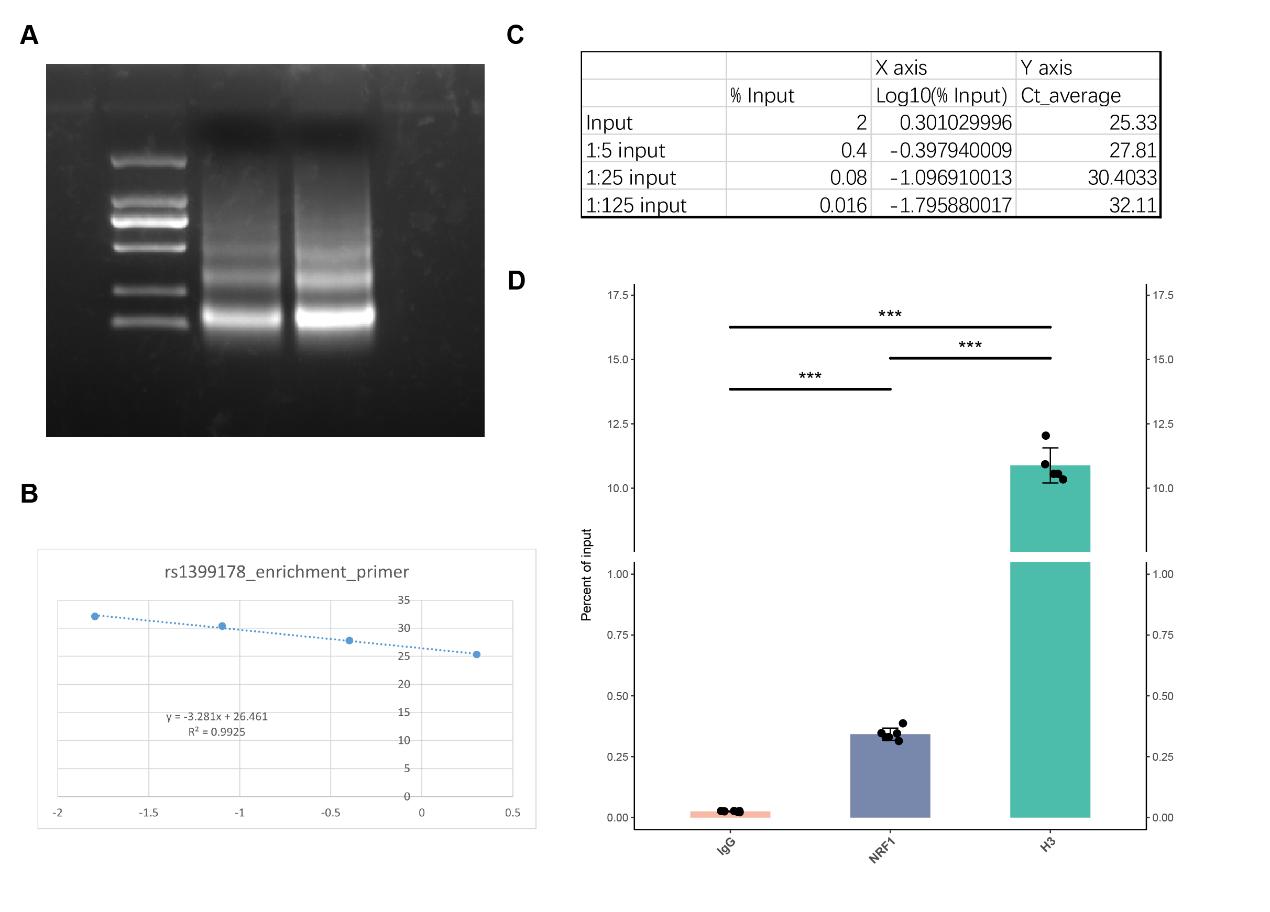


**Supplementary Figure 8. Experimental validation and efficiency analysis of Chip-qPCR Assay.** (A) depicts the electrophoresis pattern of DNA post-crosslinking, followed by enzymatic digestion and ultrasonic fragmentation. (B-C) qPCR standard curves were established by diluting input at different ratios, as recommended by CST, to determine the reaction efficiency of the qPCR assay. Our calculations indicate a qPCR reaction efficiency of 101.7368% for both primers and experimental conditions, falling within the recommended CST efficiency range of 90%-105%, affirming the suitability of both the reaction system and primers. (D) the results are computed using the percentage method.

**Detailed methods and primers are provided in this paper and include the following:**

GWAS used in this study

TFs and Chip-Seq experiments used in this study

Functional annotation of TF binding-disrupting SNPs Using RegulomeDB, Haploreg and 3DSNP

Cell culture

Vector construction

Dual Luciferase Reporter Gene Assays

Electrophoretic mobility shift assay

Supplementary Table 3. ChIP-Seq experiments and corresponding TFs from GTRD database using in this study.

Supplementary Table 4. RegulomeDB score and related functional annotation.

Supplementary Table 5. PCR primers used for amplification of DNA sequence containing TF binding-disrupting SNPs (for reporter gene assay).

Supplementary Table 6. ChIP-qPCR primer used for NRF1 enrichment analysis.

Supplementary Table 7. The probes used for EMSA.

Supplementary Table 8. R packages used in this study.

**Supplementary Methods**

**GWAS used in this study**

GWS schizophrenia risk SNPs identified by Li et al. were used in this study (11). Li et al. first carried out a GWAS approach in the Chinese population (containing 7699 cases and 18,327 controls). The authors then conducted a meta-analysis (including 43,175 cases and 65,166 controls) combining the associations obtained from the Chinese population and PGC2. Finally, an independent Chinese sample of 4384 cases and 5770 controls was used for the replication study. A total of 124 genome-wide significant (P < 5x10-8) SNPs were identified based on the combined results of the trans-ancestry meta-analysis and replication samples (See supplementary Table 3 for details).

**TFs and Chip-Seq experiments used in this study**

The functional genomics flow chart for identifying TF-disrupting SNPs is shown in Figure 1. Briefly, we first utilized 126 Chip-seq data from the Gene Transcription Regulation Database (GTRD, version 21.12), including only ChIP-seq experiments performed in human brain tissues or neuronal cells (including neuronal cell lines and iPSCs-derived neural progenitor cells) (29). The GTRD data was used for general analysis of TF binding, as it merges the binding sites for individual TFs into meta-clusters representing all experimental conditions (tissues, cell lines, treatments, etc.), reducing the computational resources required for analysis. To eliminate differences in software analysis, only Chip-seq peaks identified with MACS were used for PWM identification (30). The detailed information about the TFs and Chip-Seq experiments can be found in Supplementary Table 3.

**Functional annotation of TF binding-disrupting SNPs Using RegulomeDB, Haploreg and 3DSNP**

We employed three widely recognized online functional annotation tools to enrich for the most functional SNPs at each risk loci, including RegulomeDB (http://www.regulomedb.org/), HaploReg v4.1 (http://www.broadinstitute.org/mammals/haploreg/haploreg.php) and 3DSNP v2.0 (<https://omic.tech/3dsnpv2/>) [1-3]. RegulomeDB employs a scoring system to aid in the interpretation of putative regulatory variants, assessing their potential impact on transcription factor binding, enhancers, and promoters [1]. Variants are assigned scores on a scale from 1 to 6, with lower scores signifying a greater likelihood of functional significance (see Supplementary Table 4 for RegulomeDB score and related functional annotation). Here, the variants categorized under class one were considered as probable regulatory variants, while those falling within classes two to seven were considered unlikely to impact gene expression. Similarly, HaploReg serves as a tool for predicting the functional implications of genetic variations, encompassing aspects such as sequence conservation, regulatory protein binding, expression quantitative trait loci, regulatory motifs, and variant catalogs [2]. 3DSNP employs a quantitative scoring system to gauge the functionality of a SNP [3]. Within 3DSNP, each SNP is assigned a score based on six functional categories, including 3D interacting genes, enhancer state, promoter state, transcription factor binding sites, altered sequence motifs, and conservation score. Higher scores indicate more robust evidence supporting an SNP's regulatory role. All TF binding-disrupting SNPs identified in this study have been scored to evaluate the functional significance of SNPs using RegulomeDB, HaploReg and 3DSNP, one by one. We also investigated whether the variation in rs1399178 within Topologically Associating Domain (TAD) structures could be associated with gene regulation [4]. Briefly, we collected in-situ Hi-C data for the GM12878 (lymphoblastoid cells), IMR90 (fetal lung fibroblasts), and hESC (human embryonic stem cells) cell lines from the 4DN data portal (htps://data.4dnucleome.org/), and analyzed the TAD distribution with resolutions of 100, 50, and 25 kb [5, 6].

**Cell culture**

The HEK293T (human embryonic kidney cell line) and SH-SY5Y (human neuroblastoma cell line) used in this study were obtained from the Cell Bank Committee for Typical Cultures, Chinese Academy of Sciences. HEK293T cells were cultured in high-glucose Dulbecco's Modified Eagle Medium (DMEM) (Gibco, Cat. No: C11995500BT) supplemented with 10% fetal bovine serum (FBS) (Gibco, Cat. No: 10091148) and 1% penicillin-streptomycin (Beyotime, Cat. No: C0222). SH-SY5Y cells were cultured in complete media (Cat. No: SCSP-666) commercially available from the Chinese Academy of Sciences. Passaging of cells was performed when the cell density reached approximately 80 to 90% confluence, and the cell culture medium was refreshed every two days. The cells were cultured at 37 °C in 5% CO2.

**Vector construction**

Based on the genomic coordinates of the prioritized functional SNPs, the vectors employed in this study included the pGL4.11[luc2P] vector and the pGL3 promoter vector. The selection of vectors was contingent upon the genomic location of the target SNPs: pGL4.11[luc2P] vectors were utilized if the target SNPs were located in the promoter regions; otherwise, pGL3 promoter vectors were employed. In cases of significant SNPs, both vectors were concurrently utilized. Briefly, the DNA fragments (~300-800 bp) containing the target SNPs were amplified using clone primers (Supplementary Table 5). Each pair of clone primers featured specific sequences (homologous to the sequence of the reporter vector) at the 5' end. Subsequently, the vectors underwent digestion with the KpnI (NEB, R3142) and XhoI (NEB, R0146) restriction enzymes. The PCR products containing the target SNPs were then inserted into the vectors using 2×Hieff Clone® Enzyme Premix (Yeasen, Cat. No:10911). The ligated vectors were subsequently transformed into DH5α competent cells, and the sequence was obtained by Sanger sequencing. To introduce the alternative allele of the target SNP into the vector, PCR-mediated mutation was employed (Yeasen, Cat. No:11003). All cloned sequences were validated by Sanger sequencing.

**Dual Luciferase Reporter Gene Assays**

Dual-luciferase reporter gene assays are a valuable tool for assessing the regulatory effects of target DNA sequences [7]. We incorporated these target DNA sequences into the regulatory region, i.e., the promoter or enhancer, of the reporter gene vector (specifically the firefly luciferase). The evaluation of the regulatory effect was detected by quantifying the activity of firefly luciferase. HEK293T and SH-SY5Y cells were transfected with the constructed pGL3 promoter or pGL4.11[luc2P] vectors. The pRL-TK Renilla vector served as the internal control. HEK293T and SH-SY5Y cells were seeded into 96-well plates at densities of 3.5 × 10^4^ and 7.0 × 10^4^ cells/well, respectively. Following a 24-hour incubation period, we employed lipo8000TM (Beyotime, Cat.No: C0533) to transfect the vectors mentioned above. Cells were co-transfected with 100 ng of the pGL4.11[luc2P] or the pGL3 promoter, along with 20 ng of pRL-TK Renilla as the internal control. The luciferase activity was quantified 48 hours post-transfection using the Dual-Luciferase Reporter Gene Assay Kit (Yeasen, Cat.No: 11402ES80) according to manufacturer’s instructions. Statistical analysis employed the two-tailed Student’s t-test to assess the significance of differences in luciferase activity between cells transfected with vectors containing different alleles of the target SNP. The significance threshold value was set at *P* < 0.05.

**Chromatin Immunoprecipitation (ChIP)-qPCR**

ChIP-qPCR was conducted to assess the binding of transcription factors NRF1 to the genomic sequence encompassing rs1399178 in SH-SY5Y cells. The SimpleChIP Enzymatic Chromatin IP Kit (CST, Cat. No: #9003) was employed in accordance with manufacturer's instructions. Three immunoprecipitation (IP) groups were established using IgG (CST, Cat.No: 2729, RRID: AB_1031062), H3 (CST, Cat. No: 4620, RRID: AB_1904005), and NRF1 (Abcam, Cat.No: ab175932, RRID: AB_2629496) antibodies. Cross-linked chromatin fragments (eight μg) were used for the NRF1 antibody as the experimental group, IgG served as the negative control, and H3 as the positive control. DNA purification preceded subsequent qPCR analysis, for which primers were designed to span 100 bp up- and down-stream from rs1399178 (Supplementary Table 6).

**Electrophoretic mobility shift assay**

We employed the Electrophoretic Mobility Shift Assay (EMSA) to investigate the interaction between a DNA sequence (37bp) containing rs1399178 and nuclear proteins of SH-SY5Y cells. Nuclear extracts were obtained using the nuclear protein extraction kit (Beyotime, Cat. No: P0028) and quantified using the BCA protein assay kit (Pierce, Cat. No: 23227). Oligonucleotides harboring the G and A alleles of rs1399178 were labeled at their 3' ends with biotin using the EMSA probe biotin labeling kit (Beyotime, Cat. No: GS008). Single-strand oligonucleotides were annealed to form double-stranded complementary sequences (Supplementary Table 7). The EMSA procedure was conducted using the Chemiluminescent EMSA Kit (Thermo Scientific, Cat. No: 20148) in accordance with manufacturer’s instructions. Specifically, 100 fM probes were incubated with nuclear extracts for a duration of 20 minutes. Subsequently, the resulting incubation solution was subjected to electrophoresis on 5% non-denaturing polyacrylamide gels with 0.5 × Tris borate/EDTA, followed by electro-transfer to a nylon membrane (Roche, Cat. No: 11417240001). After UV cross-linking, the membrane underwent incubation with Streptavidin-HRP Conjugate, and images were captured using the Tanon 5200 chemiluminescent imaging system (Tanon, Shanghai, China).

**Supplementary Table 3. Validation of the regulatory effects of the TF binding‑disrupting SNPs with dual luciferase reporter gene assays.**

| Cell type | Experiment | GEO series | GEO sample | Peak caller | Uniprot | Gene |
| --- | --- | --- | --- | --- | --- | --- |
| BE2C | EXP066293 | GSE136451 | GSM4050759 | MACS2 | P61964 | WDR5 |
| BE2C | EXP066294 | GSE136451 | GSM4050760 | MACS2 | P61964 | WDR5 |
| BE2C | EXP066295 | GSE136451 | GSM4050761 | MACS2 | P61964 | WDR5 |
| BE2C | EXP049480 | GSE94822 | GSM2486155 | MACS2 | P61296 | HAND2 |
| BE2C | EXP049479 | GSE94822 | GSM2486153 | MACS2 | Q99453 | PHOX2B |
| BE2C | EXP010206 |  |  | MACS2 | P49711 | CTCF |
| BE2C | EXP049482 | GSE94822 | GSM2486158 | MACS2 | P04198 | MYCN |
| BE2C | EXP049484 | GSE94822 | GSM2486161 | MACS2 | Q13207 | TBX2 |
| BE2C | EXP049487 | GSE94822 | GSM2486170 | MACS2 | P23771 | GATA3 |
| BE2C | EXP049483 | GSE94822 | GSM2486159 | MACS2 | P61371 | ISL1 |
| brain prefrontal cortex region tissues | EXP063878 | GSE129039 | GSM3692177 | MACS2 | P10827 | THRA |
| cranial neural crest cells | EXP035243 | GSE70751 | GSM1817193 | MACS2 | P10589 | NR2F1 |
| cranial neural crest cells | EXP035244 | GSE70751 | GSM1817200 | MACS2 | P05549 | TFAP2A |
| D54 (H54, Human glioblastoma) | EXP010252 |  |  | MACS2 | P49711 | CTCF |
| D54 (H54, Human glioblastoma) | EXP001028 | GSE32692 | GSM808774 | MACS2 | P01106 | MYC |
| D54 (H54, Human glioblastoma) | EXP001027 | GSE32692 | GSM808772 | MACS2 | P49711 | CTCF |
| GBM39 (Human brain tumor) | EXP063904 | GSE131956 | GSM3832721 | MACS2 | Q9UQE7 | SMC3 |
| GBM39 (Human brain tumor) | EXP063905 | GSE131956 | GSM3832722 | MACS2 | Q9UQE7 | SMC3 |
| GBM39 (Human brain tumor) | EXP063902 | GSE131956 | GSM3832719 | MACS2 | P49711 | CTCF |
| GBM39 (Human brain tumor) | EXP063903 | GSE131956 | GSM3832720 | MACS2 | P49711 | CTCF |
| H9-derived neural crest cells | EXP030994 | GSE28874 | GSM714811 | MACS2 | P10589 | NR2F1 |
| H9-derived neural crest cells | EXP030995 | GSE28874 | GSM714812 | MACS2 | P24468 | NR2F2 |
| H9-derived neural crest cells | EXP030996 | GSE28874 | GSM902779 | MACS2 | P05549 | TFAP2A |
| H9-derived neural progenitors | EXP057843 | GSE108114 | GSM2890238 | MACS2 | O15550 | KDM6A |
| H9-derived neural progenitors | EXP057841 | GSE108114 | GSM2890228 | MACS2 | Q12888 | TP53BP1 |
| H9-derived neural progenitors | EXP057842 | GSE108114 | GSM2890232 | MACS2 | Q12888 | TP53BP1 |
| HBMEC (human brain microvascular endothelial cells) | EXP010026 |  |  | MACS2 | P49711 | CTCF |
| hNSC (neural stem cells) | EXP054747 | GSE57369 | GSM1381222 | MACS2 | Q9HCK8 | CHD8 |
| iPSCs-derived neural progenitor cells | EXP055685 | GSE84259 | GSM2230418 | MACS2 | Q13263 | TRIM28 |
| medulloblastoma | EXP011199 |  |  | MACS2 | P49711 | CTCF |
| neural cells | EXP044801 |  |  | MACS2 | Q15910 | EZH2 |
| neural cells | EXP039854 |  |  | MACS2 | P49711 | CTCF |
| neural cells | EXP044898 |  |  | MACS2 | O60216 | RAD21 |
| neural cells | EXP011137 |  |  | MACS2 | P21675 | TAF1 |
| neural cells | EXP010993 |  |  | MACS2 | Q13127 | REST |
| neural cells | EXP045297 |  |  | MACS2 | Q9UQE7 | SMC3 |
| neural cells | EXP011210 |  |  | MACS2 | Q13127 | REST |
| neural cells | EXP011209 |  |  | MACS2 | P21675 | TAF1 |
| neural cells | EXP044996 |  |  | MACS2 | Q09472 | EP300 |
| neural cells | EXP039914 |  |  | MACS2 | P50539 | MXI1 |
| neural progenitors | EXP035665 | GSE74814 | GSM1934421 | MACS2 | P20265 | POU3F2 |
| neural progenitors | EXP060779 | GSE122631 | GSM3494643 | MACS2 | P51531 | SMARCA2 |
| neural progenitors | EXP044994 |  |  | MACS2 | Q15910 | EZH2 |
| neural progenitors | EXP040290 |  |  | MACS2 | P49711 | CTCF |
| neural progenitors | EXP060773 | GSE122631 | GSM3494631 | MACS2 | P51532 | SMARCA4 |
| neural progenitors | EXP060785 | GSE122631 | GSM3732029 | MACS2 | P41225 | SOX3 |
| neural progenitors | EXP060782 | GSE122631 | GSM3732026 | MACS2 | P48431 | SOX2 |
| neural progenitors | EXP060770 | GSE122631 | GSM3486202 | MACS2 | P15408 | FOSL2 |
| neural progenitors | EXP045285 |  |  | MACS2 | Q15910 | EZH2 |
| neural progenitors | EXP035664 | GSE74814 | GSM1934417 | MACS2 | P20265 | POU3F2 |
| neural progenitors | EXP060776 | GSE122631 | GSM3494637 | MACS2 | P51532 | SMARCA4 |
| PFSK1 (primitive neuroectodermal tumor) | EXP010202 |  |  | MACS2 | Q13127 | REST |
| PFSK1 (primitive neuroectodermal tumor) | EXP044701 |  |  | MACS2 | Q96ST3 | SIN3A |
| PFSK1 (primitive neuroectodermal tumor) | EXP010462 |  |  | MACS2 | Q13127 | REST |
| PFSK1 (primitive neuroectodermal tumor) | EXP010319 |  |  | MACS2 | P21675 | TAF1 |
| PFSK1 (primitive neuroectodermal tumor) | EXP010440 |  |  | MACS2 | Q13127 | REST |
| PFSK1 (primitive neuroectodermal tumor) | EXP010606 |  |  | MACS2 | O15409 | FOXP2 |
| SH-SY5Y (neuroblastoma) | EXP034556 | GSE65664 | GSM1602667 | MACS2 | P23771 | GATA3 |
| SH-SY5Y (neuroblastoma) | EXP038298 | GSE93275 | GSM2449944 | MACS2 | O75364 | PITX3 |
| SH-SY5Y (neuroblastoma) | EXP000196 | GSE20673 | GSM518560 | MACS2 | Q9Y462 | ZNF711 |
| SH-SY5Y (neuroblastoma) | EXP057837 | GSE107335 | GSM2864936 | MACS2 | P06213 | INSR |
| SH-SY5Y (neuroblastoma) | EXP040383 | GSE101295 | GSM2870996 | MACS2 | P49711 | CTCF |
| SH-SY5Y (neuroblastoma) | EXP049704 | GSE96915 | GSM2545830 | MACS2 | P15884 | TCF4 |
| SH-SY5Y (neuroblastoma) | EXP010752 |  |  | MACS2 | P23769 | GATA2 |
| SH-SY5Y (neuroblastoma) | EXP057836 | GSE107335 | GSM2864935 | MACS2 | P06213 | INSR |
| SH-SY5Y (neuroblastoma) | EXP049703 | GSE96915 | GSM2545828 | MACS2 | P15884 | TCF4 |
| SH-SY5Y (neuroblastoma) | EXP061363 | GSE99227 | GSM2636314 | MACS2 | A6NHR9 | SMCHD1 |
| SK-N-MC (Askin tumor) | EXP010279 |  |  | MACS2 | O15409 | FOXP2 |
| SK-N-MC (Askin tumor) | EXP034090 | GSE61944 | GSM1517537 | MACS2 | Q01543 | FLI1 |
| SK-N-MC (Askin tumor) | EXP044825 |  |  | MACS2 | Q15910 | EZH2 |
| SK-N-MC (Askin tumor) | EXP049769 | GSE94275 | GSM2472121 | MACS2 | Q92922 | SMARCC1 |
| SK-N-SH (neuroblastoma) | EXP011077 |  |  | MACS2 | P23771 | GATA3 |
| SK-N-SH (neuroblastoma) | EXP010961 |  |  | MACS2 | P25490 | YY1 |
| SK-N-SH (neuroblastoma) | EXP011080 |  |  | MACS2 | Q15561 | TEAD4 |
| SK-N-SH (neuroblastoma) | EXP058187 | GSE115862 | GSM3192042 | MACS2 | O60216 | RAD21 |
| SK-N-SH (neuroblastoma) | EXP044831 |  |  | MACS2 | Q15910 | EZH2 |
| SK-N-SH (neuroblastoma) | EXP011222 |  |  | MACS2 | P25490 | YY1 |
| SK-N-SH (neuroblastoma) | EXP010994 |  |  | MACS2 | P61244 | MAX |
| SK-N-SH (neuroblastoma) | EXP011016 |  |  | MACS2 | P32519 | ELF1 |
| SK-N-SH (neuroblastoma) | EXP058186 | GSE115862 | GSM3192041 | MACS2 | P49711 | CTCF |
| SK-N-SH (neuroblastoma) | EXP011166 |  |  | MACS2 | Q06546 | GABPA |
| SK-N-SH (neuroblastoma) | EXP010983 |  |  | MACS2 | Q02078 | MEF2A |
| SK-N-SH (neuroblastoma) | EXP011067 |  |  | MACS2 | Q02078 | MEF2A |
| SK-N-SH (neuroblastoma) | EXP011025 |  |  | MACS2 | P17535 | JUND |
| SK-N-SH (neuroblastoma) | EXP010716 |  |  | MACS2 | Q13127 | REST |
| SK-N-SH (neuroblastoma) | EXP038090 | GSE90682 | GSM2410321 | MACS2 | Q9H4W6 | EBF3 |
| SK-N-SH (neuroblastoma) | EXP011065 |  |  | MACS2 | P19793 | RXRA |
| SK-N-SH (neuroblastoma) | EXP035012 | GSE69119 | GSM1693101 | MACS2 | P10276 | RARA |
| SK-N-SH (neuroblastoma) | EXP011194 |  |  | MACS2 | Q86T24 | ZBTB33 |
| SK-N-SH (neuroblastoma) | EXP010264 |  |  | MACS2 | Q13127 | REST |
| SK-N-SH (neuroblastoma) | EXP010968 |  |  | MACS2 | Q06546 | GABPA |
| SK-N-SH (neuroblastoma) | EXP010941 |  |  | MACS2 | P23771 | GATA3 |
| SK-N-SH (neuroblastoma) | EXP010391 |  |  | MACS2 | P21675 | TAF1 |
| SK-N-SH (neuroblastoma) | EXP045069 |  |  | MACS2 | Q09472 | EP300 |
| SK-N-SH (neuroblastoma) | EXP040246 |  |  | MACS2 | P49711 | CTCF |
| SK-N-SH (neuroblastoma) | EXP011130 |  |  | MACS2 | Q15561 | TEAD4 |
| SK-N-SH (neuroblastoma) | EXP011178 |  |  | MACS2 | Q08050 | FOXM1 |
| SK-N-SH (neuroblastoma) | EXP011118 |  |  | MACS2 | P17535 | JUND |
| SK-N-SH (neuroblastoma) | EXP044702 |  |  | MACS2 | Q96ST3 | SIN3A |
| SK-N-SH (neuroblastoma) | EXP044938 |  |  | MACS2 | O14647 | CHD2 |
| SK-N-SH (neuroblastoma) | EXP038091 | GSE90682 | GSM2410322 | MACS2 | Q9H4W6 | EBF3 |
| SK-N-SH (neuroblastoma) | EXP064071 | GSE138295 | GSM4104637 | MACS2 | P01106 | MYC |
| SK-N-SH (neuroblastoma) | EXP011224 |  |  | MACS2 | P22415 | USF1 |
| SK-N-SH (neuroblastoma) | EXP011219 |  |  | MACS2 | Q99081 | TCF12 |
| SK-N-SH (neuroblastoma) | EXP011048 |  |  | MACS2 | Q08050 | FOXM1 |
| SK-N-SH (neuroblastoma) | EXP011001 |  |  | MACS2 | Q99081 | TCF12 |
| SK-N-SH (neuroblastoma) | EXP040104 |  |  | MACS2 | Q14653 | IRF3 |
| SK-N-SH (neuroblastoma) | EXP011040 |  |  | MACS2 | P15408 | FOSL2 |
| SK-N-SH (neuroblastoma) | EXP044720 |  |  | MACS2 | Q09472 | EP300 |
| SK-N-SH (neuroblastoma) | EXP011139 |  |  | MACS2 | P08651 | NFIC |
| SK-N-SH (neuroblastoma) | EXP011015 |  |  | MACS2 | P19793 | RXRA |
| SK-N-SH (neuroblastoma) | EXP035982 | GSE76815 | GSM2038347 | MACS2 | P49711 | CTCF |
| SK-N-SH (neuroblastoma) | EXP011115 |  |  | MACS2 | P40426 | PBX3 |
| SK-N-SH (neuroblastoma) | EXP045070 |  |  | MACS2 | Q9UQE7 | SMC3 |
| SK-N-SH (neuroblastoma) | EXP011100 |  |  | MACS2 | P32519 | ELF1 |
| SK-N-SH (neuroblastoma) | EXP040189 |  |  | MACS2 | Q9UKL0 | RCOR1 |
| SK-N-SH (neuroblastoma) | EXP010660 |  |  | MACS2 | Q13127 | REST |
| SK-N-SH (neuroblastoma) | EXP038092 | GSE90682 | GSM2410317 | MACS2 | Q9H4W6 | EBF3 |
| SK-N-SH (neuroblastoma) | EXP045068 |  |  | MACS2 | O60216 | RAD21 |
| SK-N-SH (neuroblastoma) | EXP011023 |  |  | MACS2 | P08651 | NFIC |
| SK-N-SH (neuroblastoma) | EXP039372 |  |  | MACS2 | Q15853 | USF2 |
| SK-N-SH (neuroblastoma) | EXP011104 |  |  | MACS2 | P40426 | PBX3 |
| SK-N-SH (neuroblastoma) | EXP011109 |  |  | MACS2 | P61244 | MAX |
| SK-N-SH (neuroblastoma) | EXP010972 |  |  | MACS2 | P22415 | USF1 |
| SK-N-SH (neuroblastoma) | EXP011183 |  |  | MACS2 | P15408 | FOSL2 |
| WA01 (H1 hESC)-derived neural progenitor cells | EXP064082 | GSE138812 | GSM4119830 | MACS2 | O15054 | KDM6B |

**Supplementary Table 4. RegulomeDB score and related functional annotation.**

| Score | Description |
| --- | --- |
| 1a | eQTL + TF binding + matched TF motif + matched DNase Footprint + DNase peak |
| 1b | eQTL + TF binding + any motif + DNase Footprint + DNase peak |
| 1c | eQTL + TF binding + matched TF motif + DNase peak |
| 1d | eQTL + TF binding + any motif + DNase peak |
| 1e | eQTL + TF binding + matched TF motif |
| 1f | eQTL + TF binding/DNase peak |
| 2a | TF binding + matched TF motif + matched DNase Footprint + DNase peak |
| 2b | TF binding + any motif + DNase Footprint + DNase peak |
| 2c | TF binding + matched TF motif + DNase peak |
| 3a | TF binding + any motif + DNase peak |
| 3b | TF binding + matched TF motif |
| 4 | TF binding + DNase peak |
| 5 | TF binding or DNase peak |
| 6 | Motif hit |

**Supplementary Table 5. PCR primers used for amplification of DNA sequence containing TF binding-disrupting SNPs (for reporter gene assay).**

| Primer name | PCR primer sequence (5’>3’) |
| --- | --- |
| rs1399178-pGL3-F | CCAGAACATTTCTCTATCGATAGGTACCCAAGGCGAATTTCATACTGG |
| rs1399178-pGL3-R | GATGCAGATCGCAGATCTCGAGCCTCCCTCTCGTACTGAT |
| rs1399178-pGL4.11-F | GCCTAACTGGCCGGTACCCAAGGCGAATTTCATACTGG |
| rs1399178-pGL4.11-R | GCCAGATCTTGATATCCTCGAGAGCCTCCCTCTCGTACTGAT |
| rs1399178-Mut-G>A-F* | GCAGCCACAGCTGCCCAAACTGC |
| rs1399178-Mut-G>A-R* | AGCTGTGGCTGCGCCCAGGAG |
| rs655293-pGL3-F | CCAGAACATTTCTCTATCGATAGGTACCGCATCGCTATGTAATGCCTC |
| rs655293-pGL3-R | GATGCAGATCGCAGATCTCGAGCTTAGACCCTAACTGCTGGG |
| rs655293-pGL4.11-F | GCCTAACTGGCCGGTACCGCATCGCTATGTAATGCCTC |
| rs655293-pGL4.11-R | GCCAGATCTTGATATCCTCGAGCTTAGACCCTAACTGCTGGG |
| rs655293-Mut-A>G-F* | TCAGCATGGCAACCTCAGTTGGGTGG |
| rs655293-Mut-A>G-R* | GGTTGCCATGCTGATAGCCACAGCCAG |
| rs12146541-pGL3-F | CCAGAACATTTCTCTATCGATAGGTACCATTAGAAAAGCCTGACAACTGCAG |
| rs12146541-pGL3-R | GATGCAGATCGCAGATCTCGAGGAACCACCCATCGCTTC |
| rs12146541-pGL4.11-F | CCTAACTGGCCGGTACCATTAGAAAAGCCTGACAACTGC |
| rs12146541-pGL4.11-R | GCCAGATCTTGATATCCTCGAGGGAACCACCCATCGCTTC |
| rs12146541-Mut-G>C-F* | GTTTGGCCGCAGATGGTGGCACACAACATTTAGC |
| rs12146541-Mut-G>C-R* | TGCGGCCAAACTCTCCTCCCTGGCAAC |
| rs223333-pGL3-F | CCAGAACATTTCTCTATCGATAGGTACCCTGCAGTTAAGTTGCCAAGG |
| rs223333-pGL3-R | TGCAGATCGCAGATCTCGAGCCTCGGAATCTCGTGTGAA |
| rs223333-Mut-A>C-F* | ACAGCACCCAAACTGTAGGGGAAGAGGCGGG |
| rs223333-Mut-A>C-R* | TTTGGGTGCTGTGGCCCTAGGGTTGCATACTCG |
| rs3776130-pGL3-F | CCAGAACATTTCTCTATCGATAGGTACCATCGTGAACAATAGGGCACA |
| rs3776130-pGL3-R | GATGCAGATCGCAGATCTCGAGACTGAACAACCCACCACTAC |
| rs3776130-Mut-G>T-F* | CCATCACTTTGGGAGGCCAAGGGTGGTGGATC |
| rs3776130-Mut-G>T-R* | CCCAAAGTGATGGGATTACAGGCATGAGCCACTGTGCC |
| rs4788211-pGL3-F | CCAGAACATTTCTCTATCGATAGGTACCCGGAGATGAGGGTAAGTGAG |
| rs4788211-pGL3-R | GATGCAGATCGCAGATCTCGAGCTGGGCATCCTGCTCTATTT |
| rs4788211-Mut-C>T-F* | TTTCCCGCTCCCCAGTGTCCTG |
| rs4788211-Mut-C>T-R* | GGAGCGGGAAACGGGGAGAAGTC |
| rs7204852-pGL3-F | CCAGAACATTTCTCTATCGATAGGTACCAACTCAATTCCTCTCGCTGA |
| rs7204852-pGL3-R | GATGCAGATCGCAGATCTCGAGTTCCCATAATCCAGGCATCC |
| rs7204852-Mut-C>G-F* | CCCCGCGCCTCCTGCCTGCACACC |
| rs7204852-Mut-C>G-R* | GAGGCGCGGGGACGGGGAGGAGAG |
| rs2016875-pGL4.11-F | GCCTAACTGGCCGGTACCGGGTTCTGACAAAATGAGT |
| rs2016875-pGL4.11-R | GCCAGATCTTGATATCCTCGAGGTAGTTATAGTCCAAGGGTGC |
| rs2016875-Mut-C>A-F* | CCTTGATTACTCATTTGAGGAGTTGTCATCCCC |
| rs2016875-Mut-C>A-R* | GAGTAATCAAGGTTCAGTTTGTCATGGTAATGGTT |

* represents primers for point mutation

**Supplementary Table 6. ChIP-qPCR primer used for NRF1 enrichment analysis.**

| Primer name | PCR primer sequence (5’>3’) |
| --- | --- |
| rs1399178-enrichment-F | AAGTTCTTGTCTCTGCAGGA |
| rs1399178-enrichment-R | CAGAGAGTGCAGAGATGCCT |

**Supplementary Table 7. The probes used for EMSA.**

| Primer name | PCR primer sequence (5’>3’) |
| --- | --- |
| rs1399178-G-EMSA-F | CACCCTCCTGGGCGCAGCCGCAGCTGCCCAAACTGCA |
| rs1399178-G-EMSA-R | TGCAGTTTGGGCAGCTGCGGCTGCGCCCAGGAGGGTG |
| rs1399178-A-EMSA-F | CACCCTCCTGGGCGCAGCCACAGCTGCCCAAACTGCA |
| rs1399178-A-EMSA-R | TGCAGTTTGGGCAGCTGTGGCTGCGCCCAGGAGGGTG |

The position of rs1399178 was marked with red

**Supplementary Table 8. R packages used in this study.**

| R package | version | URL | Description |
| --- | --- | --- | --- |
| LDlinkR | 1.3.0 | [https://ldlink.nih.gov](https://ldlink.nih.gov/) | Provides access to the 'LDlink' API (<https://ldlink.nih.gov/?tab=apiaccess>) using the R console. This programmatic access facilitates researchers who are interested in performing batch queries in 1000 Genomes Project (2015) <doi:10.1038/nature15393> data using 'LDlink'. 'LDlink' is an interactive and powerful suite of web-based tools for querying germline variants in human population groups of interest. For more details, please see Machiela et al. (2015) <doi:10.1093/bioinformatics/btv402>. |
| memes | 1.10.0 | <https://github.com/snystrom/memes> | A seamless interface to the MEME Suite family of tools for motif analysis. 'memes' provides data aware utilities for using GRanges objects as entrypoints to motif analysis, data structures for examining & editing motif lists, and novel data visualizations. 'memes' functions and data structures are amenable to both base R and tidyverse workflows |
| MotifDb | 1.44.0 | <https://git.bioconductor.org/packages/MotifDb> | More than 9900 annotated position frequency matrices from 14 public sources, for multiple organisms. |
| atSNP | 1.18.0 | <https://github.com/sunyoungshin/atSN> | atSNP performs affinity tests of motif matches with the SNP or the reference genomes and SNP-led changes in motif matches. |
